# Non-uniform sampling of similar NMR spectra and its application to studies of the interaction between alpha-synuclein and liposomes

**DOI:** 10.1007/s10858-023-00418-3

**Published:** 2023-05-26

**Authors:** Alexandra Shchukina, Thomas C. Schwarz, Michał Nowakowski, Robert Konrat, Krzysztof Kazimierczuk

**Affiliations:** 1 Faculty of Chemistry, University of Warsaw, Pasteura 1, 02-093 Warsaw, Poland; 2 Department of Structural and Computational Biology, Max Perutz Labs, University of Vienna, Vienna BioCenter Campus 5, 1030 Vienna, Austria; 3 Centre of New Technologies, University of Warsaw, Banacha 2C, 02-097 Warsaw, Poland

**Keywords:** Non-uniform sampling, Serial NMR, Compressed sensing, Alpha-synuclein, Temperature-gradient, Protein-membrane interaction

## Abstract

The accelerated acquisition of multidimensional NMR spectra using sparse non-uniform sampling (NUS) has been widely adopted in recent years. The key concept in NUS is that a major part of the data is omitted during measurement, and then reconstructed using, for example, compressed sensing (CS) methods. CS requires spectra to be compressible, that is, they should contain relatively few “significant” points. The more compressible the spectrum, the fewer experimental NUS points needed in order for it to be accurately reconstructed. In this paper we show that the CS processing of similar spectra can be enhanced by reconstructing only the differences between them. Accurate reconstruction can be obtained at lower sampling levels as the difference is sparser than the spectrum itself. In many situations this method is superior to “conventional” compressed sensing. We exemplify the concept of “difference CS” with one such case—the study of alpha-synuclein binding to liposomes and its dependence on temperature. To obtain information on temperature-dependent transitions between different states, we need to acquire several dozen spectra at various temperatures, with and without the presence of liposomes. Our detailed investigation reveals that changes in the binding modes of the alpha-synuclein ensemble are not only temperature-dependent but also show non-linear behavior in their transitions. Our proposed CS processing approach dramatically reduces the number of NUS points required and thus significantly shortens the experimental time.

**Supplementary Information:**

The online version contains supplementary material available at 10.1007/s10858-023-00418-3.

## Introduction

The analysis of biomolecular structure and dynamics by NMR usually requires the acquisition of many multidimensional (nD) spectra. Especially in the case of unfolded or intrinsically disordered proteins and those undergoing unfolding transitions, these measurements require extraordinarily high resolution. Thus, we have to perform extensive sampling of nD time-domain signals, which means that collecting data can take several days.

In this context, NMR-based techniques monitoring transitions between distinct structural states are of particular interest. Variable-pressure and variable-temperature NMR experiments give a unique insight into these transitions as they monitor the protein on a per-residue basis (Dreydoppel et al. 2022). This allows us to determine different transition temperatures for separate protein domains. Additionally, it makes it possible to confirm experimentally the “cooperativity” of the unfolding behavior. In the case of slow chemical exchange on the NMR timescale, these methodologies have additional advantages over other commonly used techniques, such as circular dichroism, differential scanning fluorimetry, differential scanning calorimetry, or dynamic light scattering: They allow for the residue-resolved monitoring of two or more distinct structural states (for example, folded, intermediate, or unfolded) (Zhang et al. 2019). In addition to the large amount of biophysical information that can be obtained through protein unfolding studies, specific temperature-dependent mechanisms are also often best addressed by variable-temperature NMR (Baxter et al. 1998; Shchukina et al. 2021). Variable-temperature NMR can also provide information on the thermodynamic properties of partially populated compact or exited states (Bouvignies et al. 2011), even in the context of intrinsically disordered proteins (IDPs) (Bah et al. 2014).

The study of liquid-liquid phase separation (LLPS) has recently attracted a great deal of scientific interest as phase-separating systems have been demonstrated to contribute strongly to cellular functions by their spatial arrangement of proteins. Due to the often intrinsically disordered nature of the protein regions involved and the fact that NMR is the predominant technique used to access IDPs on a per-residue basis, NMR is considered one of the best tools for studying LLPS systems (Murthy and Fawzi 2020). NMR also offers the advantage of being able to measure gradients of temperature, pH, pressure, and salt concentration, on which LLPS is highly dependent (Cinar et al. 2019).

**Fig. 1:**
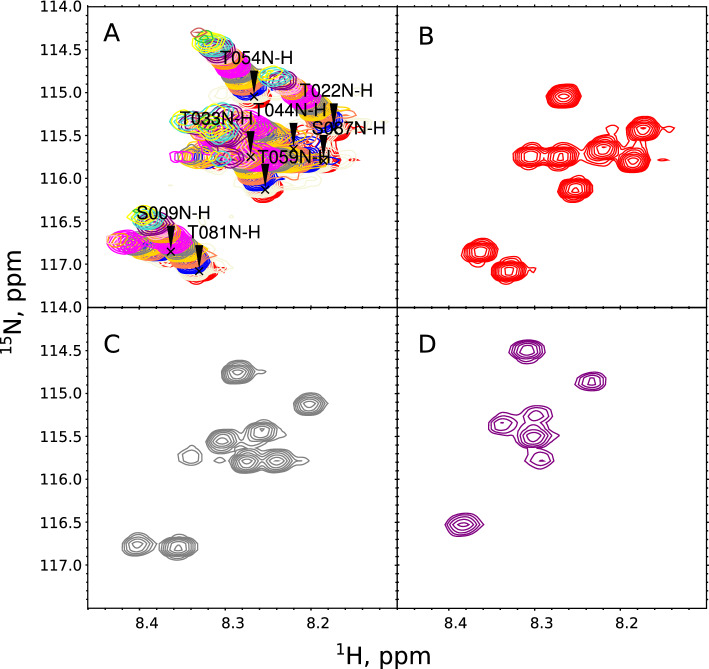
A region of 2D ${{}^{1}\hbox {H}}-{{}^{15}\hbox {N}}$ HSQC spectra of aSyn (without liposomes) measured at various temperatures. **A** Superimposed spectra at temperatures from 15 to 43 °C, with 2 °C increments. The resonance assignments are shown. **B** 15 °C. **C** 25 °C. **D** 35 °C. As can be seen, the assignment could not be unambiguously transferred between panels **B**, **C**, and **D** without the intermediate steps

**Fig. 2:**
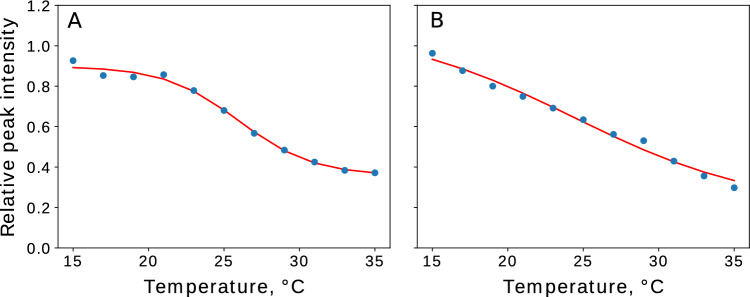
Relative heights (with vs. without liposomes) of example peaks in aSyn 2D ${{}^{1}\hbox {H}}-{{}^{15}\hbox {N}}$ HSQC spectra at various temperatures. **A** Peak A069N-H. The transition between two or more states is sigmoidal when fitted; we can estimate the “melting” temperature for this residue. **B** Peak T059N-H. The curve is almost linear, the decreased peak height resulting mostly from faster relaxation. If we sampled fewer temperature points, the approximation would be far less reliable. See profiles for all other residues in SI

When setting up an optimal variable-temperature series of NMR experiments, we need to decide on the temperature range and the sampling step, that is, how to sample the temperature “pseudo-dimension”. The sampling must be dense, for two main reasons. First, we can only transfer the resonance assignment between spectra in a series unambiguously if they do not differ too much (Fig. 1). If the experimental conditions cause dramatic changes between consecutive spectra, the resonance assignment must be re-established from the ground up. This is cumbersome as it requires multidimensional spectra and highly concentrated samples with appropriate isotopic labeling. Second, the temperature dependencies of peak intensities and chemical shifts can be very complex as they are influenced by many factors, such as relaxation, exchange with a solvent, changes in relative populations of structural states, sample degradation, and so on. Describing their complicated shapes is only possible with a sufficient number of points. Figure 2 shows examples of different peak intensity profiles from the current study.

It is interesting to note that similar problems appear in studies involving parameters other than temperature, for example, pressure (Xu et al. 2021), ligand concentration (Williamson 2018), and also various pulse-sequence settings such as mixing delay (Horst et al. 2006; Butts et al. 2011) and diffusion-encoding gradients (Pagès et al. 2017). To analyze the established dependencies unambiguously, many spectra must be acquired.

A sufficiently fine sampling of a temperature pseudo-dimension should typically include between 10 and 20 two-dimensional (2D) spectra, depending on the temperature range. Such measurements take many hours, even if sensitivity is high. For this, there are three main reasons. First, the Nyquist theorem (Nyquist 1928) states that the distance between sampling points is restricted by the spectral width. Second, because of the “Fourier uncertainty principle” (Szántay 2008), the evolution time must be sampled for long enough to maintain the spectral resolution. And third, samples need to return to equilibrium between consecutive sampling points in the indirect dimension, which takes several seconds unless fast-sampling techniques are used (Schanda et al. 2006). Because of these three factors, the measurement time can be as much as tens of hours, even for a single 2D spectrum (Misiak et al. 2013).

One of the most popular methods for accelerating NMR experiments is compressed sensing (CS). CS is based on the assumption of spectrum sparsity, whereby only a small proportion of spectral points contribute significantly to peaks; the rest contribute to the baseline and their intensity is close to zero. CS theory says that in such cases we can skip a considerable number of sampling points (Foucart and Rauhut 2010), a procedure called “non-uniform sampling” (NUS). Later on, we can reliably reconstruct the missing data points with one of several algorithms that exploit the sparsity assumption (Kazimierczuk and Orekhov 2011; Holland et al. 2011; Hyberts et al. 2012; Qu et al. 2015). CS theory further states that, for the probability of a good reconstruction of *N* spectral points to be high, the number *M* of sampled points should be proportional to

1 $$\begin{aligned} M \sim K \log (N/K) \end{aligned}$$

where *K* is the number of significant points in a spectrum (Foucart and Rauhut 2010; Shchukina et al. 2017a).

It should be noted that NMR spectra are not strictly sparse in the Fourier domain. CS theory states that in such cases *K* highest points in the spectrum may still be recovered exactly (see Theorem 2 in Candès et al. (2006)). Moreover, certain methods related to CS and based on low-rank minimization overcome this limitation by defining a strictly sparse representation of an NMR signal (Qu et al. 2015; Guo et al. 2023).

Usually, the reconstruction is performed column-wise for 2D NMR spectra, that is to say, separately for each point of the direct dimension, which has first undergone Fourier transform (FT). *K* varies from column to column, whereas *M* has to be the same for all the columns due to the way the 2D NMR signal is acquired. To be on the safe side, we should take into account the worst-case scenario in our choice of *M* and select the *K* of the least sparse (most crowded) column. If condition Eq. (1) is not fulfilled, peak intensities will be suppressed, which is particularly harmful when performing quantitative studies such as NOESY analysis (Wieske and Erdélyi 2021).

Typically, the 2D ${{}^{1}\hbox {H}}-{{}^{15}\hbox {N}}$ HSQC of even a small protein is not very sparse, so non-uniform sampling (NUS) of such spectra does not save much time (Shchukina et al. 2017a). The situation is worse for IDPs, which have very low peak dispersion in the ${{}^{1}\hbox {H}}$ dimension.

One way to improve CS efficiency is to increase the sparsity of the spectra. This can be done during signal acquisition or by means of pre-reconstruction signal processing. Specific methods include using a virtual echo (Mayzel et al. 2014) or reducing the number of peaks, for example, by using pure-shift (Aguilar and Kenwright 2018) or selective excitation (Piai et al. 2016). We have discussed several sparsity-increasing approaches elsewhere (Gołowicz et al. 2020).

In this paper we increase sparsity at the stage of signal processing, before the reconstruction. Our method involves exploiting the similarity between the reconstructed spectrum and a reference spectrum. We perform the CS reconstruction on the difference between two spectra, which is much sparser than either of the original ones. This concept has been successfully applied previously in real-time dynamic magnetic resonance imaging reconstruction by Majumdar et al. (2012).

Taking the difference between two spectra is an inherent part of other techniques, such as saturation transfer difference (Viegas et al. 2011) and difference diffusion-ordered spectroscopy (Ribeiro et al. 2010). It has also been used to remove background signals (de Groot et al. 1988). However, it has not been used before to support the CS reconstruction of spectroscopic NUS data sets. Some other methods of fast spectral acquisition have exploited the similarities between spectra in a series, but in different ways - methods such as multidimensional decomposition (Jaravine et al. 2008; Linnet and Teilum 2016), methods exploiting the knowledge-based confinement of frequency space (Kazimierczuk et al. 2010; Matsuki et al. 2011; Frey et al. 2013), variants of the Radon transform (Kupče and Freeman 2013; Dass et al. 2017; Rytel et al. 2019; Nawrocka et al. 2019; Romero et al. 2020; Shchukina et al. 2021), and others (Shchukina et al. 2017b).

Below, as an example of applying the method—and a challenging one at that—we examine the temperature dependence of membrane-protein interactions. This field of research often focuses on the transition temperature of the membrane. But the temperature dependence of protein-membrane interactions can also be drastically influenced by the variability of the bound state or states, as is the case in our system. $\alpha$-Synuclein (aSyn) is a small protein predominantly located in the presynaptic termini of neurons (Iwai et al. 1995). It has been widely studied due to its involvement in several diseases (multiple system atrophy, Parkinson’s disease, dementia with Lewy bodies) (Wakabayashi et al. 1998; Konno et al. 2016) that currently lack a disease-modifying treatment, and also due to its structural heterogeneity. In vivo aSyn is partitioned between the cytosol and cellular membranes, with the cytosolic state showing intrinsic disorder (Theillet et al. 2016). aSyn plays a physiological role in the synaptic vesicle cycle, where it has been observed to influence vesicle clustering and fusion (Das et al. 2022). It adopts radically different structures depending on whether it is membrane-bound, free in solution, or part of a fibril. In addition, even the membrane-bound state of the protein consists of an ensemble of states that have been shown to depend on the protein:membrane ratio and the specific membrane composition and buffer conditions (Viennet et al. 2018). Below, we show how changing the measurement temperature can shift the overall fraction of free and membrane-bound states, as well as the structural ensemble of the membrane-bound state. We also demonstrate that the data can be collected quickly and effectively processed with a new “difference CS” approach.

## Method

Below, we present the fundamentals of the proposed difference compressed sensing (DCS) method and the procedures we use to boost its effectiveness. We also discuss sample preparation and measurement conditions, as well as the curve-fitting procedure used in the data analysis.

### Concept and features of difference compressed sensing

**Fig. 3:**
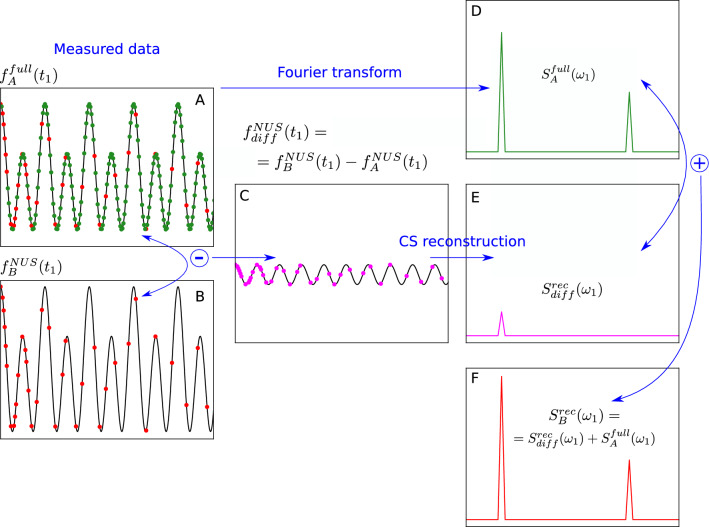
Difference compressed sensing (DCS). **A** Fully sampled time-domain signal. **B** Non-uniformly sampled time-domain signal *similar* to A. **C** Difference between experimentally undersampled time-domain signal B and artificially undersampled time-domain signal A. The similarity between A and B causes C to be more compressible than B. The artificial undersampling of A (red dots in panel A) is performed according to the experimental undersampling B. **D** Fully sampled spectrum of A. **E** CS-reconstructed difference spectrum. **F** Reconstructed difference spectrum added to full spectrum A. This final result corresponds to spectrum B; all the panels represent a column of a 2D spectrum/interferogram, i.e., fixed $\omega _2$

With two sufficiently similar spectra at our disposal, we can increase the sparsity by taking their difference (see Fig. 3). Let us consider two 2D time-domain signals, $f_A(t_1, t_2)$ and $f_B(t_1, t_2)$. In an experiment we acquire the full signal A, $f_A^{full}(t_1, t_2)$, and signal B undersampled in $t_1$, $f_B^{NUS}(t_1, t_2)$. The former is processed in the conventional manner, yielding the spectrum $S_A^{full}(\omega _1,\omega _2)$. We then apply the sampling schedule of signal B to signal A, that is to say, we artificially undersample signal A in the same way that we undersampled B. This gives us $f_A^{NUS}(t_1,t_2)$. Finally, we take the difference between the two undersampled FIDs: $f_{diff}^{NUS}(t_1,t_2) = f_B^{NUS}(t_1,t_2) - f_A^{NUS}(t_1,t_2)$

This then forms the input to the reconstruction algorithm. As spectra A and B are similar, some peaks will disappear in this difference, increasing the sparsity of the reconstruction input. Thus, the number *K* of significant points will be lower, so we will be able to safely decrease the number *M* of acquired points (see Eq. (1)). After the reconstruction, we need to add its output to spectrum A to obtain the reconstructed spectrum B: $S_B^{rec}(\omega _1,\omega _2)=S_{diff}^{rec}(\omega _1,\omega _2)+S_A^{full}(\omega _1,\omega _2)$.

Spectrum A does not necessarily have to be fully sampled: It can also be undersampled, as long as this is done at a reasonably high level. The sampling schedule of B should be a subset of the sampling schedule of A. In this case, the additional step of reconstructing A is required.

### Increasing sparsity by minimizing differences

**Fig. 4:**
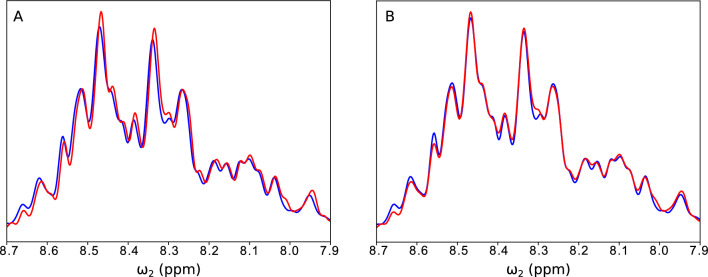
Minimizing differences using DCS pre-processing. **A** The superposition of the first $t_1$ increment of 2D ${{}^{1}\hbox {H}}-{{}^{15}\hbox {N}}$ HSQC of aSyn with liposomes (blue) and without liposomes (red), before correction. There are slight differences in the linewidth and peak positions. **B** Superposition of spectra from panel **A**, corrected to minimize the norm of the residual between them (as in Eq. (2)). The correction algorithm yields an optimal shift of two spectral points and optimal line broadening of 1 Hz

In the case of DCS we can considerably reduce the number of significant spectral points *K* by making the two spectra even more similar, for example, by suppressing the differences in peak positions, linewidths, and amplitudes. To some extent the latter can be done deterministically, for instance by considering the sample dilution upon the addition of liposomes. We can suppress differences in peak positions and linewidths very effectively in the direct dimension by comparing the 1D spectra of the first $t_1$ increment, $f_A(t_1=0, \omega _2)$ and $f_B(t_1=0, \omega _2)$. Slightly different experimental conditions affect peak positions, amplitudes, and linewidths. As a result, $f_A(t_1=0, \omega _2)$ and $f_B(t_1=0, \omega _2)$ differ as shown in Fig. 4A.

In our example we applied a simple brute-force algorithm that finds the correction of peak positions and linewidths by minimizing the norm of the residual between $f_A(t_1=0, \omega _2)$ and $f_B(t_1=0, \omega _2)$:

2 $$\begin{aligned}{} & {} \mathop {\mathrm {arg\,min}}\limits _{\sigma , \Delta \omega _2} || f_A^{full}(t_1=0, \omega _2-\Delta \omega _2) *\exp (-\frac{\omega _2^2}{2 \sigma ^2}) - f_B^{NUS}(t_1=0, \omega _2)|| \end{aligned}$$

The correction for this example is shown in Fig. 4B. We use the values $\sigma$ and $\Delta \omega _2$, minimizing the difference of Eq. (2), to generate the sparsest possible “difference signal”, which we then use as input for the DCS reconstruction.

### Sample preparation and NMR experiments

The aSyn protein sample was uniformly labeled with ${{}^{15}\hbox {N}}$. The production and purification protocols are described elsewhere (Wrasidlo et al. 2016): In short, *E. coli* cells containing IPTG-inducable aSyn were grown to a high cell mass in LB medium, washed, and then transferred to ${{}^{15}\hbox {N}}$ ammonium chloride-supplemented M9 medium for induction. Expression was carried out for 16 h (h) at 28 °C, before the cells were harvested by centrifugation. After sonicating the resulting pellet, we carried out purification using heat precipitation, followed by streptomycin, ammonium sulfate and ethanol precipitations. Finally, we used an ion exchange chromatography step to obtain samples with $\ge 95\%$ purity as judged by SDS-PAGE analysis. We obtained POPG (1-palmitoyl-2-oleoyl-sn-glycero-3-phospho-(1′-rac-glycerol)) from Avanti Polar Lipids for the production of liposomes via extrusion through a 0.4 μM filter.

All NMR experiments were performed using a Varian 700 MHz DDR2 spectrometer with an HCN probe. Two NMR samples were used. The first was 0.13 mM ${{}^{15}\hbox {N}}$ labeled aSyn dissolved in 585 μL of an ${\hbox {H}_{2}\hbox {O}}:{\hbox {D}_{2}\hbox {O}}$ mixture (9:1,v/v) containing 50 mM of phosphate buffer (pH 6.5), 1 mM of EDTA, and 1 mM of sodium azide. The second sample was obtained by adding 65 μL of POPG liposomes (4 mg/mL dissolved in an identical buffer) to the first sample. For both NMR samples we measured a set of 15 ${{}^{1}\hbox {H}}-{{}^{15}\hbox {N}}$ HSQC spectra, spanning a temperature range of 15–43 °C (2 °C between consecutive spectra). Each HSQC spectrum was measured with a relaxation delay of 1.5 s (s), four scans, spectral widths of 11.468 kHz (${{}^{1}\hbox {H}}$) and 2.7 kHz (${{}^{15}\hbox {N}}$), and maximum evolution times set to 89 ms (${{}^{1}\hbox {H}}$) and 95 ms (${{}^{15}\hbox {N}}$), corresponding to 256 points measured in the indirect dimension. The assignment of aSyn signals was taken from previous work (Wrasidlo et al. 2016).

The spectra were artificially undersampled at various sampling levels (8, 16, 24, 32, 40, 48, 56, 64, and 128 points out of 256) with Poisson-gap samplings (Hyberts et al. 2010; Kazimierczuk et al. 2008; Kasprzak et al. 2021). For the reconstruction we used the iterative soft thresholding algorithm implemented in the mddnmr program (Orekhov et al. 2021), with default settings (200 iterations and virtual echo).

### Fitting peak intensity curves

As we expect the dependencies of peak relative intensities on temperature to have a sigmoidal shape, we approximated the experimental data with a hyperbolic tangent function (Fig. 2). We introduced parameters to scale the “basic” curve and shift it along both vertical and horizontal axes: $x_{scale}$, $x_{shift}$, $y_{scale}$, $y_{shift}$. Our approximation function thus has the following form:

3 $$\begin{aligned} y = y_{scale}\Bigl (1-\tanh \bigl (x_{scale}\cdot (x-x_{shift})\bigl ) \Bigl )/2+y_{shift} \end{aligned}$$

In the fitting procedure, *x* corresponds to the temperature of the experiment and *y* corresponds to peak relative intensity. Among the four adjustable parameters, $x_{shift}$ is of special interest as it defines the transition temperature, which is our primary concern. We apply the following boundaries to the parameters: $y_{scale}$, in between half the *y* range and two times it; $y_{shift}$, within the range of $\min (y) \pm 0.1$; and $0.05< x_{scale} < 0.8$; $20< x_{shift} < 40$ (transition temperatures between 20 and 40 °C).

**Fig. 5:**
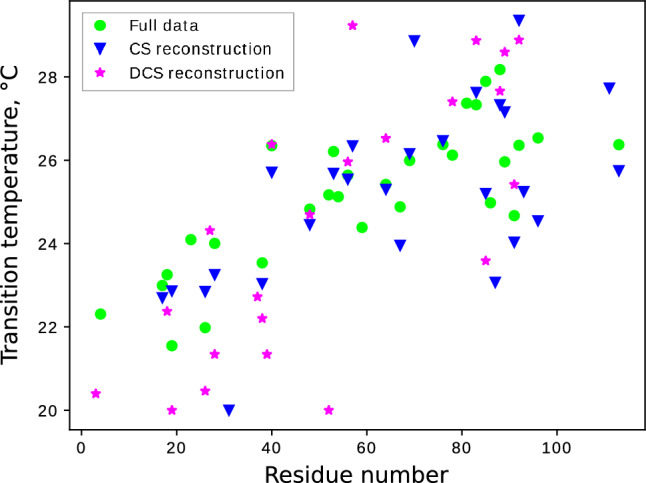
Transition temperature vs. residue number for the aSyn bound to liposomes. The values were determined by fitting sigmoidal curves, as shown in Fig. 2. Results of FT (full data), CS and DCS (64 NUS points) are marked with circle, Down-Pointing Triangle and star, respectively

We use the “curve_fit” optimization function from the SciPy Python library (Virtanen et al. 2020) with the Trust Region Reflective (‘trf’) algorithm (Moré and Sorensen 1983). The experimental profiles (full data) and their approximations for all the peaks visible in spectra, with and without POPG, are given in the SI. The transition temperatures of selected residues are given in Fig. 5. We omitted those with too few data points (fewer than six), where peaks went below the noise level too soon and were not visible in higher temperatures. We also filtered out peaks whose profiles were “not sigmoidal enough”. This applied in three different cases: First, some profiles were linear, as we could also see from the horizontal scaling parameter— if it was too little, it meant that the approximation yielded only the close-to-linear middle region of the sigmoid function and we could introduce the condition $x_{scale}>0.08$. Second, some profiles did manifest a transition but this transition was too small. If the last-but-one data point on both sides differed less than twice, we omitted those peaks. Finally, we excluded peaks whose approximation had too high a relative residual (more than 0.1). The rest, which give a reliable picture of a transition temperature, are shown in Fig. 5. As can be seen, the NUS reconstructions - CS and DCS - reveal a trend in transition temperatures similar to that seen in the FT of the full data.

## Results and Discussion

We applied DCS and conventional CS to a series of 2D ${{}^{1}\hbox {H}}-{{}^{15}\hbox {N}}$ HSQC spectra of aSyn acquired at various temperatures, with and without the addition of liposomes. The results are interesting in terms of both biophysics and signal processing. We discuss both contexts below.

### aSyn-liposome interactions

**Fig. 6:**
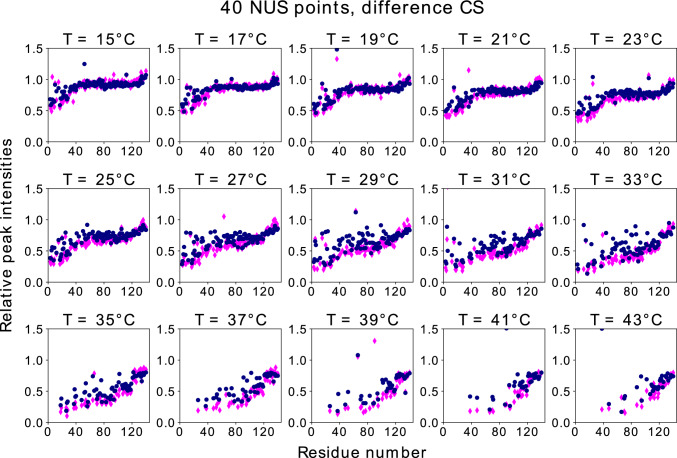
DCS reconstruction results: relative heights of peaks in ${{}^1\hbox {H}}-{{}^{15}\hbox {N}}$ HSQC of aSyn with and without liposomes versus residue number. Various temperatures, NUS level of 40 points and full data results. Normalized residuals are as follows (from lowest temperature to highest): 0.081; 0.068; 0.064; 0.108; 0.100; 0.128; 0.195; 0.320; 0.307; 0.290; 0.267; 0.256; 0.230; 0.442; 0.510

**Fig. 7:**
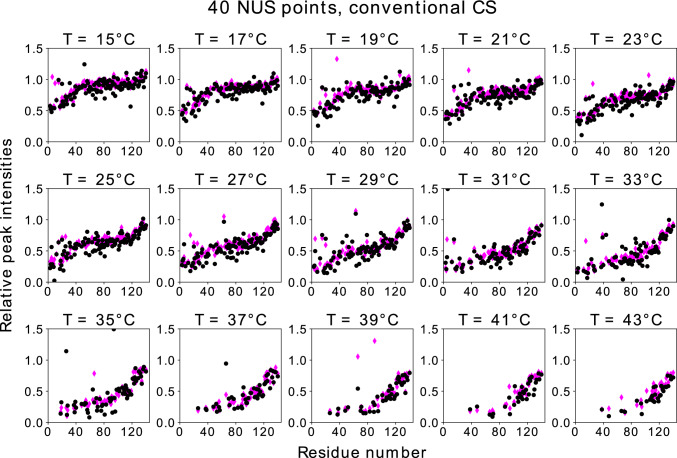
Conventional CS reconstruction results: relative heights of peaks in ${{}^{1}\hbox {H}}-{{}^{15}\hbox {N}}$ HSQC of aSyn with and without liposomes vs. residue number. Various temperatures, NUS level of 40 points and full data results. Normalized residuals are as follows (from lowest temperature to highest): 0.125; 0.093; 0.141; 0.113; 0.135; 0.138; 0.169; 0.121; 0.154; 0.260; 0.403; 0.178; 0.330; 0.459; 0.172

The high internal dynamics of aSyn in its intrinsically disordered regions, combined with the high molecular mass of the liposomes used in this study and a slow exchange rate between free and bound states (Bodner et al. 2009), provide a remarkable way of indirectly obtaining information on the bound state of the protein. When aSyn is bound to a liposome, all stably bound regions of the protein have similar relaxation behavior to the liposome itself and are thus not directly detectable by NMR. However, the unbound regions of aSyn retain such high levels of flexibility that the signal intensities obtained are nearly unchanged compared to the unbound state of the protein. We can therefore obtain the fraction of stably bound aSyn by measuring the peak intensity of each residue with liposomes relative to its unbound state ($I_{rel}$). We then use the residue-resolved information obtained by this method to get information on various binding states as shown in (Viennet et al. 2018), who relied on  the approach after it was verified by studying the bound state indirectly using CEST methodology (Fusco et al. 2014). Tracking of relative peak intensities along the primary sequence of the protein has been used to study the effect of different membrane compositions (Viennet et al. 2018), as well as to obtain information on the binding mode and efficacy of pharmaceutical compounds (Wrasidlo et al. 2016). Here, we used this approach to study the temperature dependence of aSyn-liposome interactions. The changes taking place as the measurement temperatures increase are readily observed when comparing the peak intensity pattern along the primary sequence at different temperatures (see Figs. 6 and 7 ).

At low temperatures (15 °C), binding is nearly entirely restricted to the 25 most N-terminal residues of aSyn. But higher temperatures (35 °C) show strong binding for more than 100 residues, leaving only the C-terminal residues relatively unaffected. While the higher temperatures lead us to expect faster exchange between free and bound forms, the very slow exchange described (Bodner et al. 2009) for intermediate temperatures and also the lack of residue-specific peak shifts induced by liposomes, which would be expected for fast exchanging species, allow us to interpret the observed curves as a reflection of the bound fraction of protein regions. The dense sampling of temperatures in this study provides further insights into the intriguing changes observed in aSyn-liposomes interactions.

Tracking the relative intensities on a per-residue basis across the measured temperature range, we see that not only is there a reduction in peak intensity, but for many residues this transition is non-linear. By fitting a sigmoidal function to these residues, we can obtain the temperature of maximal change (that is, the “melting” or “transition temperature”; see Fig. 5). For peaks displaying non-linear behavior, the temperature of transition from one binding mode (or ensemble of structures) to another rises along the primary sequence of the protein. Although the temperature range is narrow, we see that the N-terminal region (AAs 1–40) of the protein switches to a larger bound fraction earlier than the remainder of its membrane binding region. It has previously been observed that aSyn’s N-terminus has a higher membrane affinity (Bartels et al. 2010). However, as far as we are aware, the temperature dependence of binding for its latter part has not been studied. That said, the disconnect between the regions is well established: Early work has demonstrated a helical break around residue 45 for micelle-bound aSyn (Chandra et al. 2003; Ulmer et al. 2005; Bussell 2005), and this has also been shown to exist in the more physiological conditions of vesicle-bound aSyn (Lokappa and Ulmer 2011). The transition takes place at around 26 °C, close to standard measurement temperatures of 25 °C and above most room-temperature experiments, underscoring the importance of considering the possible effects of the measurement temperature on experimental observables.

The protein:liposome ratio is constant over the entire temperature range, so the larger amount of bound protein regions at higher temperatures reflects either a structural change in the membrane or a structural change in the bound state of aSyn. As the transition temperature is far removed from the melting temperature of POPG-based bilayers (Wiedmann et al. 1993), a temperature-dependent structural change in the membrane would be a local effect, driven by the prior structural ordering of lipids through aSyn, as is observed for other proteins (Drücker et al. 2013; Varyukhina et al. 2022). Membrane curvature modification by aSyn has been suggested (Braun et al. 2012), so a localized influence on the order of lipid molecules is likely. Therefore, additional binding of aSyn could be facilitated by a further insertion in the lipid bilayer or by interactions between bound aSyn entities in its new structure, or both as indicated in (Schwarz et al. 2023).

The data presented shows a change in the bound state of aSyn at elevated temperatures. This change is not a simple transition to one new bound state, but rather involves intermediate states or more than one new bound state: The transition is not uniform throughout the entire length of the protein. We are thus possibly observing a synergistic process involving both an alteration of the lipid structural order and aSyn structural re-arrangement. It is interesting to note that the two major regions with differing temperature-dependent binding profiles fit in well with the known helical break within the structure of aSyn - which, it has been proposed, plays a central role in its physiological function. On the one hand, the interaction of the C-terminal helix improves the odds of forming a membrane anchor at the N-terminus (Cholak et al. 2020); on the other, the two helices are subsequently also capable of binding to two separate membranes, enabling membrane fusion processes (Das et al. 2022). The strong impact that different lipid compositions have on the binding affinity and binding mode of aSyn (Man et al. 2021) is likely mirrored in the temperature dependence that we observe.

### NUS reconstruction—DCS versus CS

For each temperature in a series, we performed DCS reconstruction for spectra with the addition of liposomes ($S_B(\omega _1,\omega _2)$), using fully sampled spectra without liposomes ($S_A(\omega _1,\omega _2)$) as a basis. As all the data was acquired using full sampling, we could test various NUS levels by applying artificial undersampling. The indicator of aSyn-liposome binding, $I_{rel}$, served as a quality factor. In our analysis we sought to answer the following questions: What are the advantages and disadvantages of DCS in terms of reconstruction quality and sensitivity? How does conventional CS compare with DCS for spectra with different signal-to-noise ratios and NUS levels? Can we use NUS methods other than CS/IST to process difference signals? Is DCS applicable to serial measurements other than VT-NMR? And does it make sense to apply it to dimensionalities three and more?

**Fig. 8:**
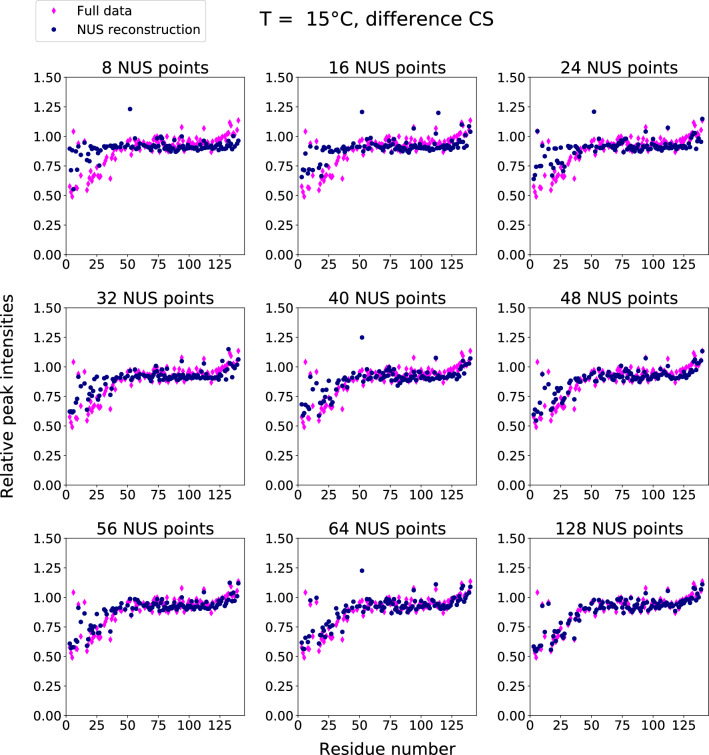
DCS reconstruction results: relative heights of peaks in ${{}^{1}\hbox {H}}-{{}^{15}\hbox {N}}$ HSQC of aSyn with and without liposomes versus residue number. Various NUS sampling levels and full data results. T=15 C$^{\circ }$ (see Supplementary Information for results at higher temperatures). Normalized residuals are as follows (from lowest sampling level to highest): 0.136; 0.105; 0.091; 0.087; 0.081; 0.071; 0.070; 0.067; 0.055

**Fig. 9:**
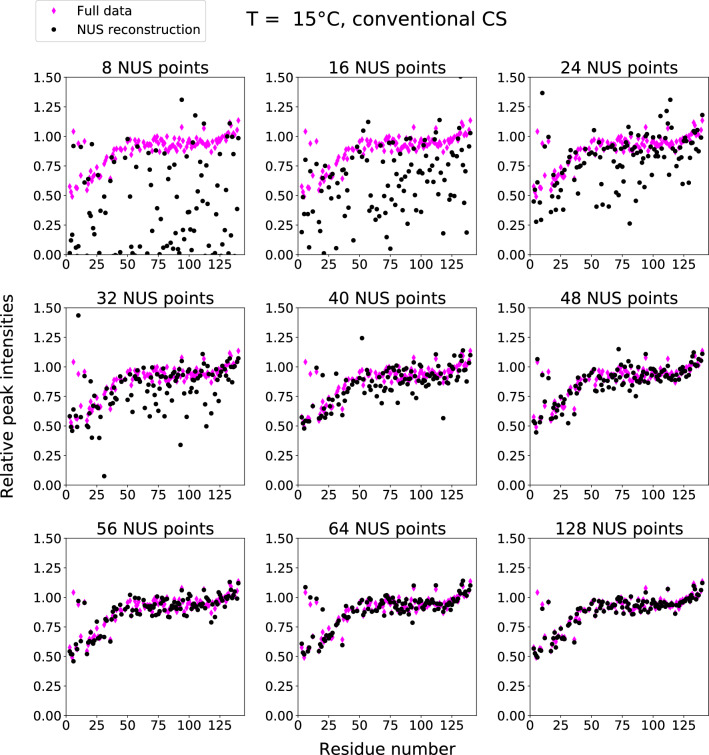
Conventional CS reconstruction results: relative heights of peaks in ${{}^{1}\hbox {H}}-{{}^{15}\hbox {N}}$ HSQC of aSyn with and without liposomes versus residue number. Various NUS sampling levels and full data results. T=15 C$^{\circ }$ (see Supplementary Information for results at higher temperatures). Normalized residuals are as follows (from lowest sampling level to highest): 0.721; 0.463; 0.220; 0.183; 0.125; 0.060; 0.075; 0.041; 0.060

Figure 8 shows $I_{rel}$ in DCS and fully sampled spectra as a function of the residue number for the lowest temperature in the series (15 °C). It is worth comparing these plots with those presented in Fig. 9. For lower sampling levels (32 or 40 NUS points), the DCS reconstruction is much better than the CS reconstruction. It provides a very good $I_{rel}$ profile and unambiguously indicates that residues close to N-terminus are involved in binding. Thus, the increased sparsity provided by DCS does indeed lead to better reconstruction. At higher sampling levels, the CS reconstruction is better than DCS as taking the difference amplifies the noise by approximately $\sqrt{2}$. On the other hand, if there are enough NUS points, condition Eq. (1) is fulfilled. At higher temperatures (over 23 °C), the noise amplification problem becomes so pronounced that DCS cannot be considered superior to conventional CS. This fact also manifests itself in the residuals of the peak heights of the reconstructed data compared to those of the full data. For each panel in Figs. 6, 7, 8, and 9, we provide the corresponding normalized residuals: the l2-norm of the difference of the reconstructed and the full height vectors, divided by the l2-norm of the full height vector. In the Supplementary Information, we also provide a list of all the normalized residuals for all temperatures and sampling levels.

To sum up, using only 64 points from a 256 points grid allows us to reconstruct the temperature curves with high fidelity, providing melting temperatures (see Fig. 5). At an even lower NUS level (40 points) the resulting trends (Fig. 6) are satisfactory in qualitative terms. We can now collect the data for the samples with liposomes in approximately two to three hours, compared to 12 h for full sampling.

**Fig. 10:**
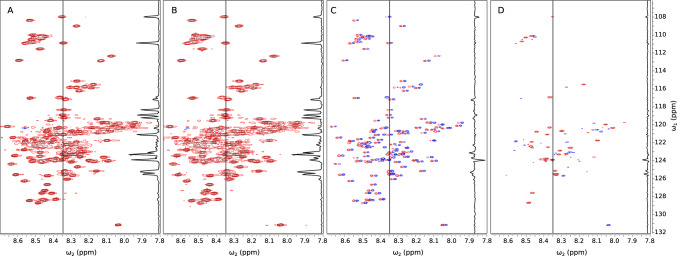
2D ${{}^{1}\hbox {H}}-{{}^{15}\hbox {N}}$ HSQC of aSyn at T=15$^{\circ }$C and an example vertical trace (8.347 ppm in $\omega _2$). **A** Spectrum of aSyn sample without liposomes. **B** Spectrum of aSyn sample with liposomes. **C** Raw difference between A and B. **D** Difference spectrum optimized according to Eq. (2)

The peculiar shape of a “difference spectrum” (Fig. 10C) should be taken into account when choosing an appropriate NUS method for its reconstruction. Some of the algorithms, such as SMILE (Ying et al. 2017), SCRUB (Coggins et al. 2012), and LPMP (Kazimierczuk and Kasprzak 2014) assume a certain lineshape, typically a mixture of Gaussian and Lorentzian. Others, such as R-MDD (Jaravine et al. 2006) or low-rank completion (Qu et al. 2015) are not so strict, but work most effectively when the signal is auto-recursive (for example, exponential). Such assumptions are not necessarily fulfilled in difference spectra. The reconstruction algorithm in this case should not therefore assume any particular peak shape. Iterative soft thresholding (Kazimierczuk and Orekhov 2011; Hyberts et al. 2012; Sun et al. 2015), iteratively re-weighted least squares (Kazimierczuk and Orekhov 2011; Kazimierczuk et al. 2012), maximum entropy (Hoch 1969; Mobli and Hoch 2008), and machine-learning methods (Hansen 2019; Karunanithy and Hansen 2020; Luo et al. 2020; Jahangiri et al. 2023) employing dedicated networks are all suitable for difference spectra. Methods based on the assumption of dark spectral regions, such as SIFT (Matsuki et al. 2011) or iterated maps (Frey et al. 2013), could also work effectively on the difference signals. Moreover, the bright regions can be severely narrowed down. Notably, the virtual echo procedure (Mayzel et al. 2014) is fully applicable to DCS—and indeed was effectively used in this study.

Is it possible to co-process the data with and without liposomes using multidimensional decomposition (Orekhov and Jaravine 2011)? Indeed, if the differences between spectra $S_A(\omega _1,\omega _2)$ and $S_B(\omega _1,\omega _2)$ were only a matter of amplitudes (peak heights), then individual peak shapes in $S_B(\omega _1,\omega _2)$ could be effectively determined from $S_A(\omega _1,\omega _2)$ by multidimensional decomposition. The NUS reconstruction of $S_B(\omega _1,\omega _2)$ would then be limited to determining peak heights. Unfortunately, the slight shifts of resonance frequencies between spectra make this approach unfeasible.

Another option we considered and found ineffective in determining $I_{rel}$ is a quasi-Radon transform of a non-stationary signal, as discussed in Shchukina et al. (2021). This method is good at determining the changes in resonance frequency, but not so good for peak height variations. Although these variations are encoded in the lineshapes (see Fig. 1 in Dass et al. (2017)), they are not easy to decode using currently available methods.

Importantly, DCS could be the optimal approach for speeding up other serial NMR experiments in which low spectral sparsity limits the use of conventional CS. If the signal-to-noise ratio is reasonably high, one might consider using it for reaction monitoring, for example. By contrast, if most peaks in the spectrum change their intensities—for example, in 3D DOSY and 2D relaxation series—taking the difference does not improve sparsity and the use of DCS is not justified.

As with conventional CS, a high dynamic range of signal intensities in the spectrum is challenging for DCS (Wieske and Erdélyi 2021). Sometimes, however, using a difference signal may lower the intensity range. When studying reactions occurring in mixtures, for instance, consecutive spectra in the series of measurements may only show changes in small components, thus improving the CS reconstruction conditions not only in terms of sparsity, but also dynamic range.

For serial measurements of dimensionality higher than two, the low sensitivity is more problematic than the mathematical conditions of NUS reconstruction (Eq. (1)). As shown elsewhere (Kazimierczuk et al. 2014), it often makes sense to worsen the latter and improve the signal-to-noise ratio, for example, using the relaxation-matched NUS density (Barna et al. 1987). For dimensionalities higher than three, thousands of NUS points are usually collected for sensitivity reasons, making even conventional CS reconstruction unnecessary (Kazimierczuk et al. 2009, 2013). Thus, in our opinion, the main field of application for DCS is likely serial 2D NMR with high sensitivity and low sparsity, combined with high sparsity of the “difference spectrum”. The variable pressure or variable temperature series are good examples of this.

## Conclusion

The efficacy of NUS/CS reconstruction depends on the sparsity (compressibility) of the spectrum. We can sometimes improve the quality of the reconstruction by focusing only on the differences between NUS data and similar spectra that are known in advance. In the case of aSyn, as described in this paper, the DCS approach allows us to determine temperature-dependent transitions in the bound ensemble with much faster signal acquisition times than is the case with conventional CS. The information obtained on the temperature dependence of aSyn binding shows two somewhat independent regions, which fit well with published information on its membrane-bound structure and the role this structure plays in its physiological function. This new information on temperature dependence should be taken into consideration in future investigations of this protein.

Importantly, after acquiring the signal it is possible to choose between CS and DCS. In so doing, one should consider the strengths and weaknesses of each approach. Thus, while DCS increases sparsity and improves the mathematical conditions of the reconstruction, it worsens the signal-to-noise ratio. Conventional CS, by contrast, is more sensitive. As we show in this paper, CS and DCS are complementary tools. Indeed, in some cases, the optimal approach may be to use both approaches in a single study.

## Supplementary Information

Below is the link to the electronic supplementary material.

Supplementary material 1 (PDF 9088.5 kb)—The Supplementary Information contains figures analogous to Fig. 2 for all the residues, and figures analogous to Figs. 8 and 9 for all the temperatures. The normalized differences between results from fully sampled and reconstructed data are also shown.

## Data Availability

The datasets and processing scripts are available at Zenodo (10.5281/zenodo.7715704).
