# Supplementary material for: Non-uniform sampling of similar NMR spectra and its application to studies of the interaction between alpha-synuclein and liposomes
Source: J Biomol NMR. 2023 May 26;77(4):149–63. doi: 10.1007/s10858-023-00418-3 (PMC10406685; doi:10.1007/s10858-023-00418-3)
Supplement: Supplementary file 1 — Supplementary material 1 (PDF 9088.5 kb)—The Supplementary Information contains figures analogous to Fig. 2 for all the residues, and figures analogous to Figs. 8 and 9 for all the temperatures. The normalized differences between results from fully sampled and reconstructed data are also shown. [file 10858_2023_418_MOESM1_ESM.pdf]

Supplementary information

"Non-uniform sampling of similar NMR spectra and its application to studies of the interaction between alpha-synuclein and liposomes"

Alexandra Shchukina[1], Thomas C. Schwarz[2], Michal Nowakowski[1], Robert Konrat[2], and Krzysztof Kazimierczuk[* 3]

[1]Faculty of Chemistry, University of Warsaw, Pasteura 1, 02-093 Warsaw, Poland

[2]Department of Structural and Computational Biology, University of Vienna, Max Perutz Labs, Vienna BioCenter Campus 5, 1030 Vienna, Austria

[3]Centre of New Technologies, University of Warsaw, Banacha 2C, 02-097 Warsaw, Poland, k.kazimierczuk@cent.uw.edu.pl

Figure S1: CS and DCS reconstruction results: relative heights of peaks in 1H-15N HSQC of aSyn with and without liposomes vs. residue number.

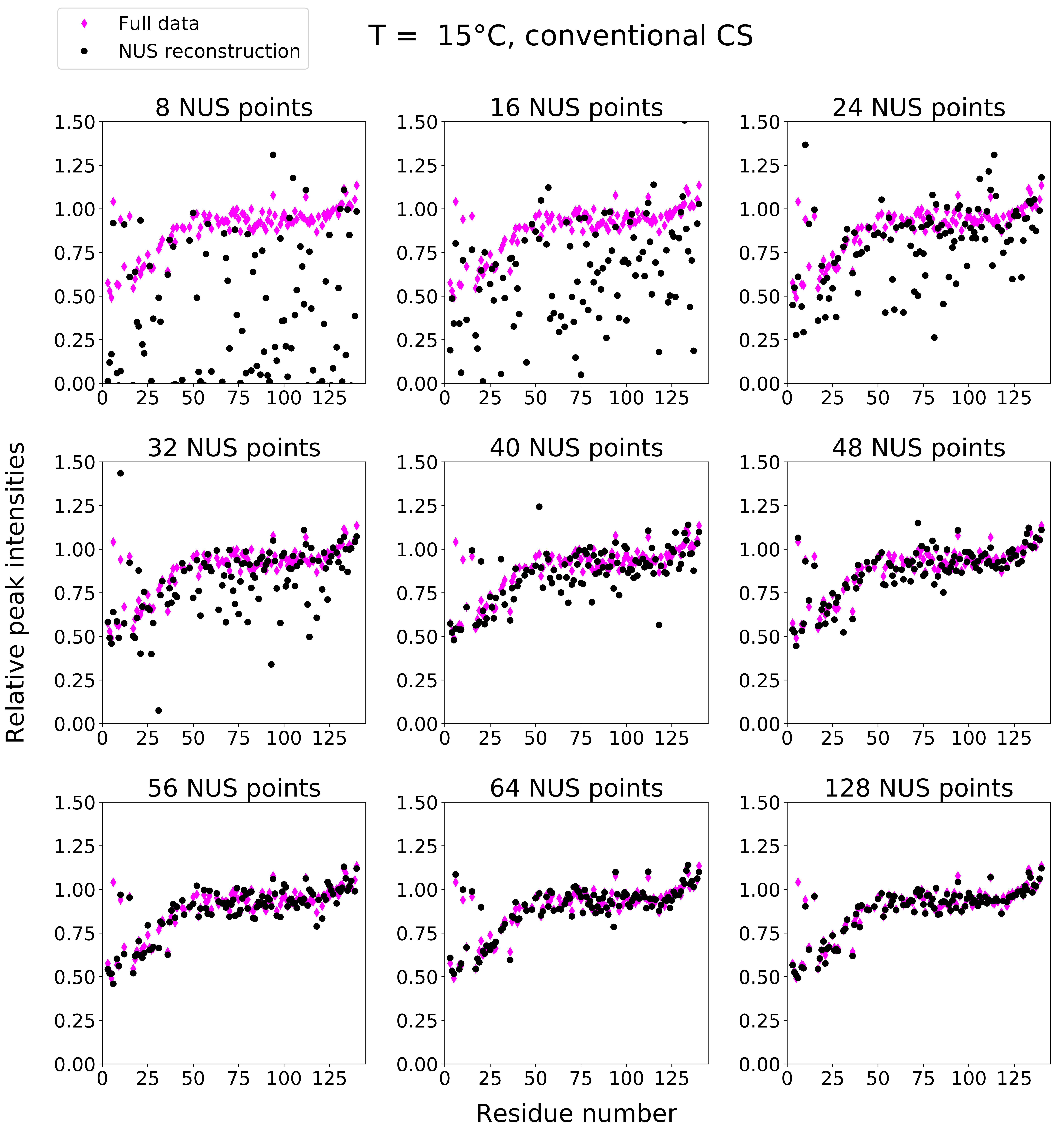

Full data
NUS reconstruction
T = 15°C, conventional CS
8 NUS points
16 NUS points
24 NUS points
32 NUS points
40 NUS points
48 NUS points
56 NUS points
64 NUS points
128 NUS points
Relative peak intensities
Residue number

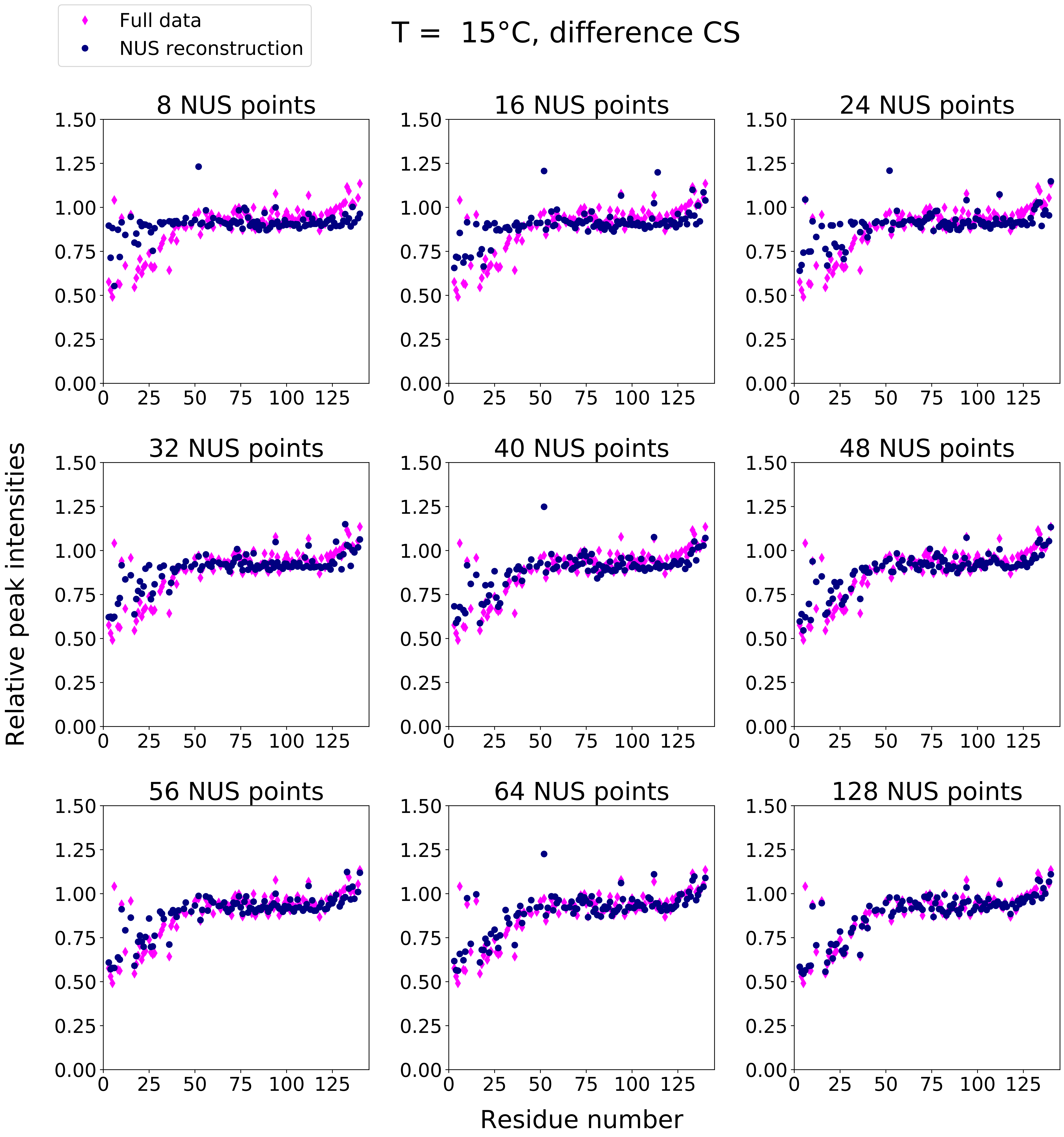

Full data
NUS reconstruction
T = 15°C, difference CS
8 NUS points
16 NUS points
24 NUS points
32 NUS points
40 NUS points
48 NUS points
56 NUS points
64 NUS points
128 NUS points
Relative peak intensities
Residue number

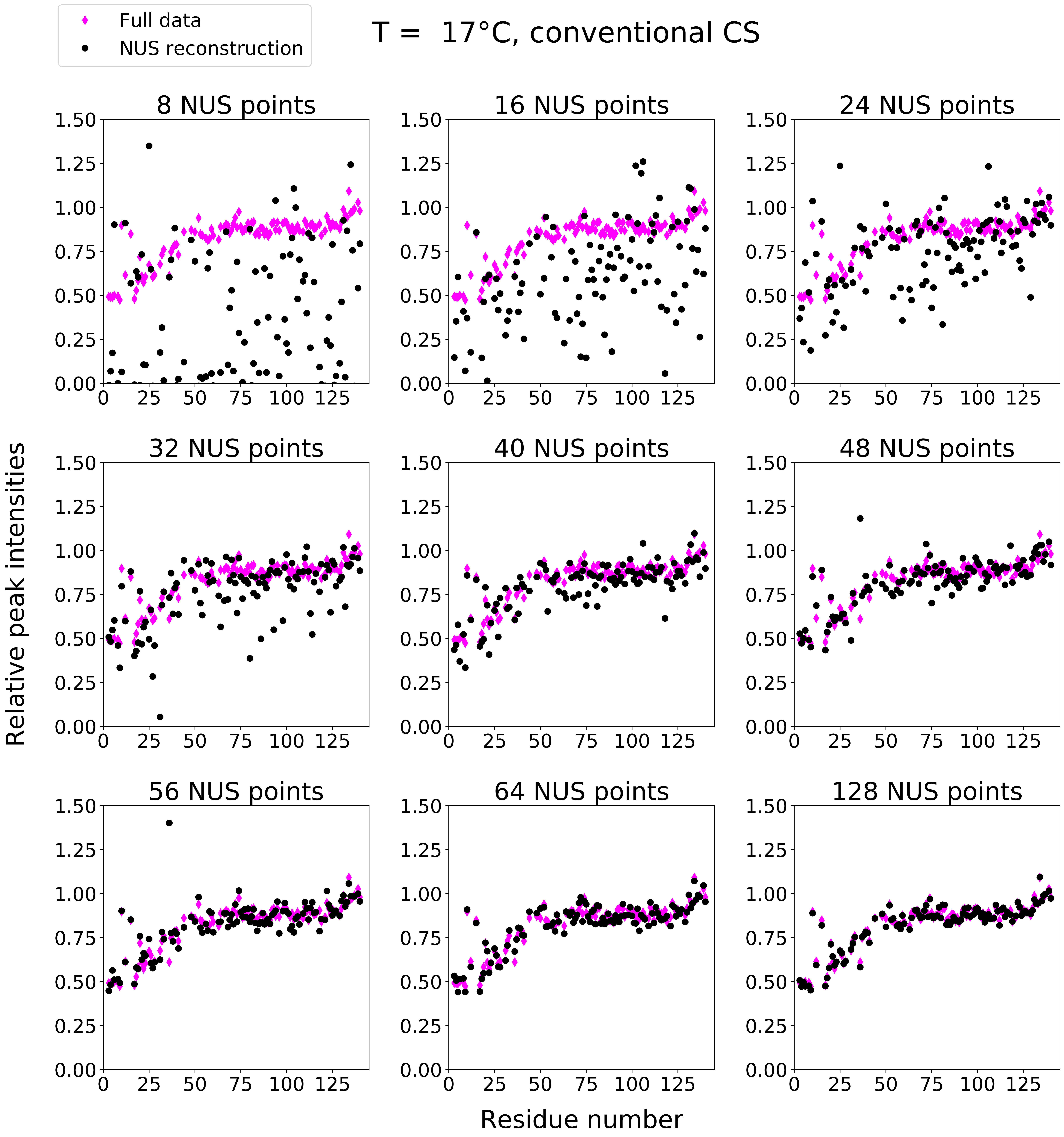
Full data
NUS reconstruction
T = 17°C, conventional CS
8 NUS points
16 NUS points
24 NUS points
32 NUS points
40 NUS points
48 NUS points
56 NUS points
64 NUS points
128 NUS points
Relative peak intensities
Residue number

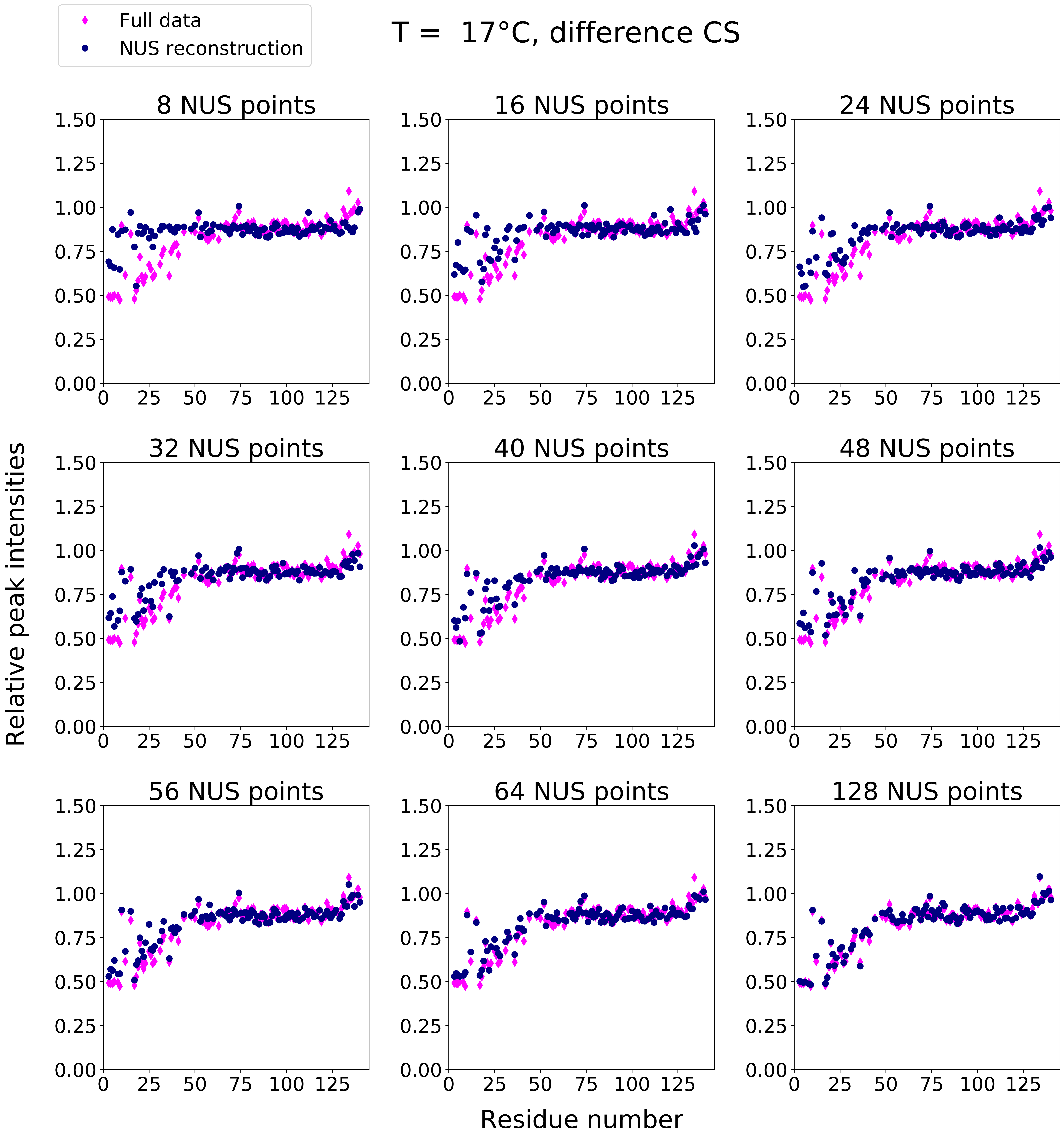

Full data
NUS reconstruction
T = 17°C, difference CS
8 NUS points
16 NUS points
24 NUS points
32 NUS points
40 NUS points
48 NUS points
56 NUS points
64 NUS points
128 NUS points
Relative peak intensities
Residue number

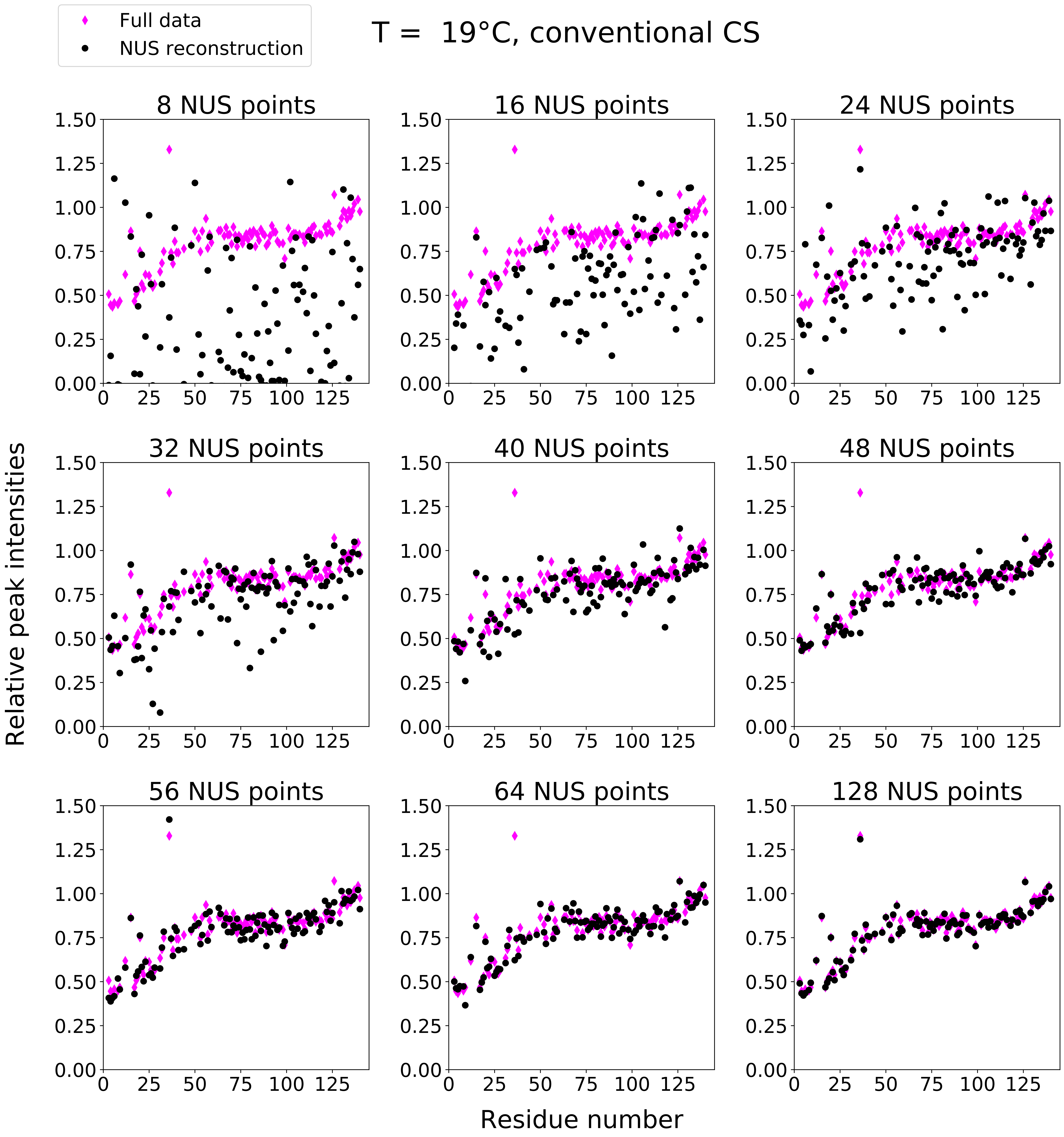

Full data
NUS reconstruction
T = 19°C, conventional CS
8 NUS points
16 NUS points
24 NUS points
32 NUS points
40 NUS points
48 NUS points
56 NUS points
64 NUS points
128 NUS points
Relative peak intensities
Residue number

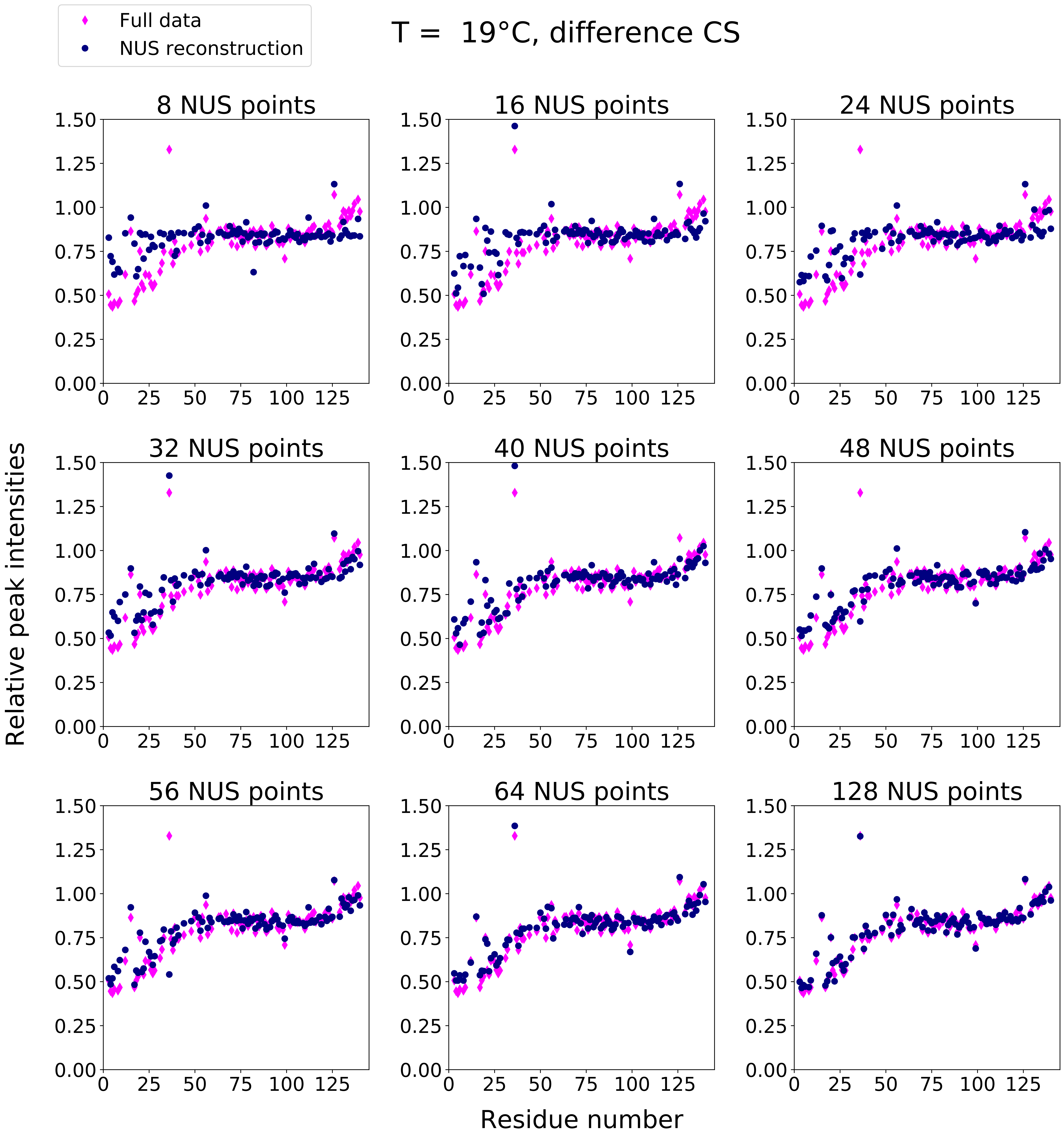

Full data
NUS reconstruction
T = 19°C, difference CS
8 NUS points
16 NUS points
24 NUS points
32 NUS points
40 NUS points
48 NUS points
56 NUS points
64 NUS points
128 NUS points
Relative peak intensities
Residue number
0.00
0.25
0.50
0.75
1.00
1.25
1.50
0
25
50
75
100
125

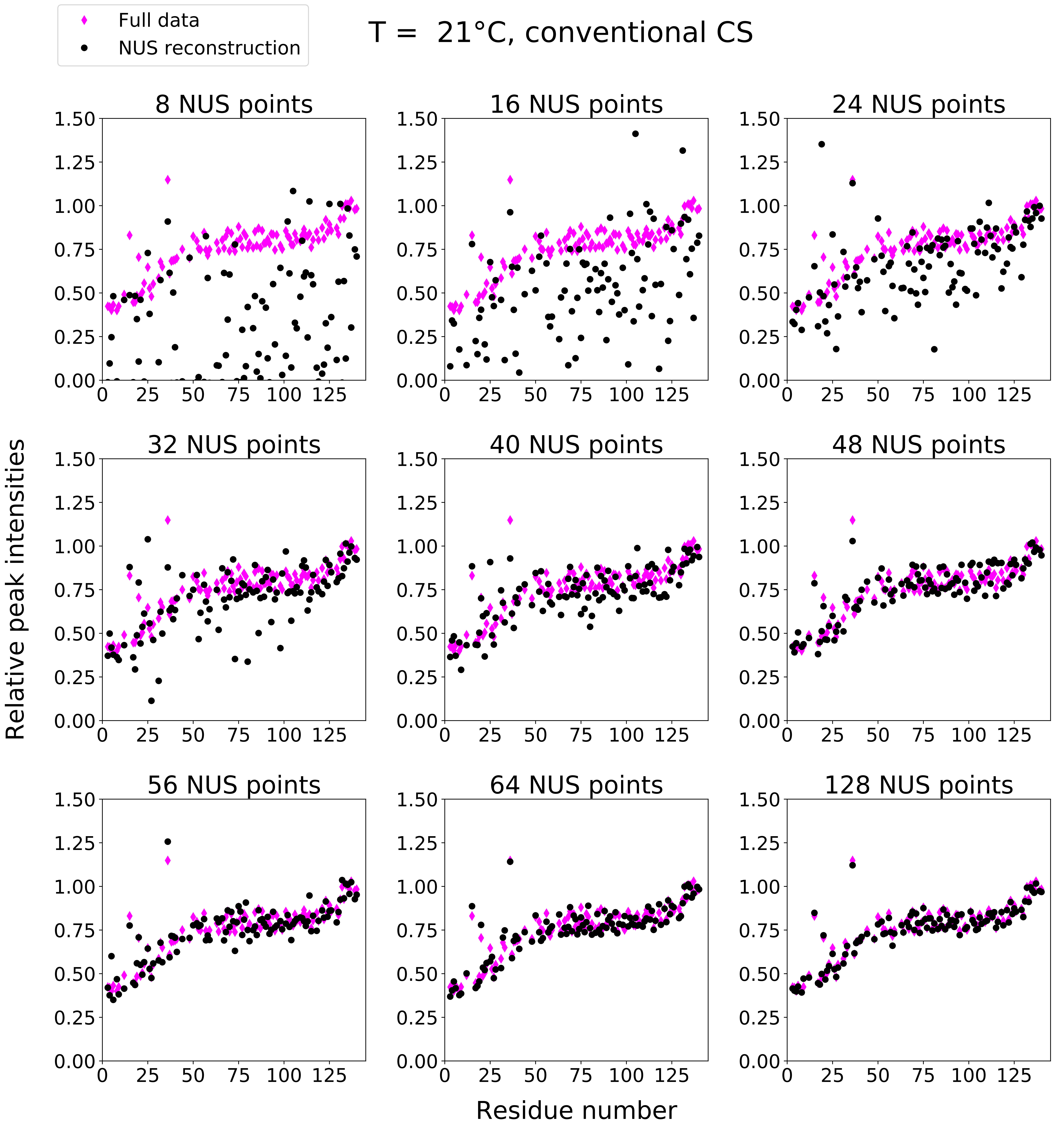

Full data
NUS reconstruction
T = 21°C, conventional CS
8 NUS points
16 NUS points
24 NUS points
32 NUS points
40 NUS points
48 NUS points
56 NUS points
64 NUS points
128 NUS points
Relative peak intensities
Residue number

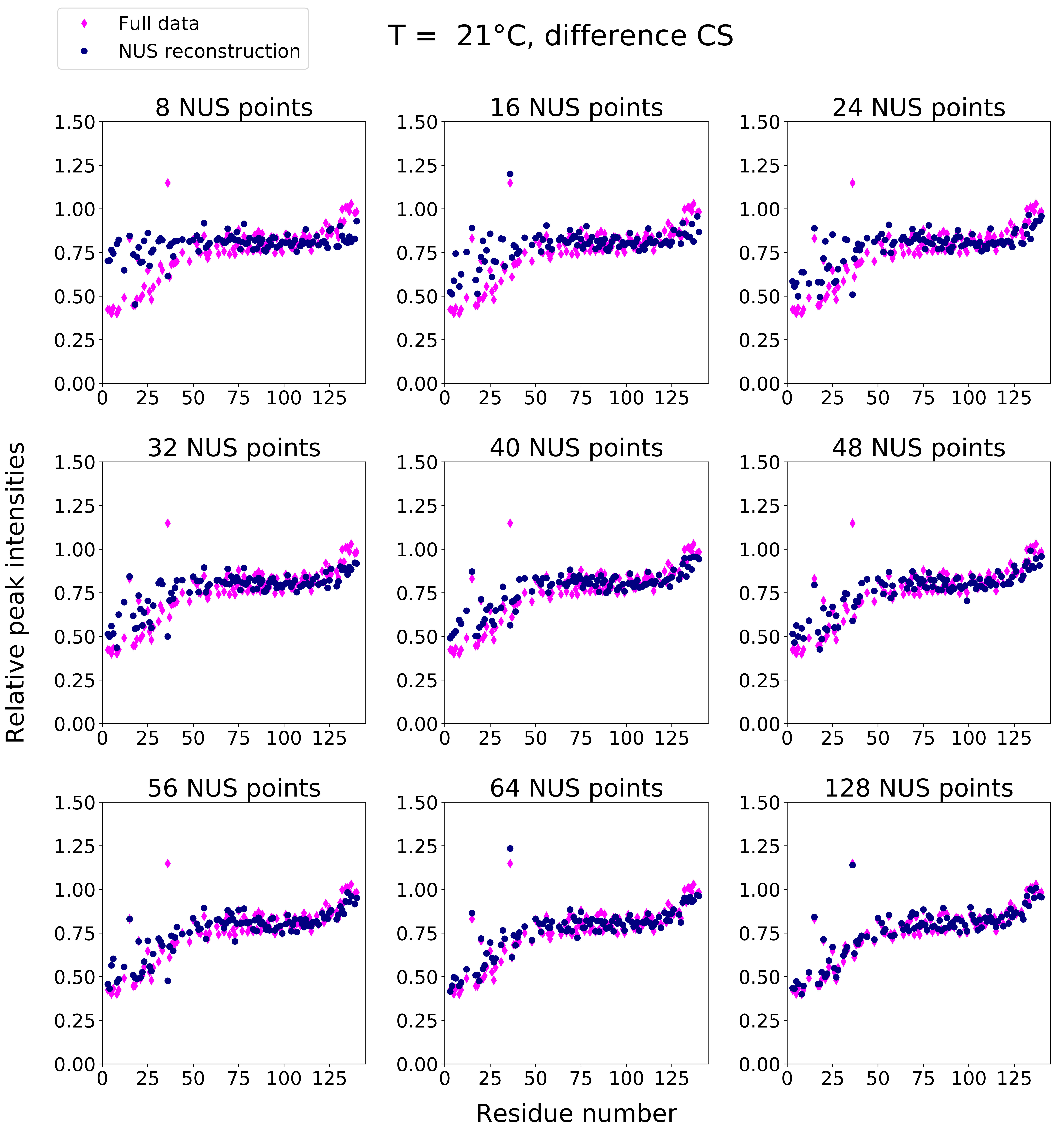

Full data
NUS reconstruction
T = 21°C, difference CS
8 NUS points
16 NUS points
24 NUS points
32 NUS points
40 NUS points
48 NUS points
56 NUS points
64 NUS points
128 NUS points
Relative peak intensities
Residue number

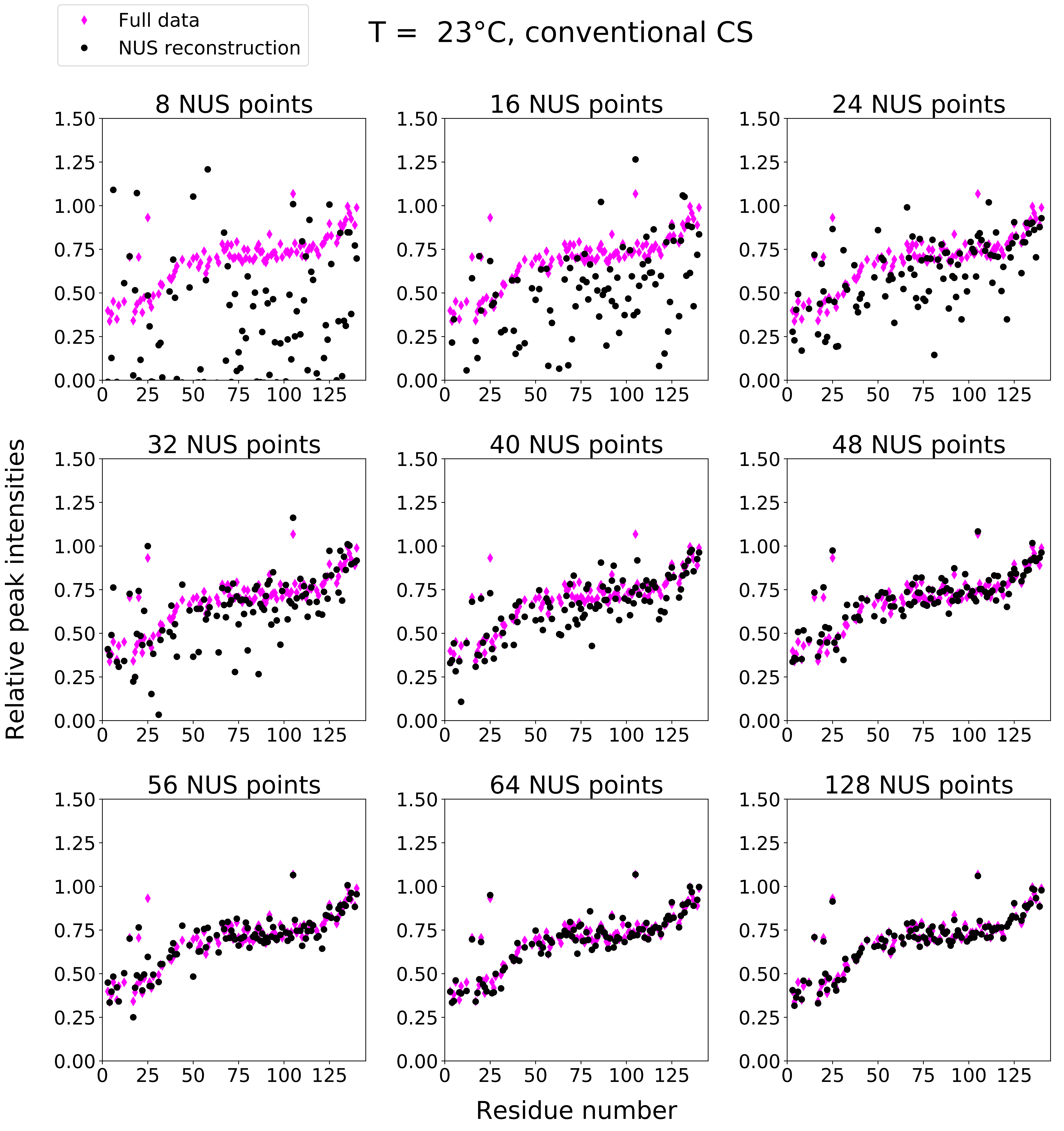

Full data
NUS reconstruction
T = 23°C, conventional CS
8 NUS points
16 NUS points
24 NUS points
32 NUS points
40 NUS points
48 NUS points
56 NUS points
64 NUS points
128 NUS points
Relative peak intensities
Residue number

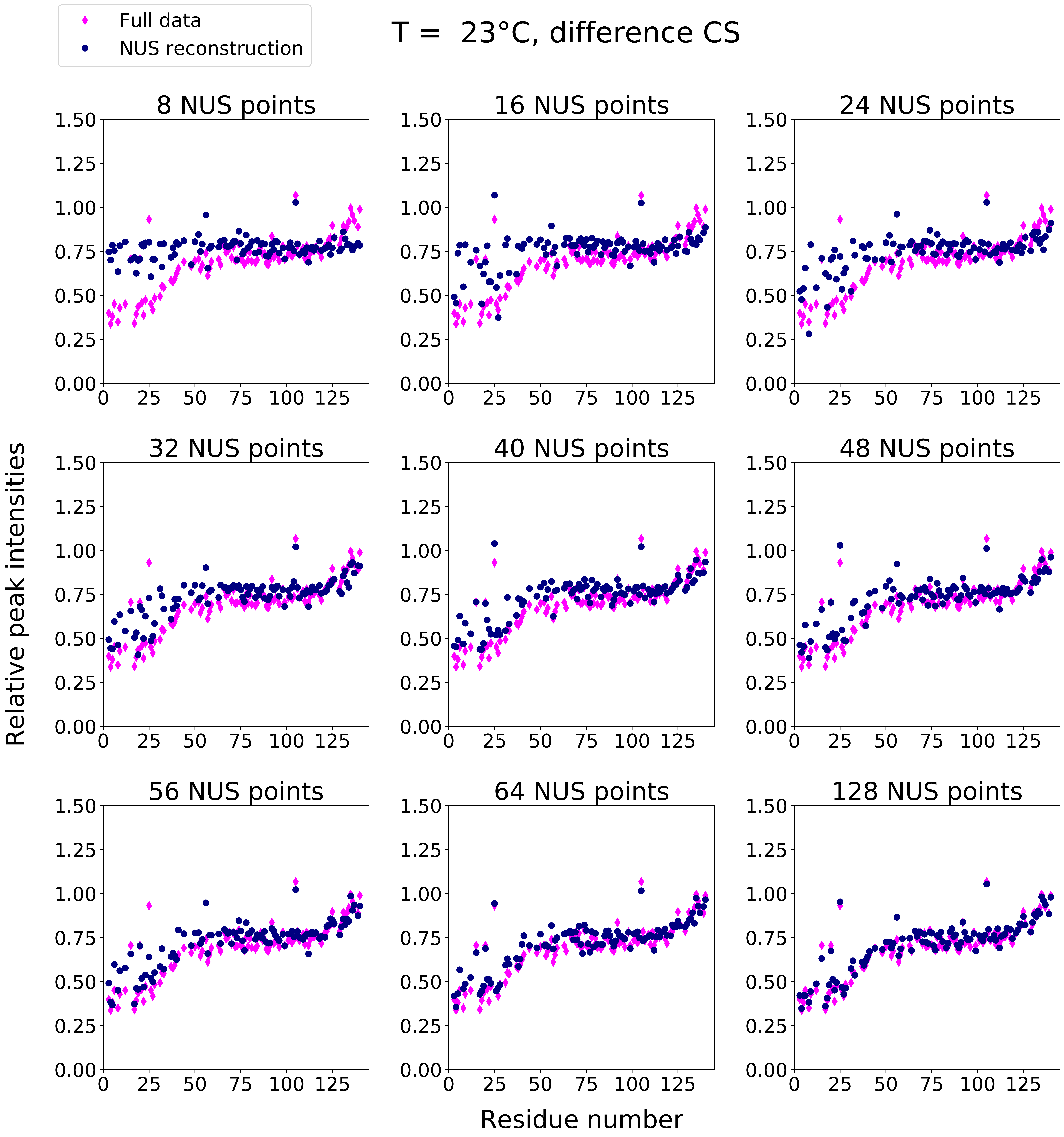

Full data
NUS reconstruction
T = 23°C, difference CS
8 NUS points
16 NUS points
24 NUS points
32 NUS points
40 NUS points
48 NUS points
56 NUS points
64 NUS points
128 NUS points
Relative peak intensities
Residue number

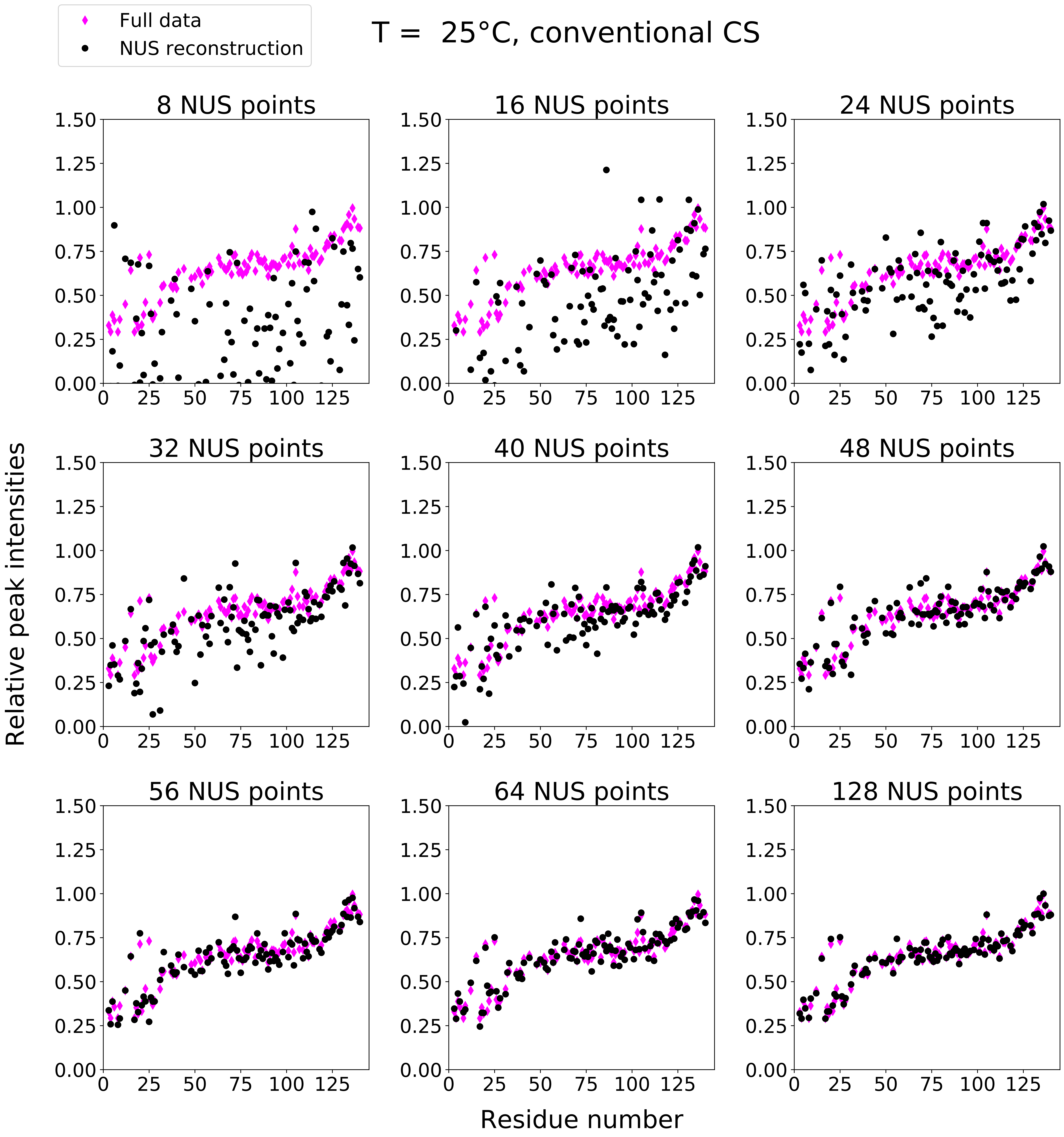

Full data
NUS reconstruction
T = 25°C, conventional CS
8 NUS points
16 NUS points
24 NUS points
32 NUS points
40 NUS points
48 NUS points
56 NUS points
64 NUS points
128 NUS points
Relative peak intensities
Residue number
0.00
0.25
0.50
0.75
1.00
1.25
1.50
0
25
50
75
100
125

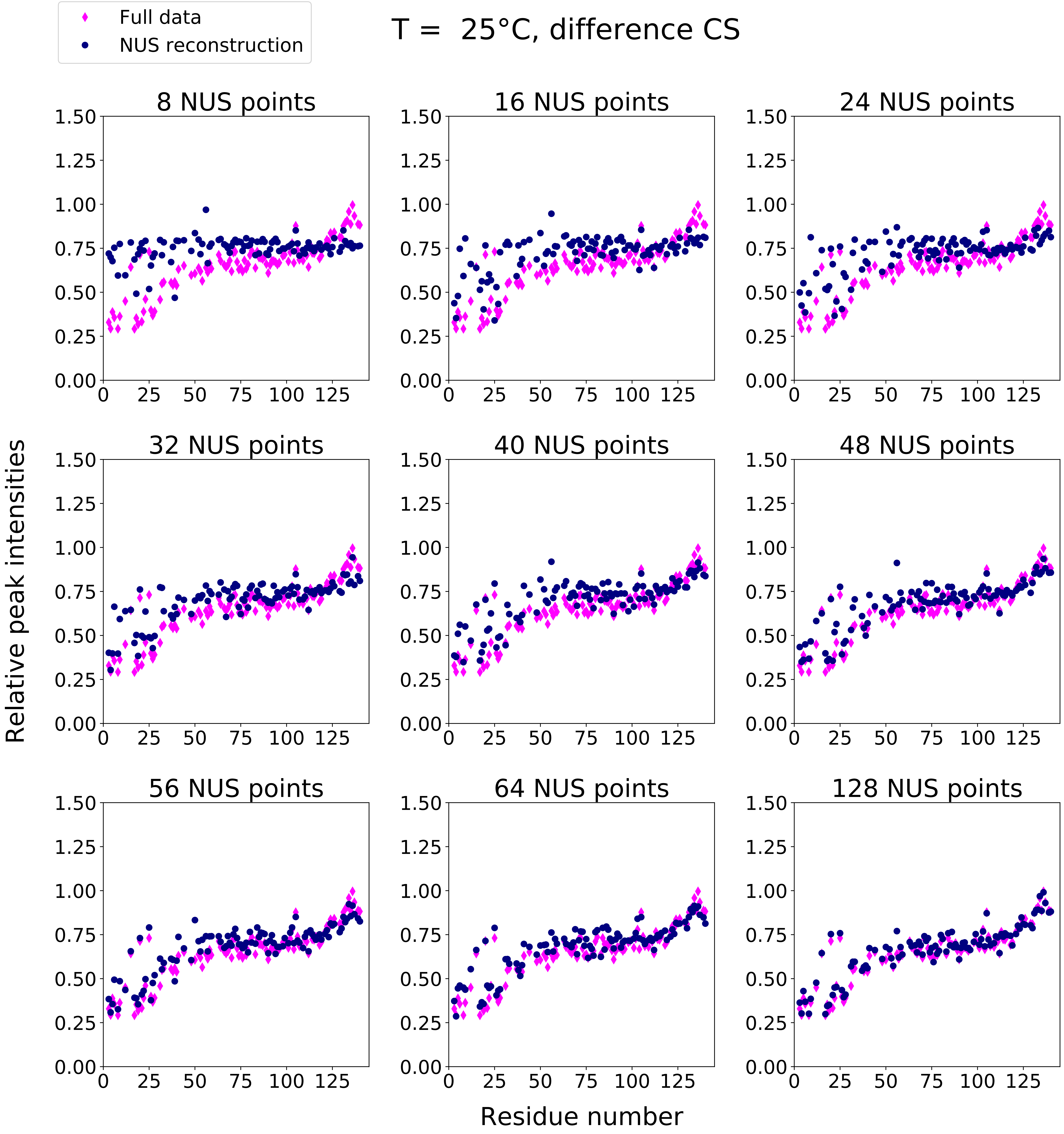
Full data
NUS reconstruction
T = 25°C, difference CS
8 NUS points
16 NUS points
24 NUS points
32 NUS points
40 NUS points
48 NUS points
56 NUS points
64 NUS points
128 NUS points
Relative peak intensities
Residue number
0.00
0.25
0.50
0.75
1.00
1.25
1.50
0
25
50
75
100
125

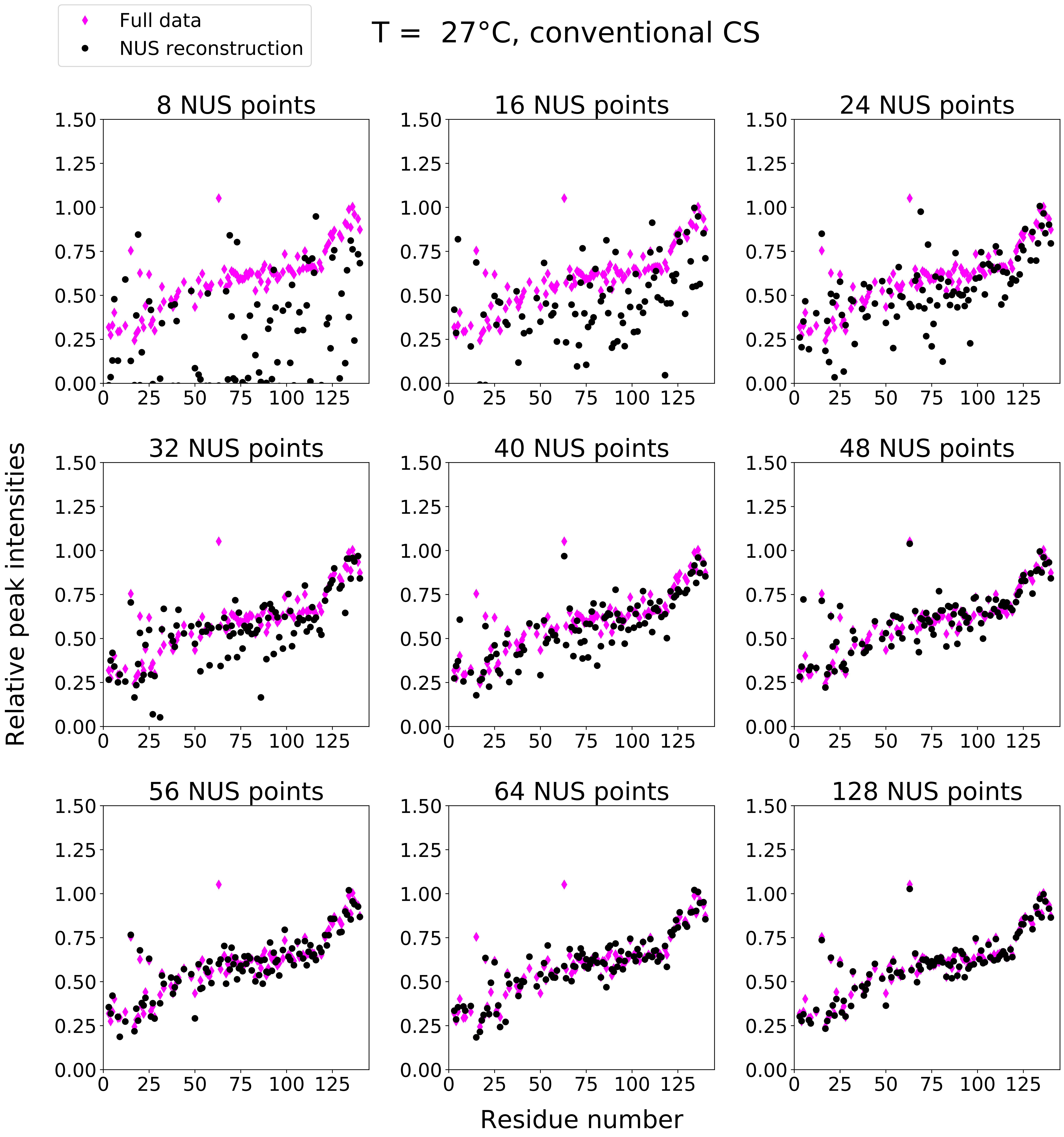
Full data
NUS reconstruction
T = 27°C, conventional CS
8 NUS points
16 NUS points
24 NUS points
32 NUS points
40 NUS points
48 NUS points
56 NUS points
64 NUS points
128 NUS points
Relative peak intensities
Residue number

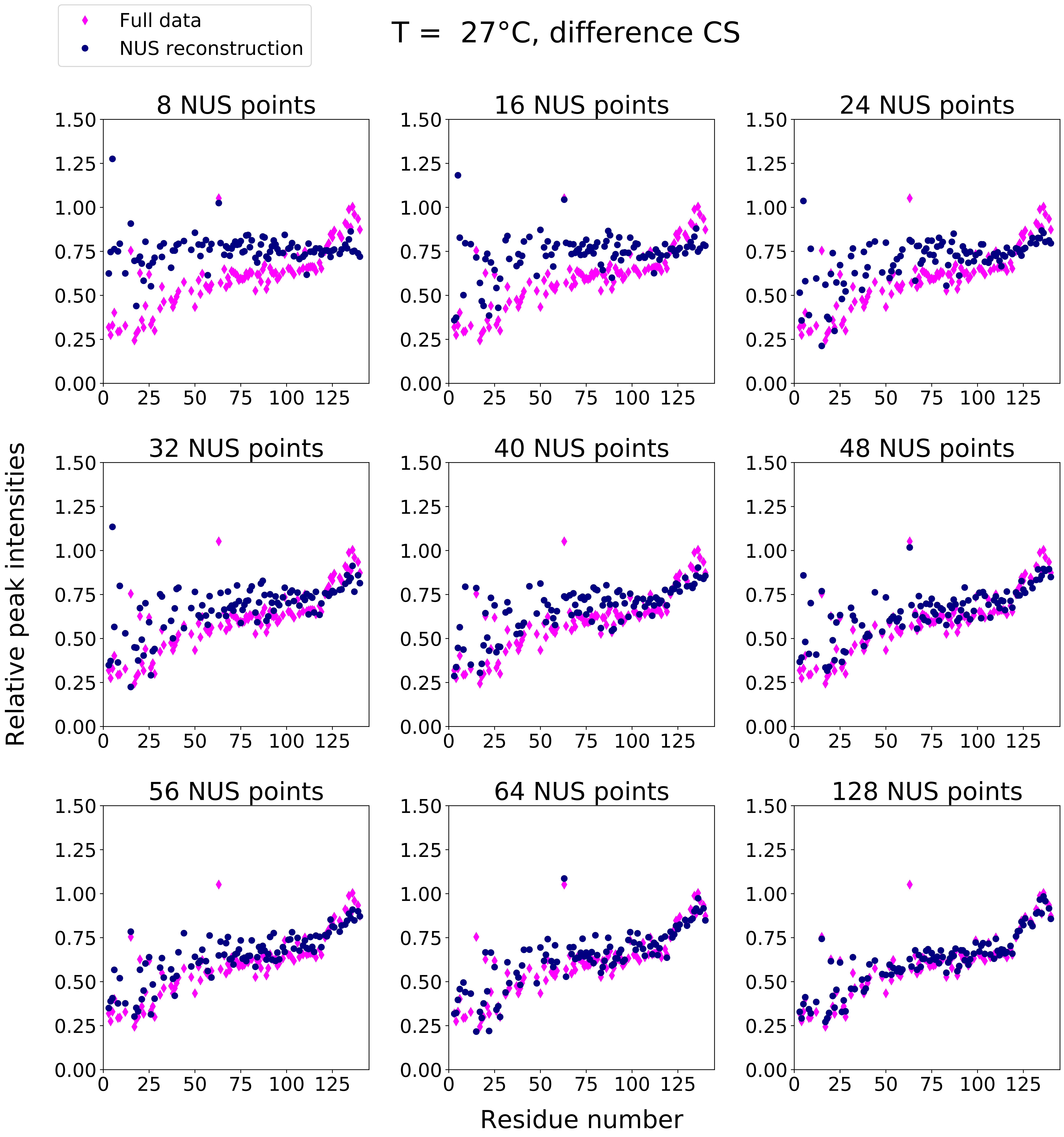

Full data
NUS reconstruction
T = 27°C, difference CS
8 NUS points
16 NUS points
24 NUS points
32 NUS points
40 NUS points
48 NUS points
56 NUS points
64 NUS points
128 NUS points
Relative peak intensities
Residue number
0.00
0.25
0.50
0.75
1.00
1.25
1.50
0
25
50
75
100
125

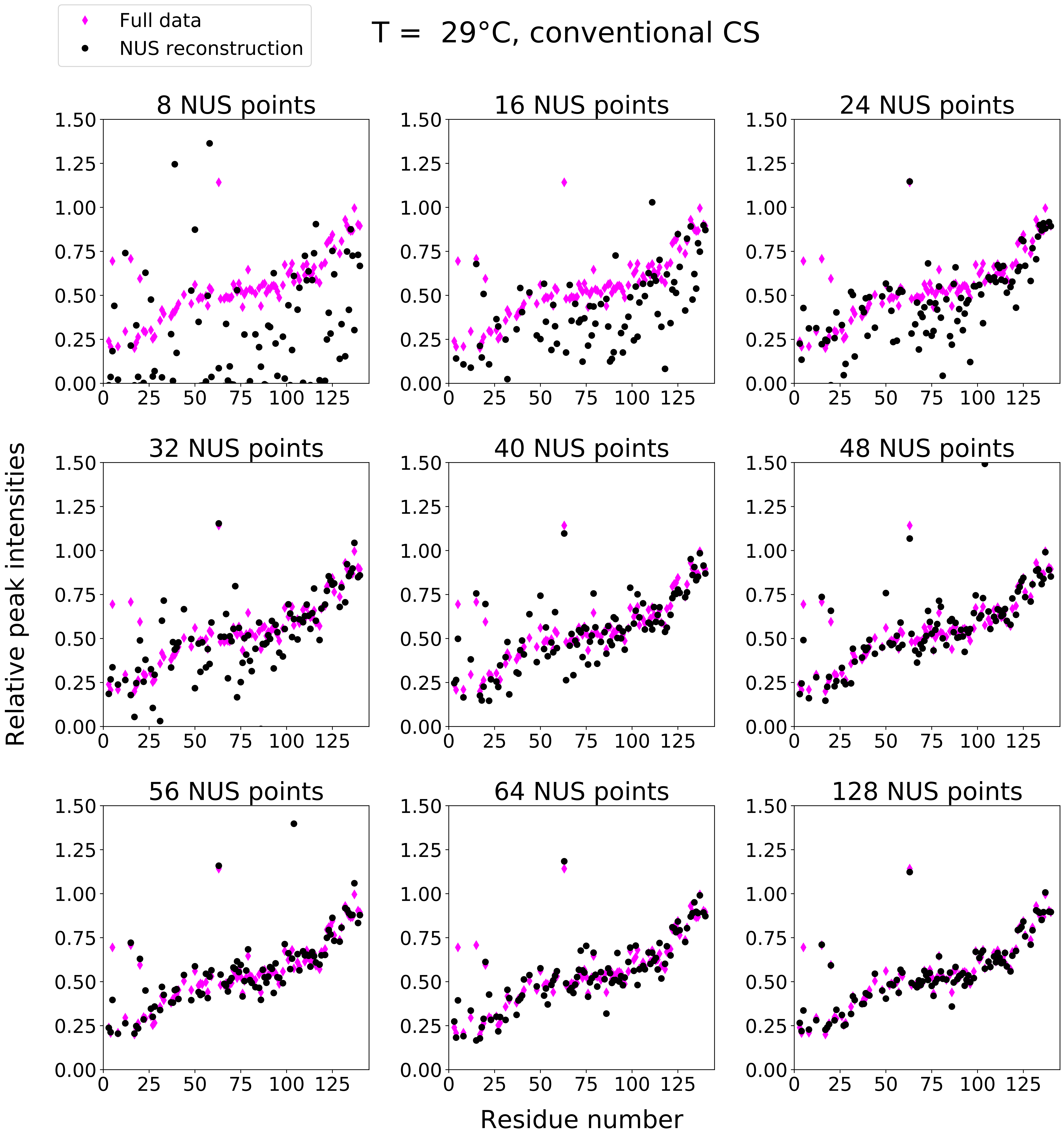
Full data
NUS reconstruction
T = 29°C, conventional CS
8 NUS points
16 NUS points
24 NUS points
32 NUS points
40 NUS points
48 NUS points
56 NUS points
64 NUS points
128 NUS points
Relative peak intensities
Residue number

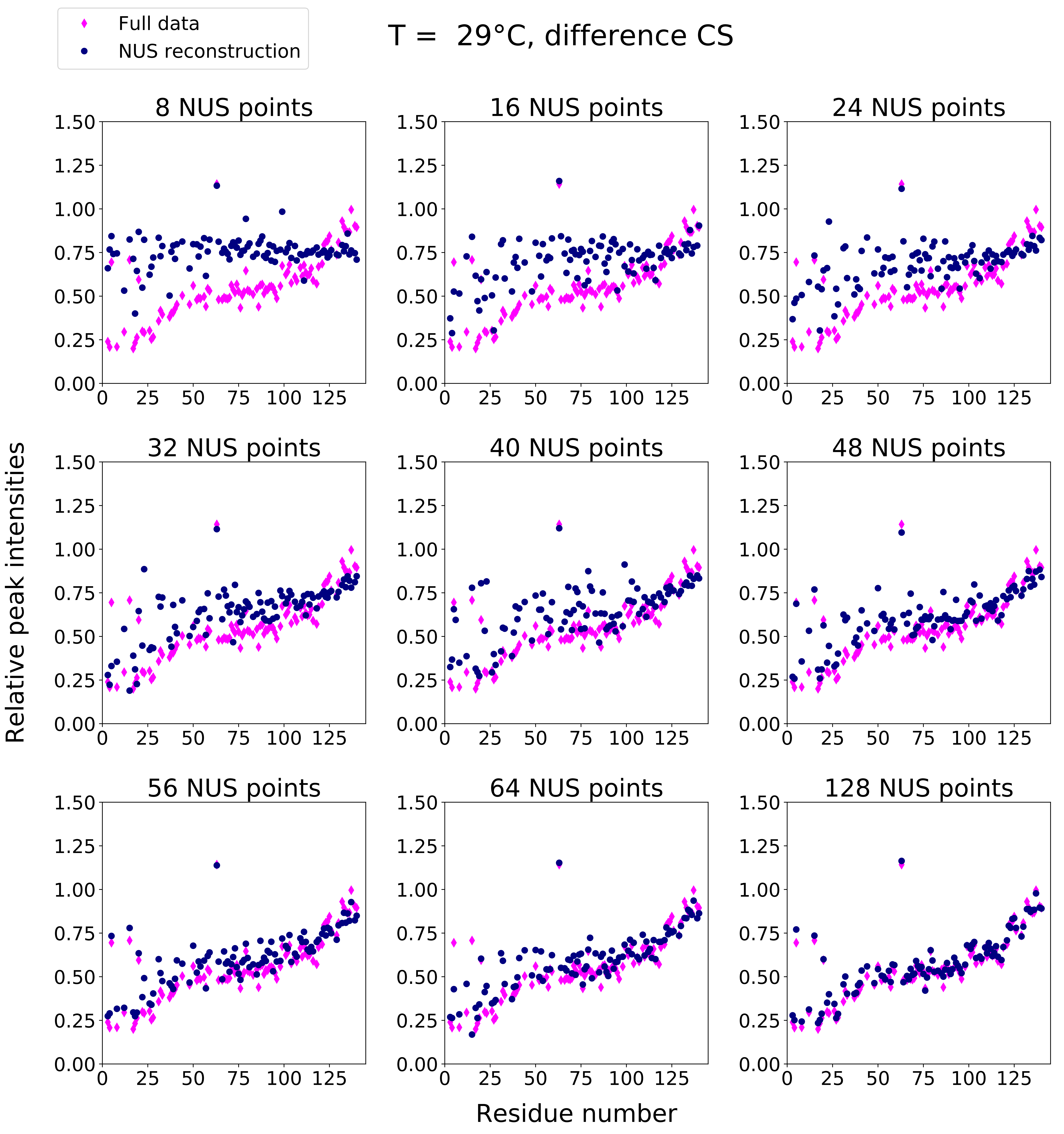

Full data
NUS reconstruction
T = 29°C, difference CS
8 NUS points
16 NUS points
24 NUS points
32 NUS points
40 NUS points
48 NUS points
56 NUS points
64 NUS points
128 NUS points
Relative peak intensities
Residue number

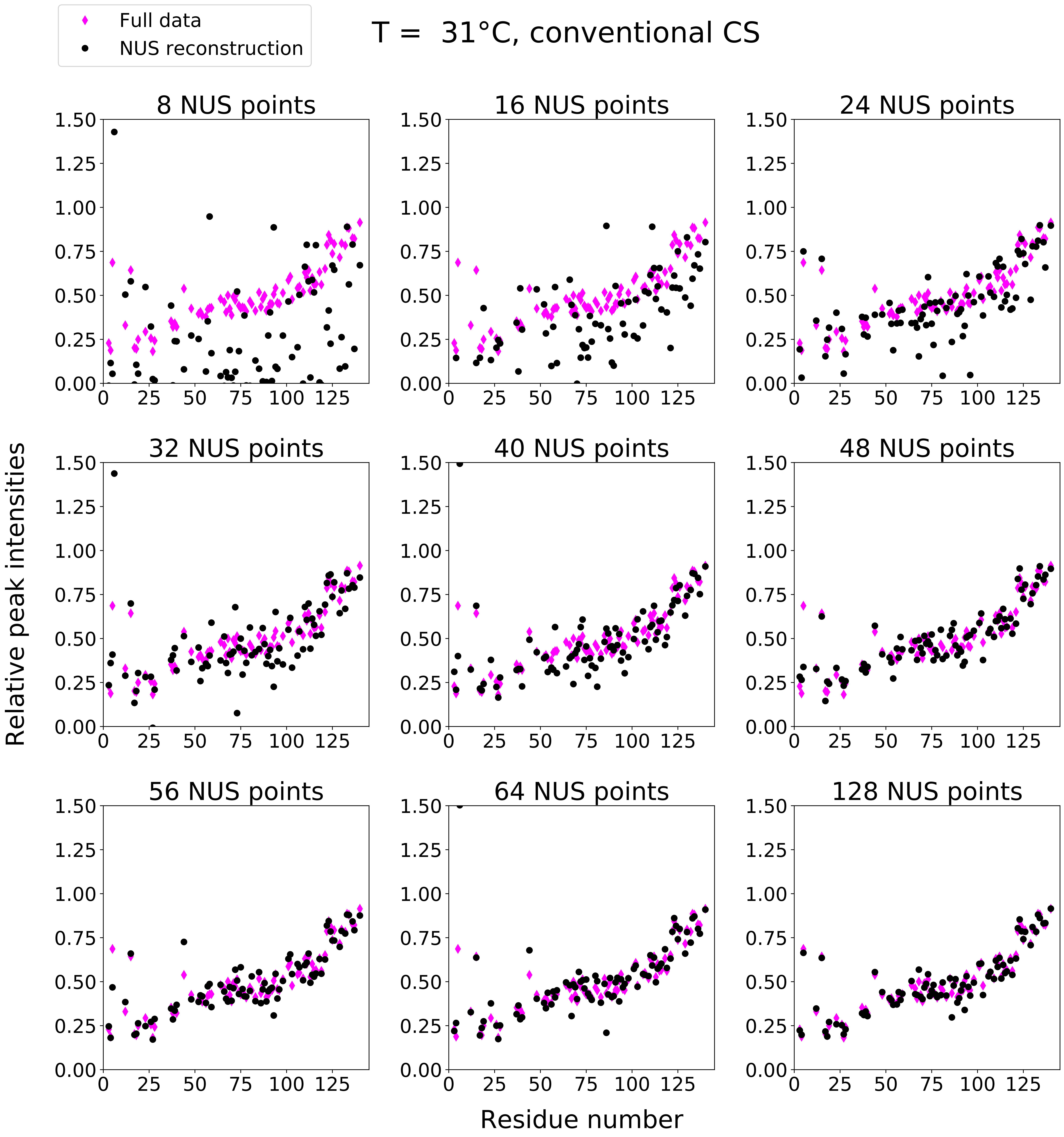
Full data
NUS reconstruction
T = 31°C, conventional CS
8 NUS points
16 NUS points
24 NUS points
32 NUS points
40 NUS points
48 NUS points
56 NUS points
64 NUS points
128 NUS points
Relative peak intensities
Residue number

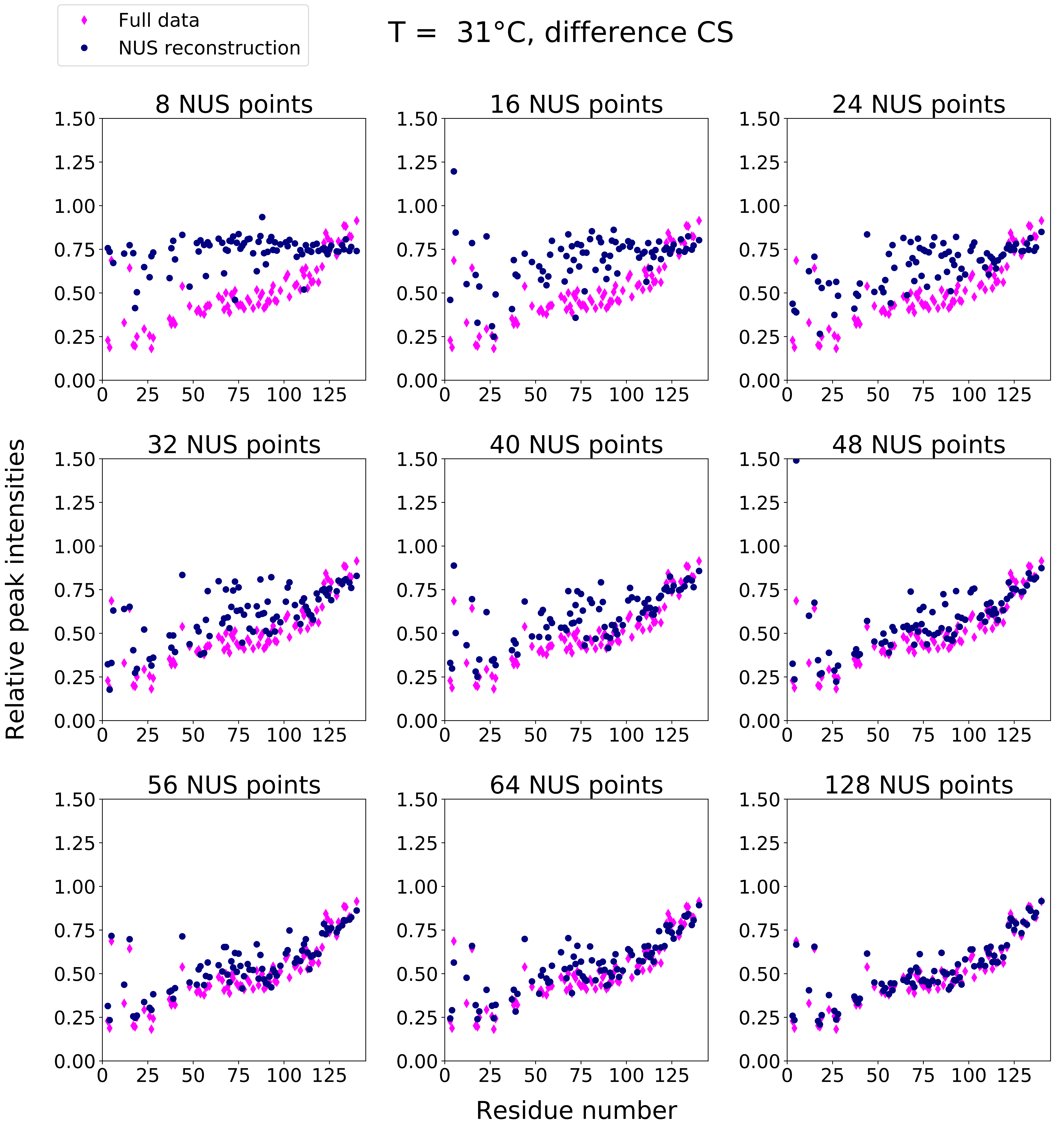

Full data
NUS reconstruction
T = 31°C, difference CS
8 NUS points
16 NUS points
24 NUS points
32 NUS points
40 NUS points
48 NUS points
56 NUS points
64 NUS points
128 NUS points
Relative peak intensities
Residue number

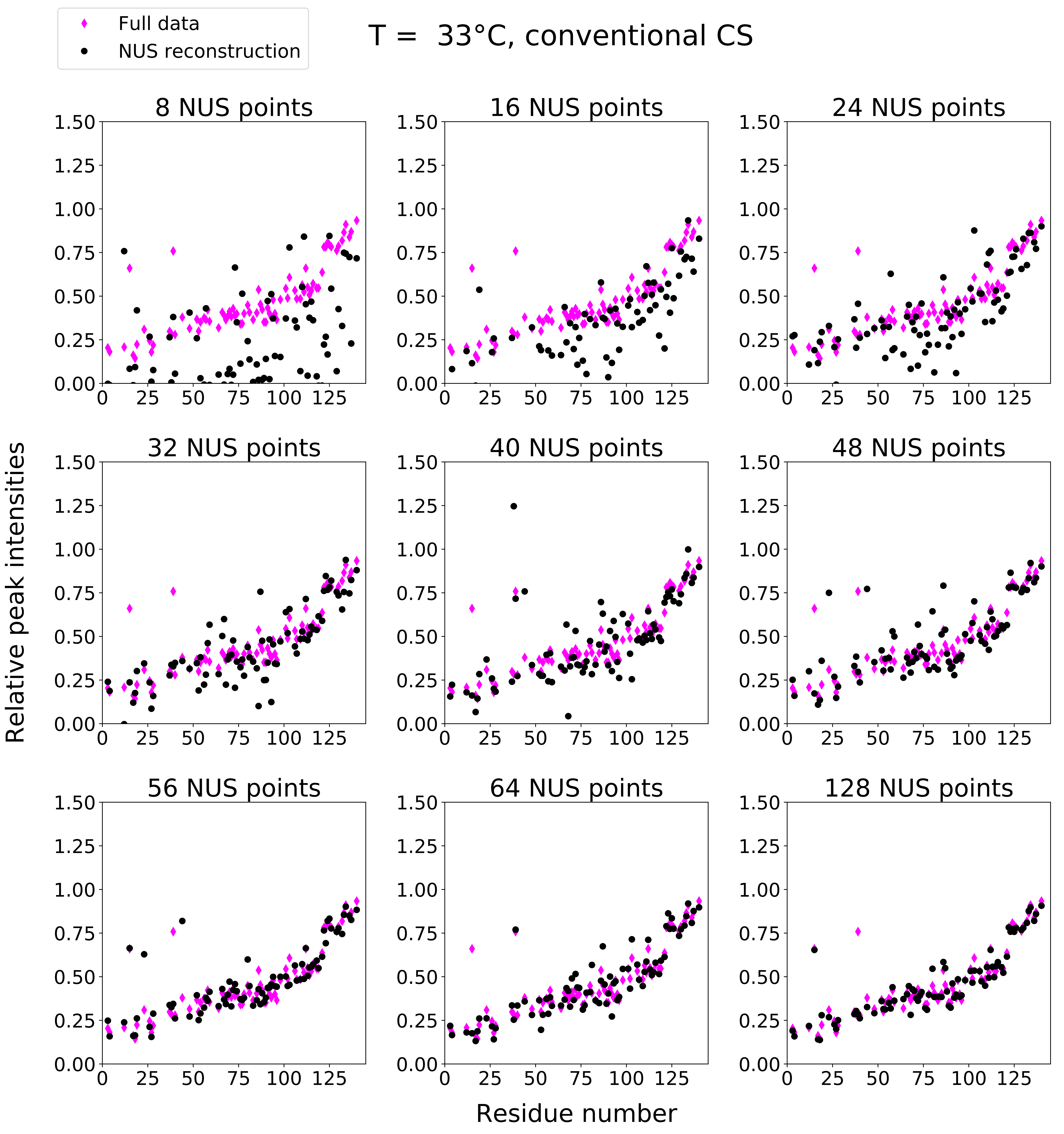

Full data
NUS reconstruction
T = 33°C, conventional CS
8 NUS points
16 NUS points
24 NUS points
32 NUS points
40 NUS points
48 NUS points
56 NUS points
64 NUS points
128 NUS points
Relative peak intensities
Residue number

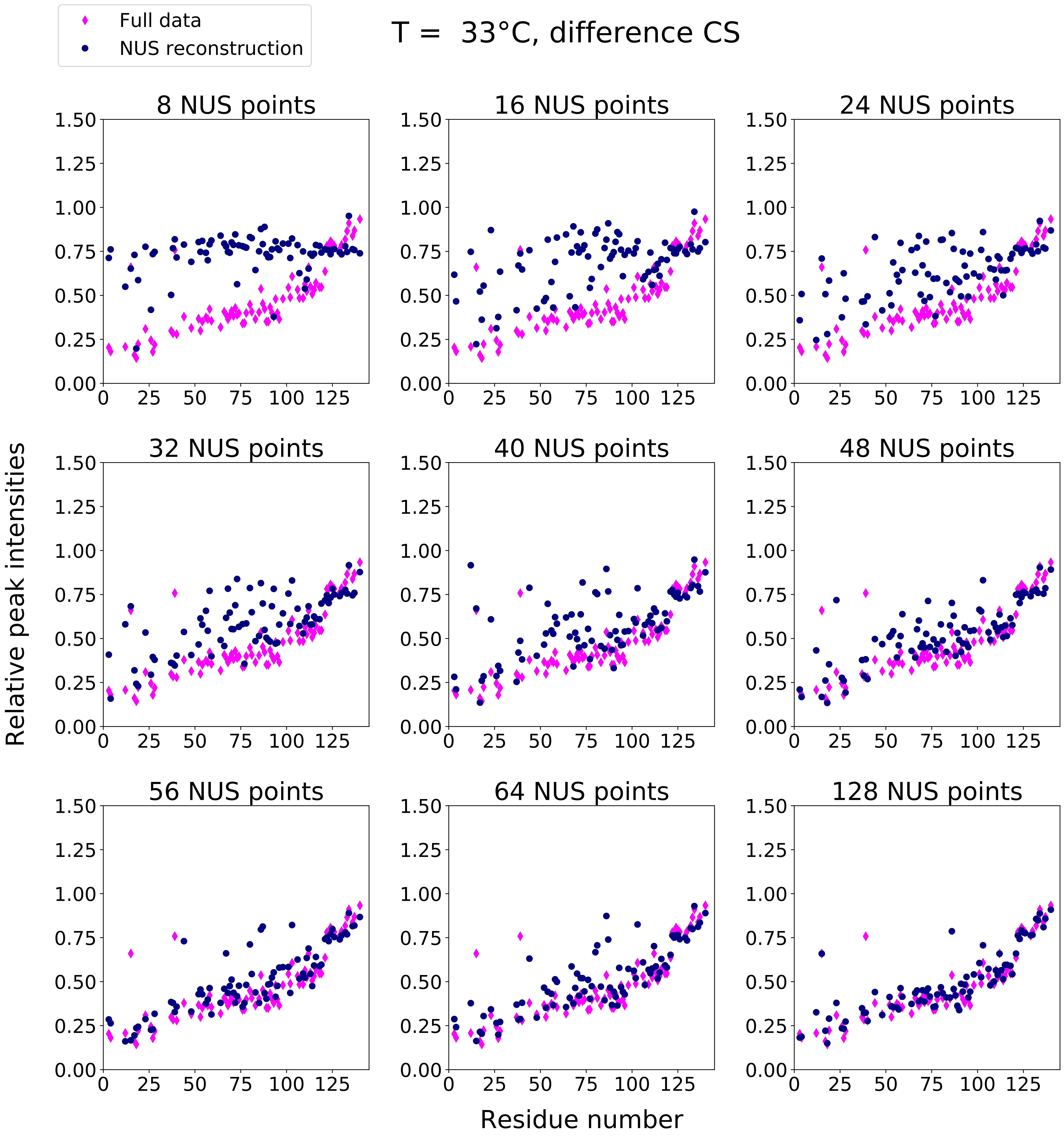

Full data
NUS reconstruction
T = 33°C, difference CS
8 NUS points
16 NUS points
24 NUS points
32 NUS points
40 NUS points
48 NUS points
56 NUS points
64 NUS points
128 NUS points
Relative peak intensities
Residue number

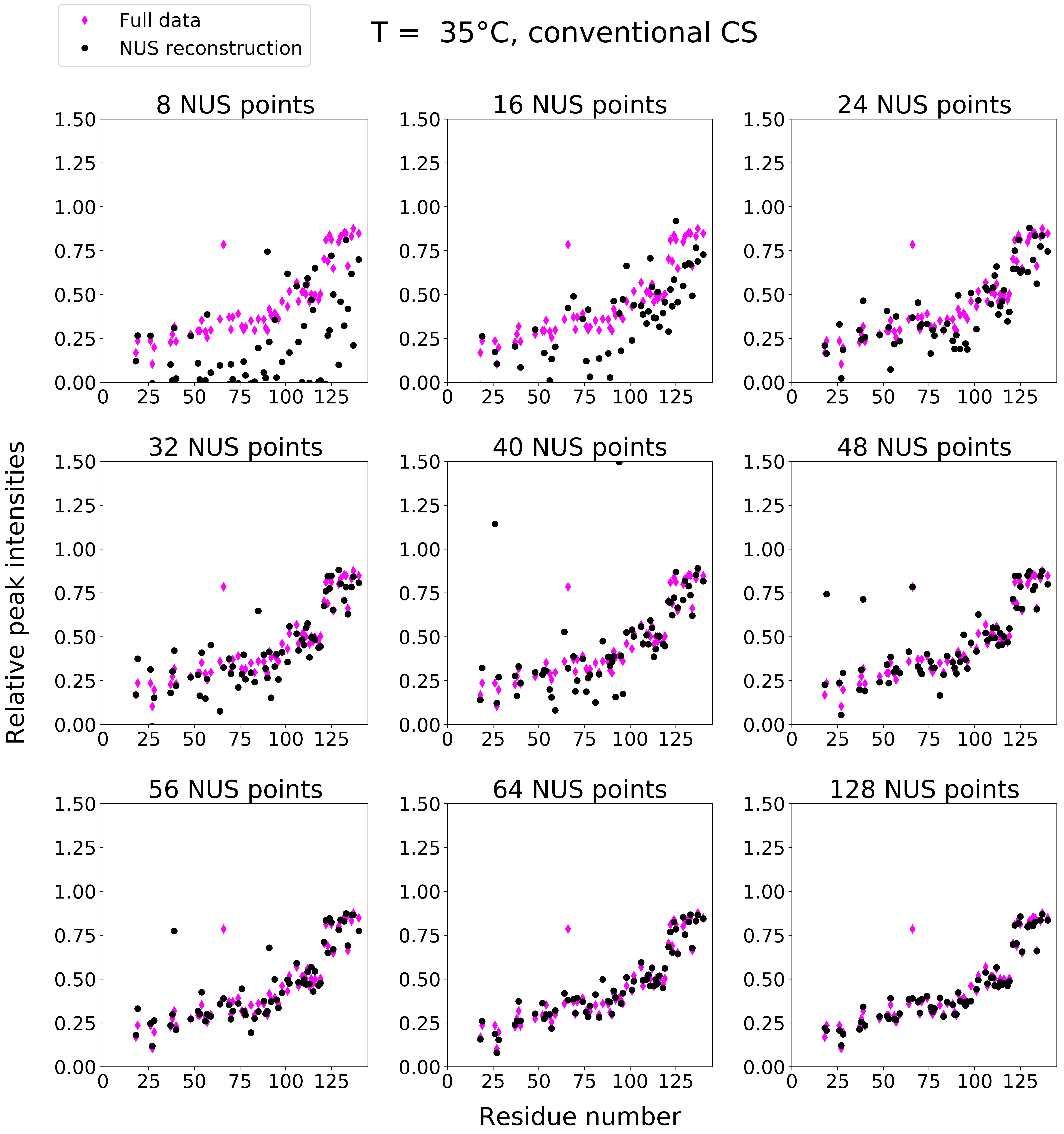
Full data
NUS reconstruction
T = 35°C, conventional CS
8 NUS points
16 NUS points
24 NUS points
32 NUS points
40 NUS points
48 NUS points
56 NUS points
64 NUS points
128 NUS points
Relative peak intensities
Residue number

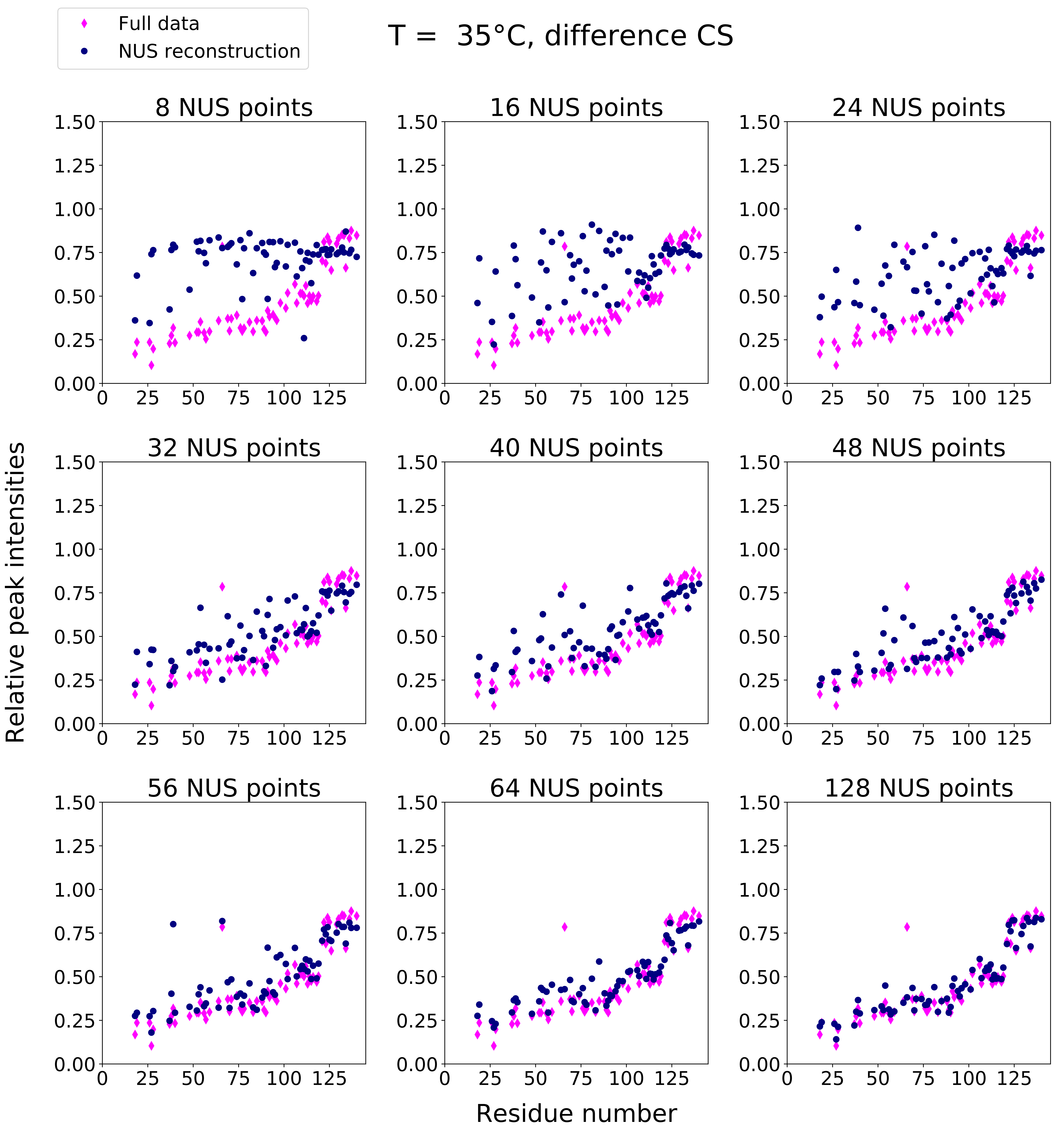

Full data
NUS reconstruction
T = 35°C, difference CS
8 NUS points
16 NUS points
24 NUS points
32 NUS points
40 NUS points
48 NUS points
56 NUS points
64 NUS points
128 NUS points
Relative peak intensities
Residue number
1.50
1.25
1.00
0.75
0.50
0.25
0.00
0
25
50
75
100
125

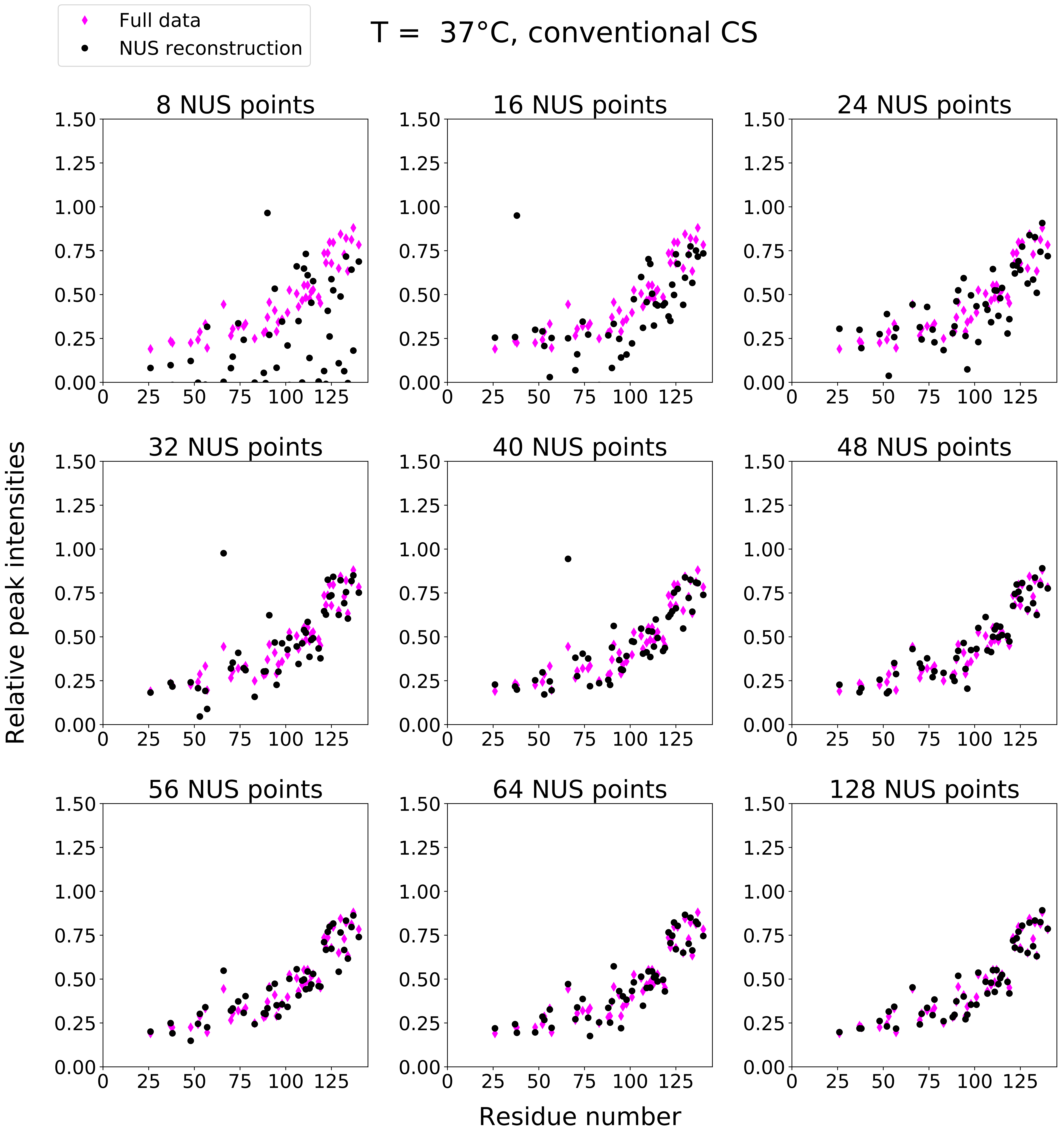

Full data
NUS reconstruction
T = 37°C, conventional CS
8 NUS points
16 NUS points
24 NUS points
32 NUS points
40 NUS points
48 NUS points
56 NUS points
64 NUS points
128 NUS points
Relative peak intensities
Residue number

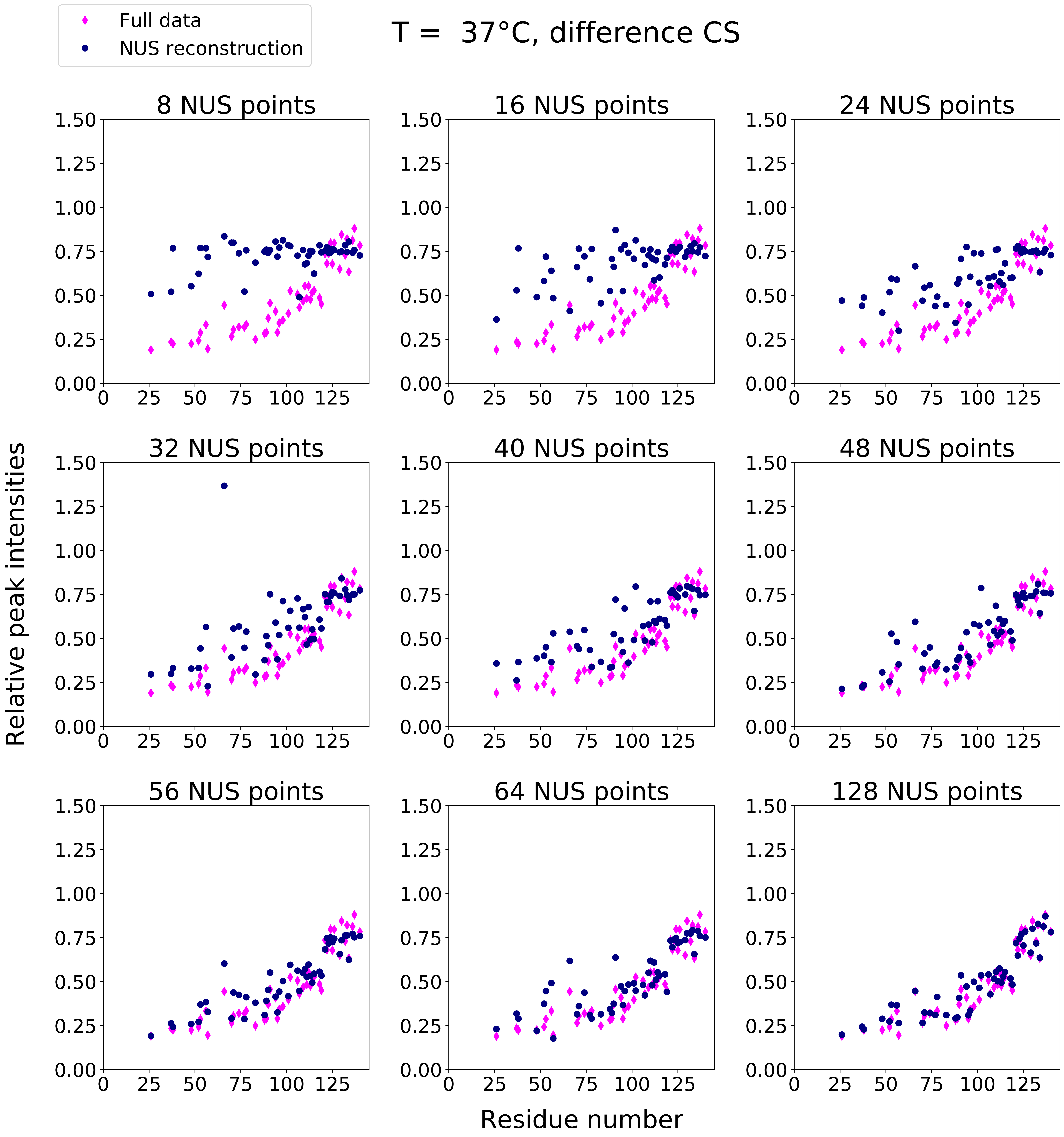

Full data
NUS reconstruction
T = 37°C, difference CS
8 NUS points
16 NUS points
24 NUS points
32 NUS points
40 NUS points
48 NUS points
56 NUS points
64 NUS points
128 NUS points
Relative peak intensities
Residue number

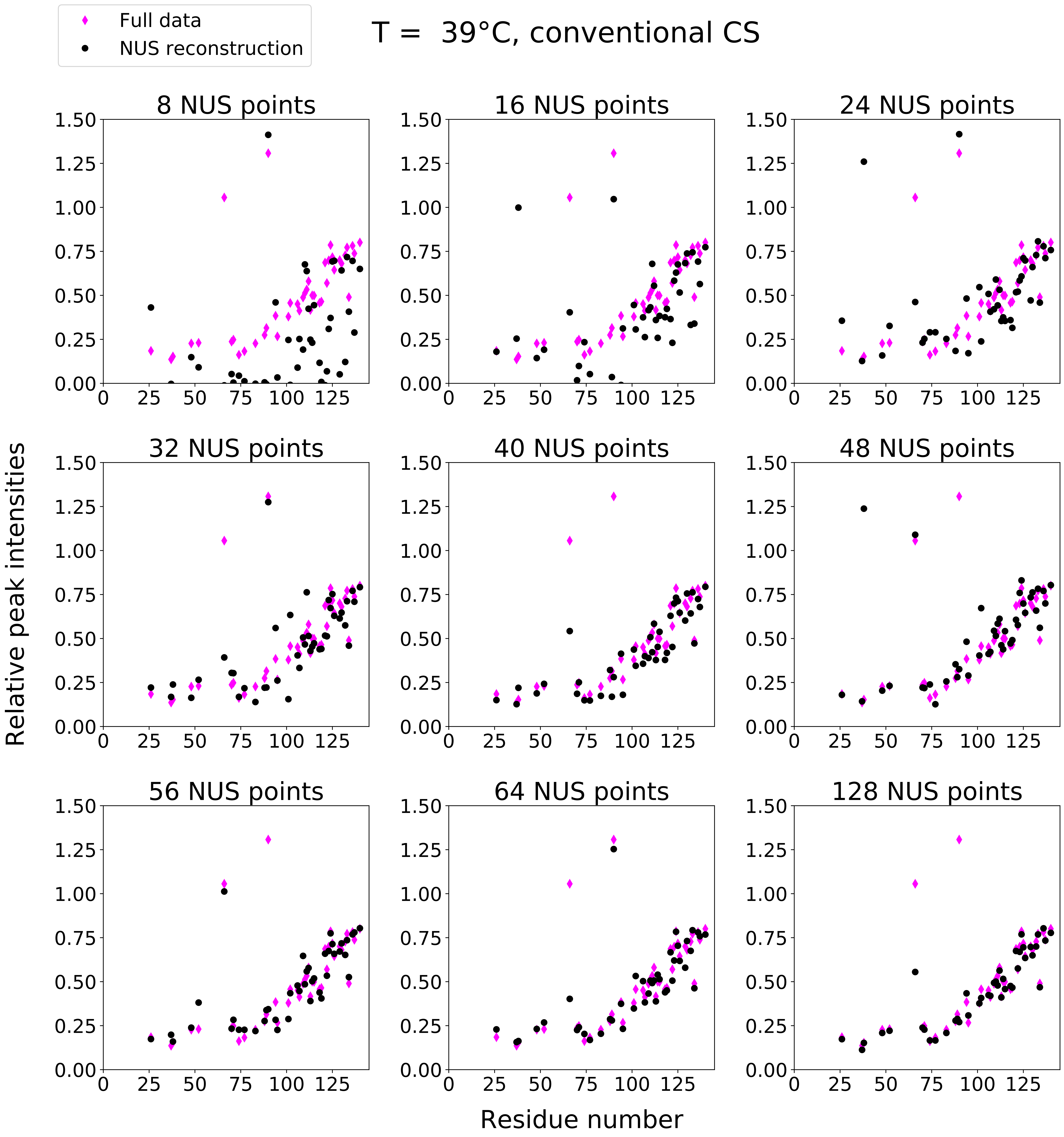
T = 39°C, conventional CS
Full data
NUS reconstruction
8 NUS points
16 NUS points
24 NUS points
32 NUS points
40 NUS points
48 NUS points
56 NUS points
64 NUS points
128 NUS points
Relative peak intensities
Residue number

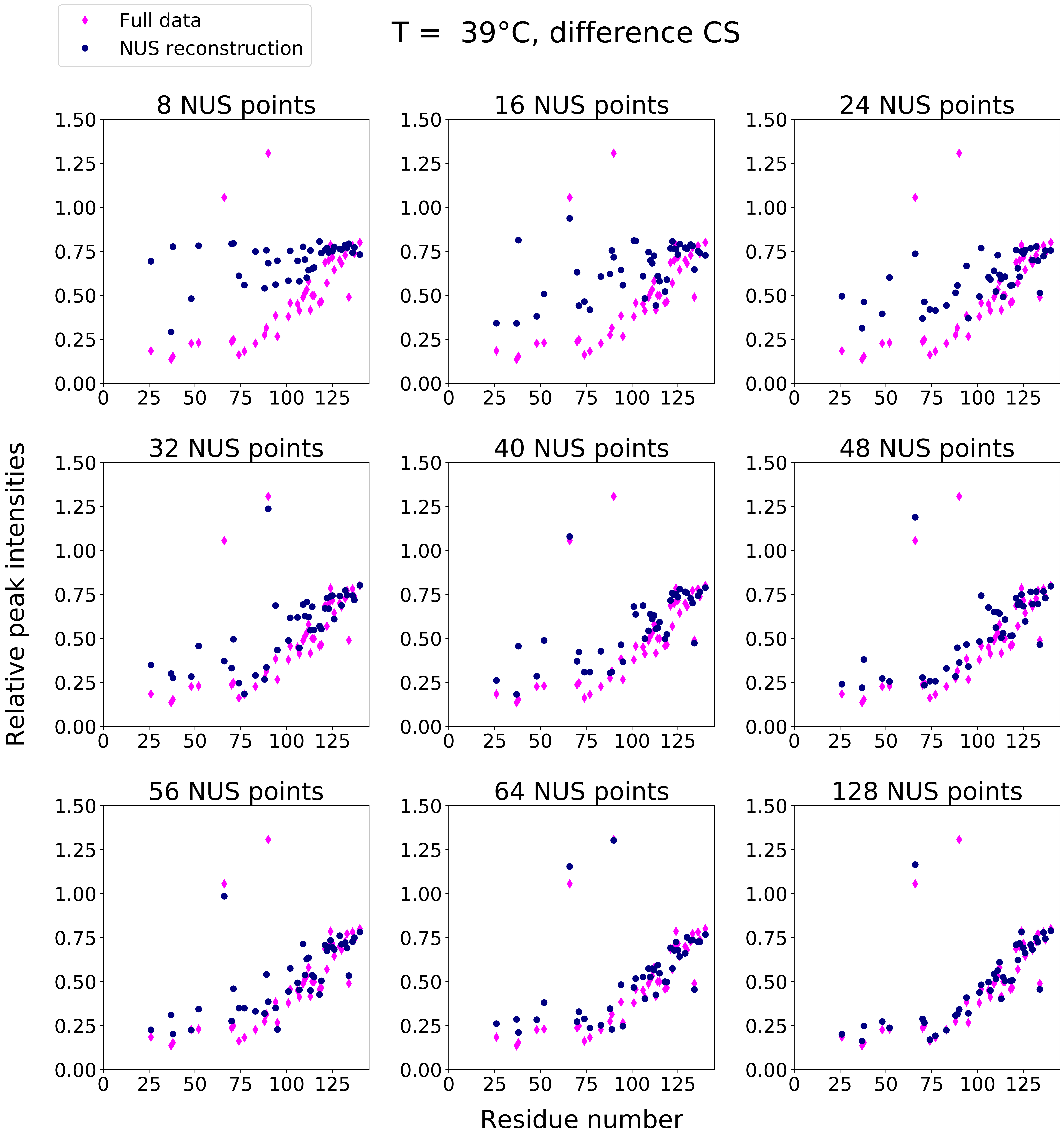

Full data
NUS reconstruction
T = 39°C, difference CS
8 NUS points
16 NUS points
24 NUS points
32 NUS points
40 NUS points
48 NUS points
56 NUS points
64 NUS points
128 NUS points
Relative peak intensities
Residue number

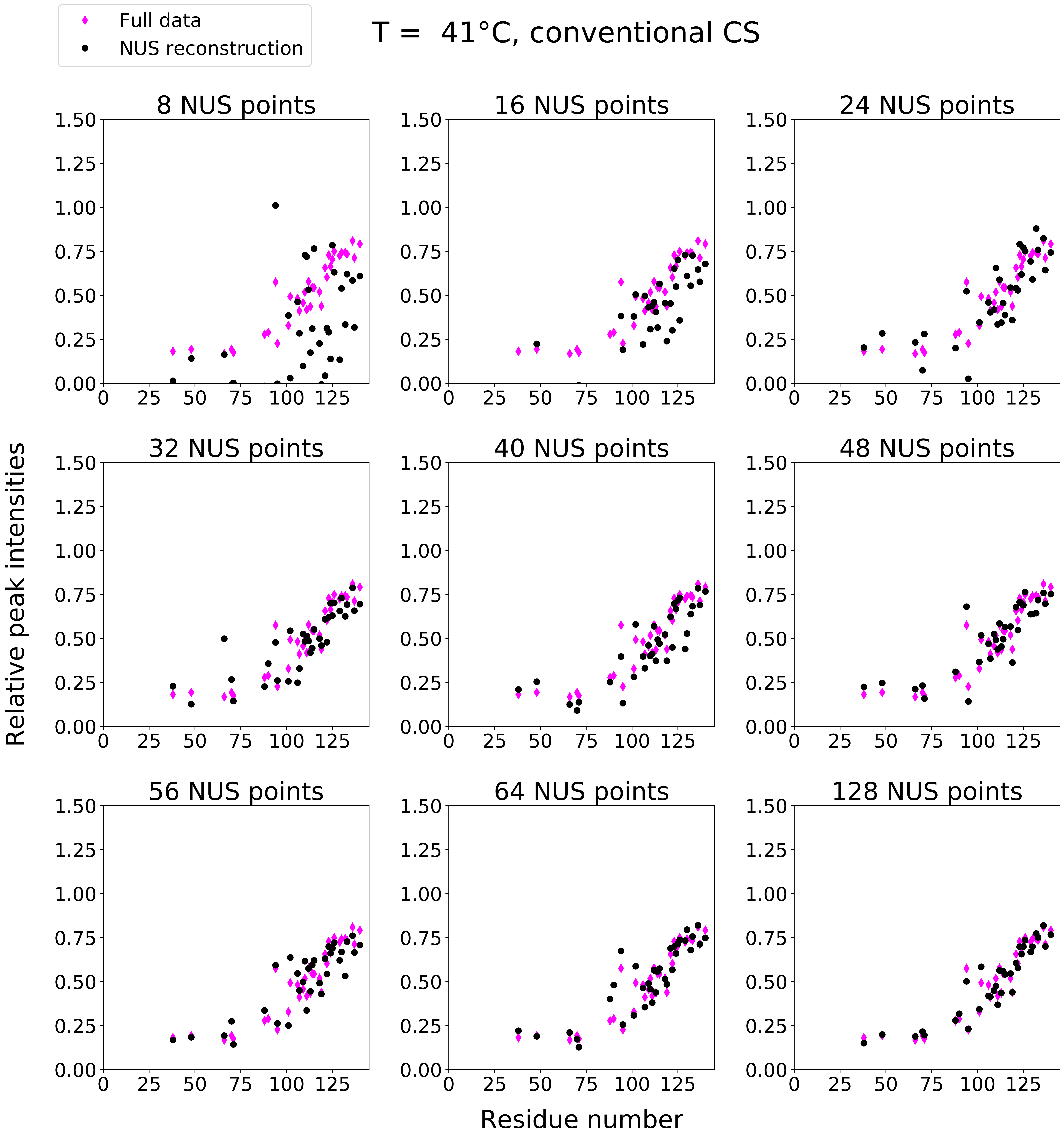
Full data
NUS reconstruction
T = 41°C, conventional CS
8 NUS points
16 NUS points
24 NUS points
32 NUS points
40 NUS points
48 NUS points
56 NUS points
64 NUS points
128 NUS points
Relative peak intensities
Residue number
1.50
1.25
1.00
0.75
0.50
0.25
0.00
0
25
50
75
100
125

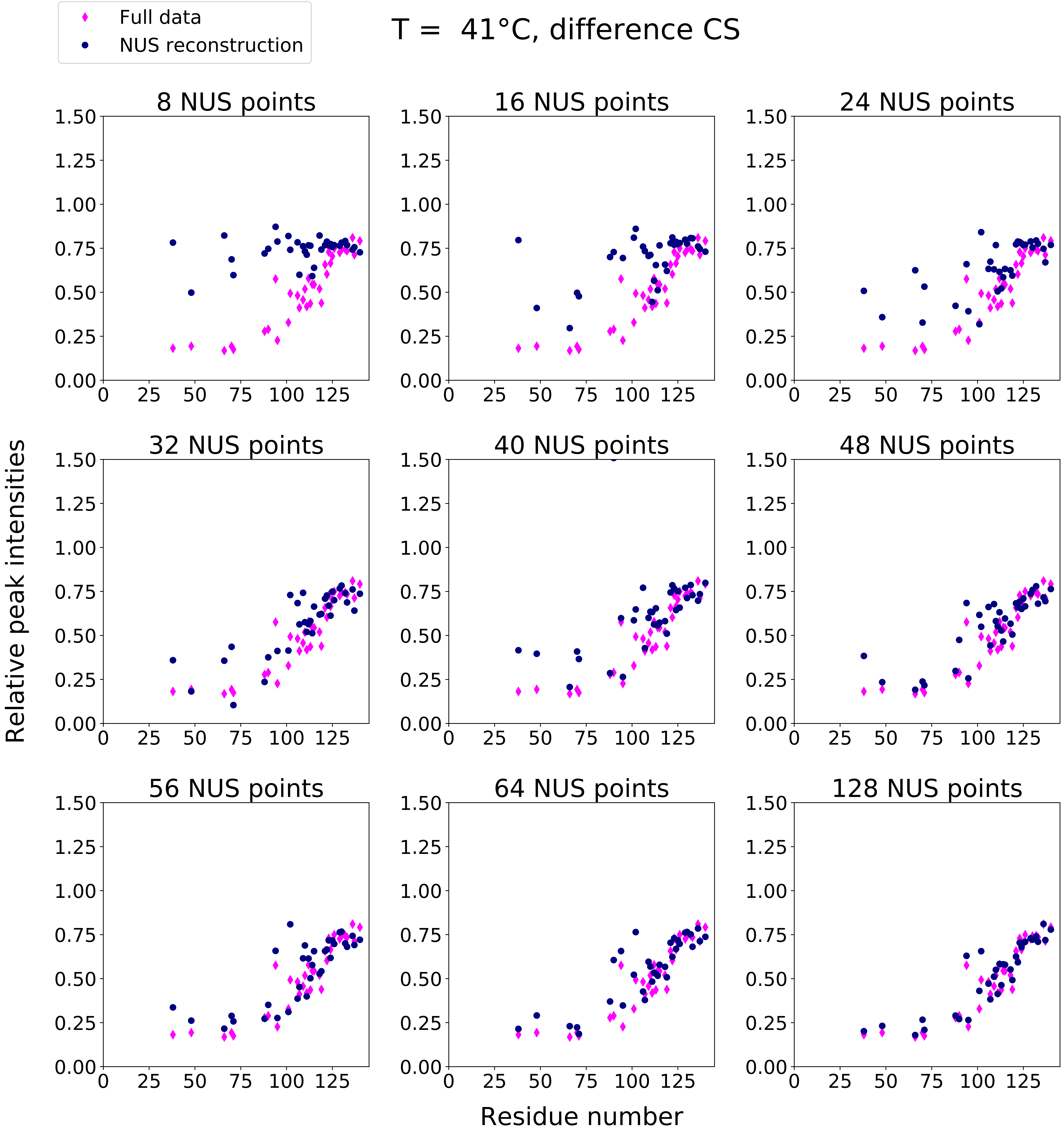

Full data
NUS reconstruction
T = 41°C, difference CS
8 NUS points
16 NUS points
24 NUS points
32 NUS points
40 NUS points
48 NUS points
56 NUS points
64 NUS points
128 NUS points
Relative peak intensities
Residue number

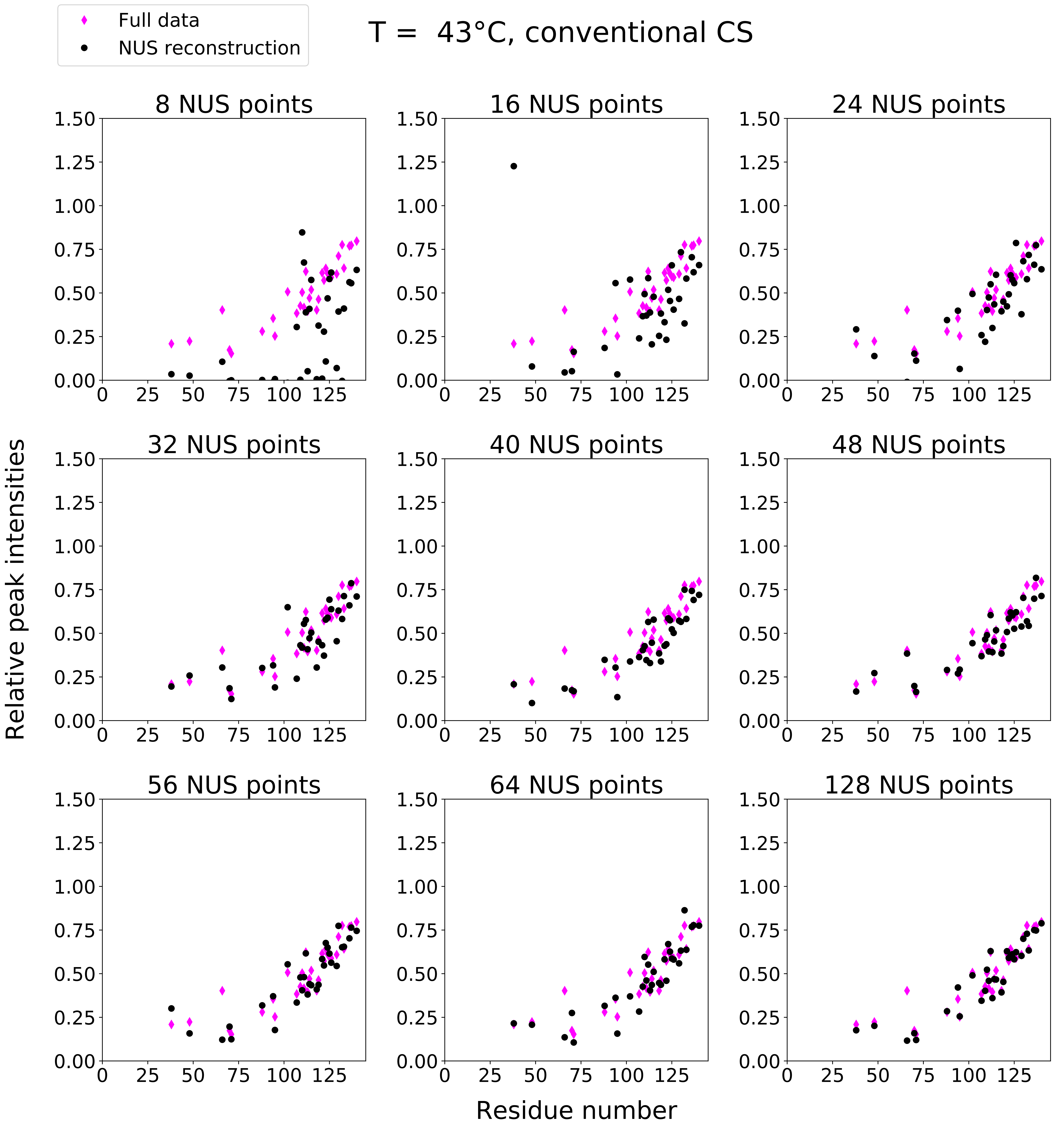
Full data
NUS reconstruction
T = 43°C, conventional CS
8 NUS points
16 NUS points
24 NUS points
32 NUS points
40 NUS points
48 NUS points
56 NUS points
64 NUS points
128 NUS points
Relative peak intensities
Residue number

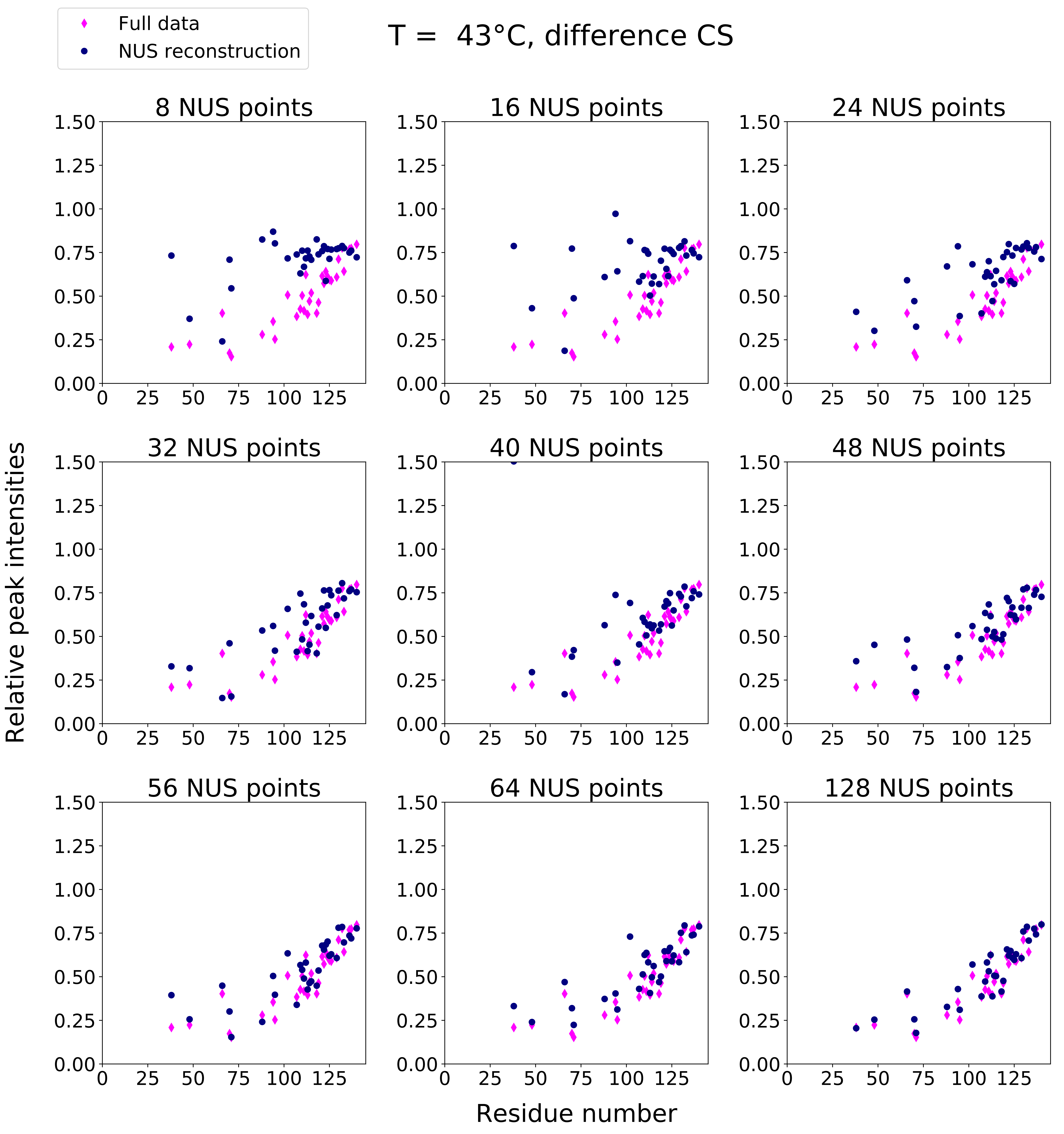

Full data
NUS reconstruction
T = 43°C, difference CS
8 NUS points
16 NUS points
24 NUS points
32 NUS points
40 NUS points
48 NUS points
56 NUS points
64 NUS points
128 NUS points
Relative peak intensities
Residue number
1.50
1.25
1.00
0.75
0.50
0.25
0.00
0
25
50
75
100
125

Table S1: Normalized residuals of peak heights: CS reconstruction

	8 NUS points	16 NUS points	24 NUS points	32 NUS points	40 NUS points	48 NUS points	56 NUS points	64 NUS points	128 NUS points
15°C	0.721	0.463	0.220	0.183	0.125	0.060	0.075	0.041	0.060
17°C	0.733	0.475	0.226	0.170	0.093	0.091	0.100	0.035	0.017
19°C	0.717	0.481	0.229	0.196	0.141	0.112	0.058	0.094	0.019
21°C	0.718	0.503	0.255	0.173	0.113	0.056	0.063	0.052	0.024
23°C	0.722	0.491	0.233	0.195	0.135	0.066	0.077	0.045	0.024
25°C	0.707	0.510	0.219	0.201	0.138	0.070	0.333	0.054	0.030
27°C	0.726	0.514	0.259	0.189	0.169	0.323	0.317	0.350	0.313
29°C	0.749	0.630	0.302	0.224	0.121	0.177	0.157	0.129	0.073
31°C	0.658	0.616	0.233	0.206	0.154	0.115	0.266	0.189	0.058
33°C	0.577	0.725	0.251	0.199	0.260	0.198	0.147	0.125	0.101
35°C	0.678	0.459	0.404	0.209	0.403	0.182	0.182	0.357	0.107
37°C	0.667	0.390	0.204	0.377	0.178	0.095	0.080	0.083	0.043
39°C	0.594	0.421	0.395	0.236	0.330	0.409	0.277	0.192	0.316
41°C	0.556	0.324	0.496	0.174	0.459	0.461	0.438	0.100	0.060
43°C	0.619	0.471	0.248	0.177	0.172	0.112	0.137	0.140	0.108

Table S2: Normalized residuals of peak heights: DCS reconstruction

	8 NUS points	16 NUS points	24 NUS points	32 NUS points	40 NUS points	48 NUS points	56 NUS points	64 NUS points	128 NUS points
15°C	0.136	0.105	0.091	0.087	0.081	0.071	0.070	0.067	0.055
17°C	0.136	0.102	0.084	0.085	0.068	0.056	0.051	0.040	0.021
19°C	0.150	0.111	0.133	0.078	0.064	0.106	0.108	0.046	0.026
21°C	0.180	0.131	0.138	0.126	0.108	0.098	0.105	0.055	0.027
23°C	0.214	0.172	0.159	0.119	0.100	0.087	0.094	0.067	0.043
25°C	0.262	0.210	0.180	0.143	0.128	0.099	0.091	0.083	0.045
27°C	0.368	0.323	0.284	0.264	0.195	0.167	0.152	0.147	0.084
29°C	0.460	0.333	0.499	0.261	0.320	0.161	0.127	0.161	0.052
31°C	0.575	0.439	0.386	0.330	0.307	0.235	0.252	0.146	0.067
33°C	0.539	0.482	0.385	0.301	0.290	0.229	0.212	0.208	0.114
35°C	0.661	0.572	0.469	0.296	0.267	0.230	0.203	0.171	0.122
37°C	0.606	0.515	0.356	0.365	0.256	0.188	0.137	0.146	0.076
39°C	0.588	0.437	0.313	0.318	0.230	0.308	0.298	0.114	0.271
41°C	0.551	0.659	0.550	0.445	0.442	0.180	0.164	0.175	0.080
43°C	0.546	0.494	0.341	0.267	0.510	0.195	0.146	0.151	0.082

Figure S2: Relative heights (with vs. without liposomes) of peaks in aSyn 2D 1H-15N HSQC spectra at various temperatures.

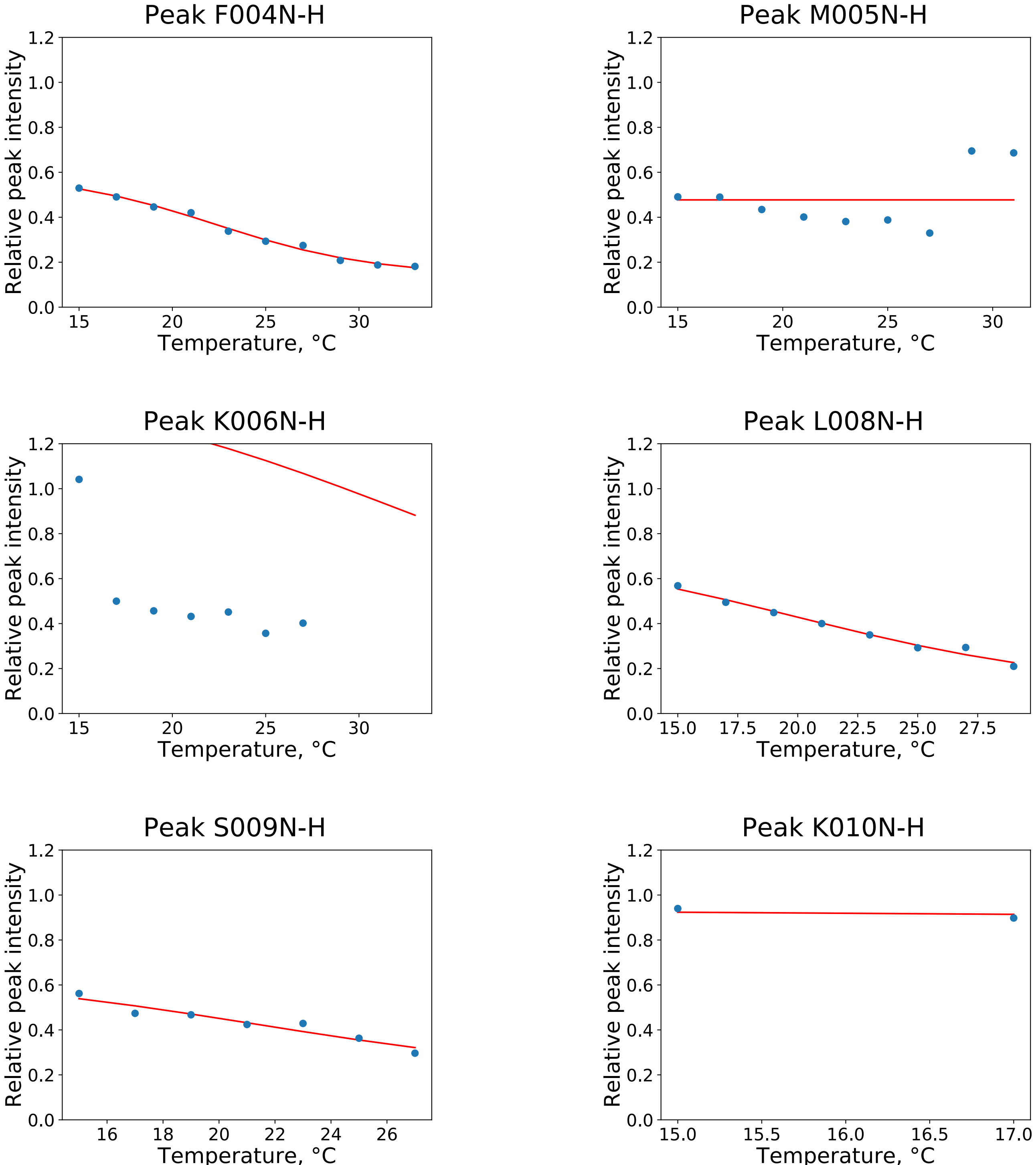
Peak F004N-H
Relative peak intensity
Temperature, °C
Peak M005N-H
Relative peak intensity
Temperature, °C
Peak K006N-H
Relative peak intensity
Temperature, °C
Peak L008N-H
Relative peak intensity
Temperature, °C
Peak S009N-H
Relative peak intensity
Temperature, °C
Peak K010N-H
Relative peak intensity
Temperature, °C

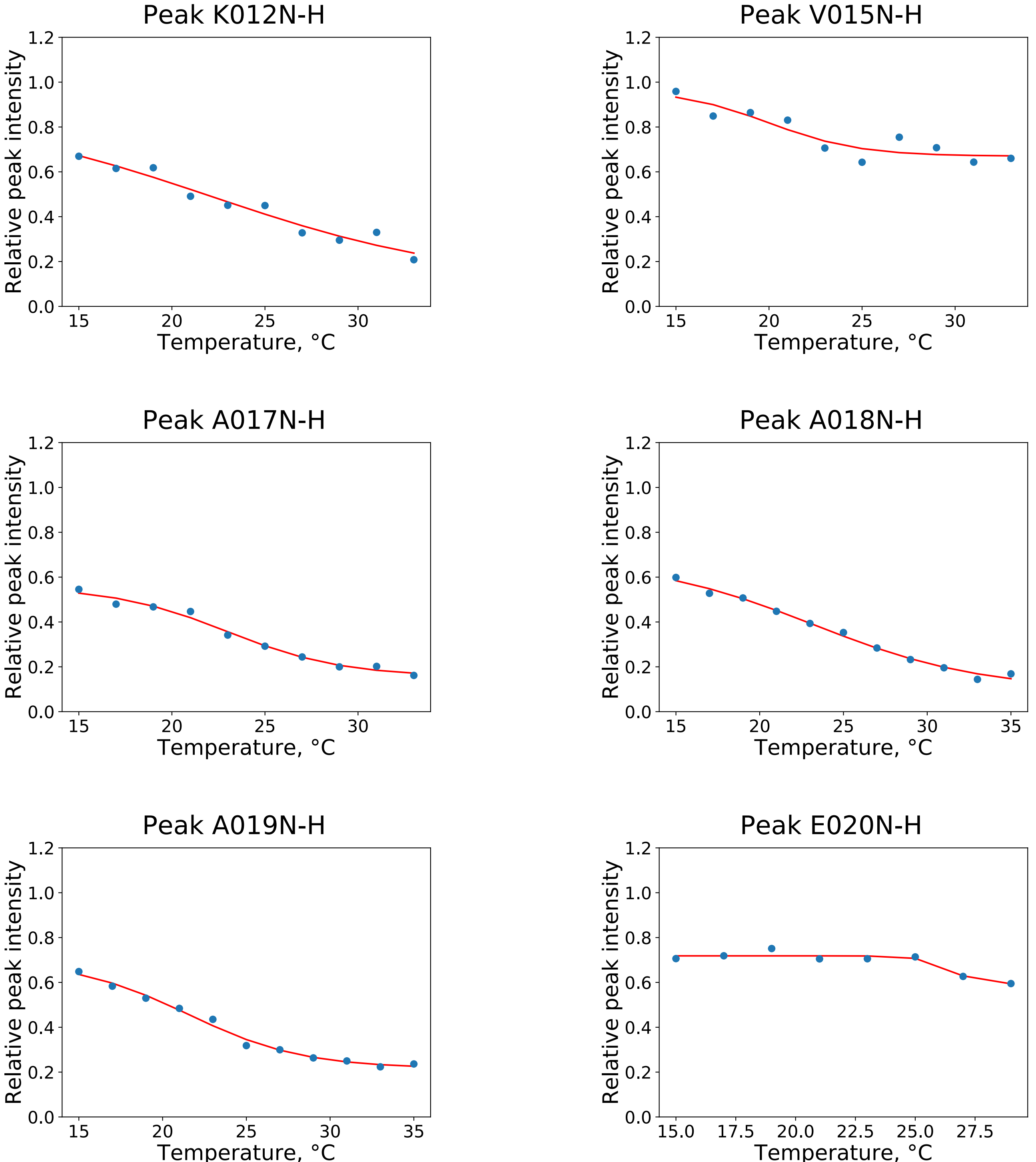

Peak K012N-H
Peak V015N-H
Peak A017N-H
Peak A018N-H
Peak A019N-H
Peak E020N-H
Relative peak intensity
Temperature, °C
0.0
0.2
0.4
0.6
0.8
1.0
1.2
15
20
25
30
35
15.0
17.5
20.0
22.5
25.0
27.5

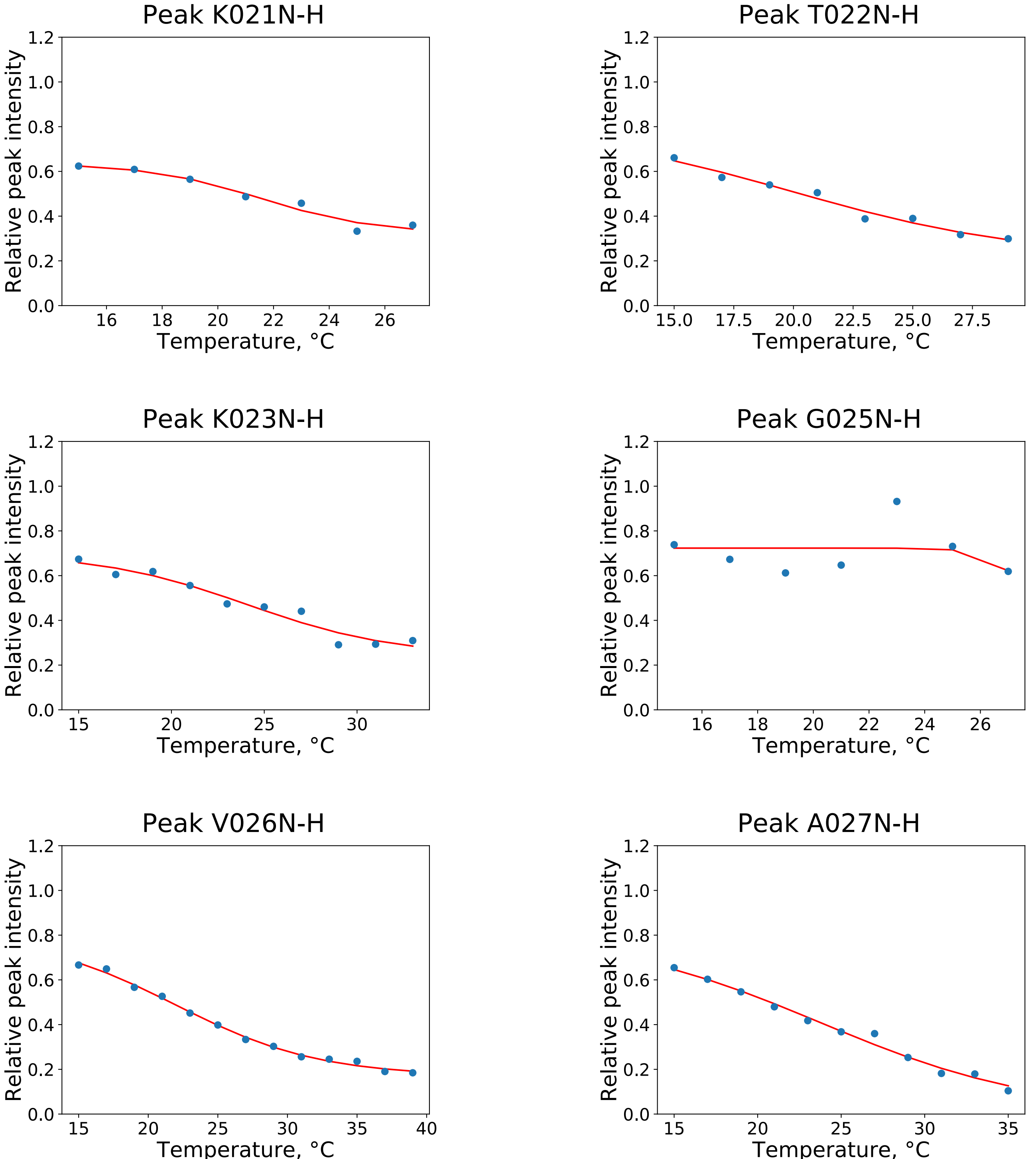

Peak K021N-H
Relative peak intensity
0.0 0.2 0.4 0.6 0.8 1.0 1.2
16 18 20 22 24 26
Temperature, °C
Peak T022N-H
Relative peak intensity
0.0 0.2 0.4 0.6 0.8 1.0 1.2
15.0 17.5 20.0 22.5 25.0 27.5
Temperature, °C
Peak K023N-H
Relative peak intensity
0.0 0.2 0.4 0.6 0.8 1.0 1.2
15 20 25 30
Temperature, °C
Peak G025N-H
Relative peak intensity
0.0 0.2 0.4 0.6 0.8 1.0 1.2
16 18 20 22 24 26
Temperature, °C
Peak V026N-H
Relative peak intensity
0.0 0.2 0.4 0.6 0.8 1.0 1.2
15 20 25 30 35 40
Temperature, °C
Peak A027N-H
Relative peak intensity
0.0 0.2 0.4 0.6 0.8 1.0 1.2
15 20 25 30 35
Temperature, °C

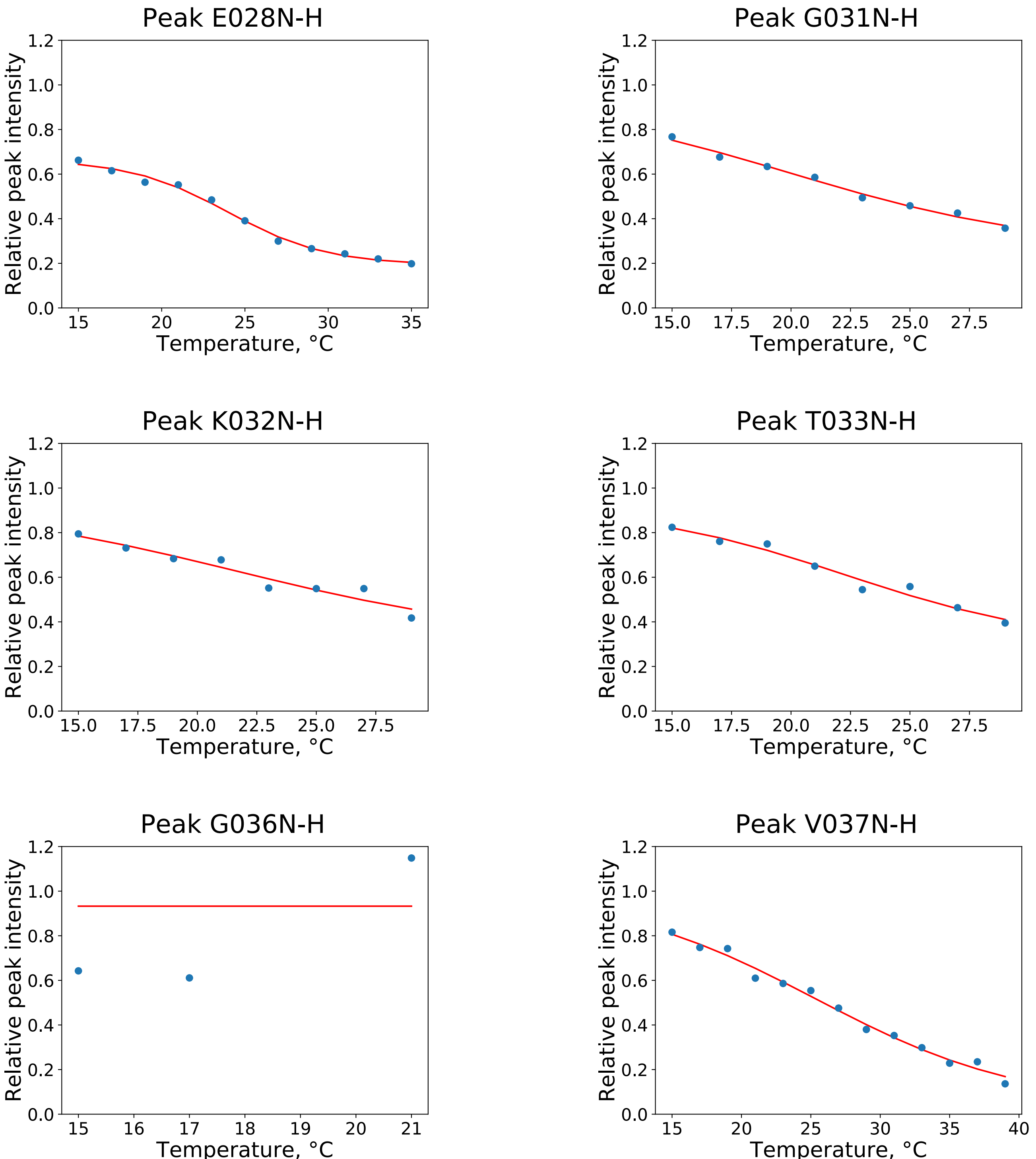
Peak E028N-H
Peak G031N-H
Peak K032N-H
Peak T033N-H
Peak G036N-H
Peak V037N-H
Relative peak intensity
Temperature, °C

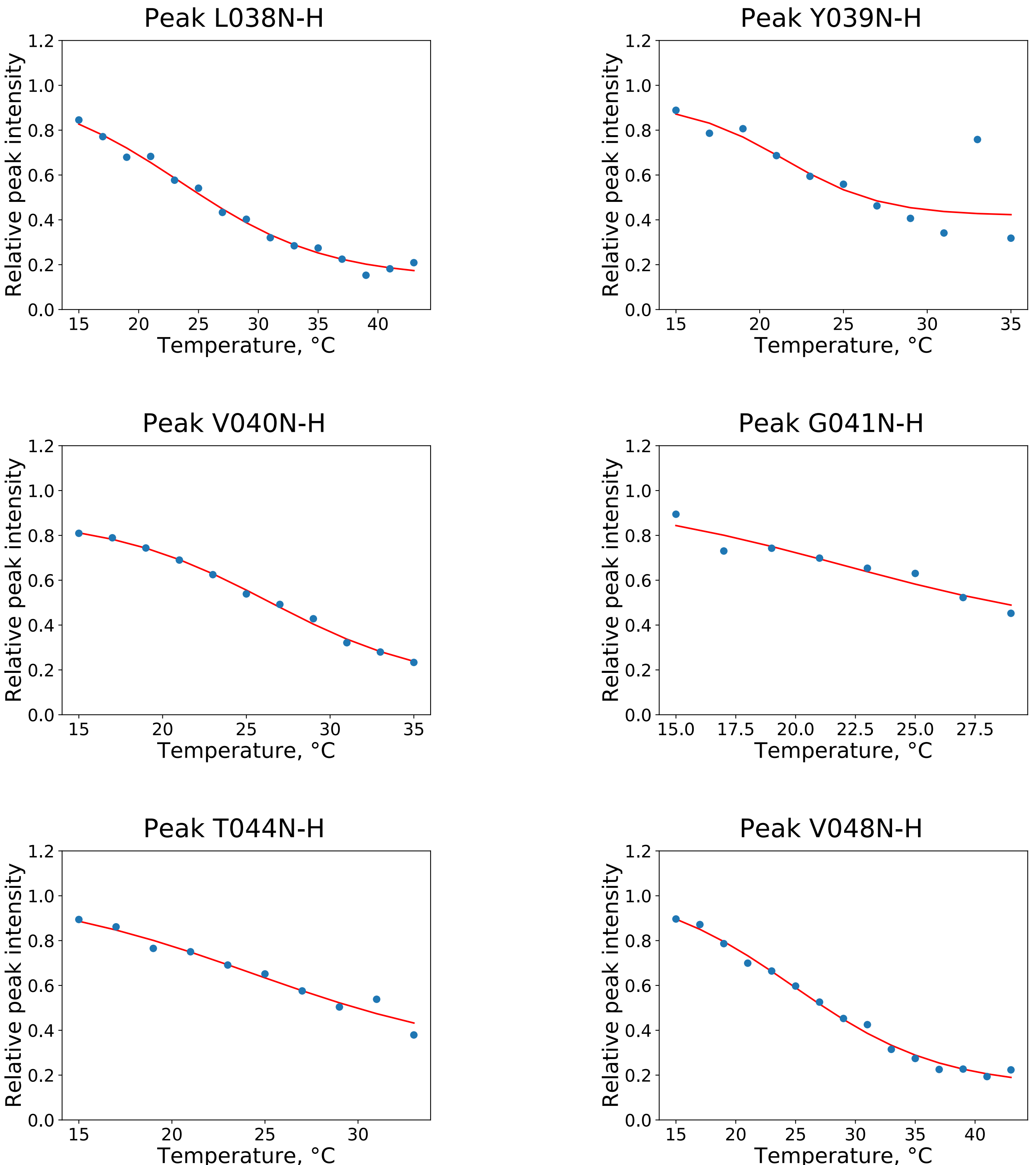
Peak L038N-H
Relative peak intensity
Temperature, °C
Peak Y039N-H
Relative peak intensity
Temperature, °C
Peak V040N-H
Relative peak intensity
Temperature, °C
Peak G041N-H
Relative peak intensity
Temperature, °C
Peak T044N-H
Relative peak intensity
Temperature, °C
Peak V048N-H
Relative peak intensity
Temperature, °C

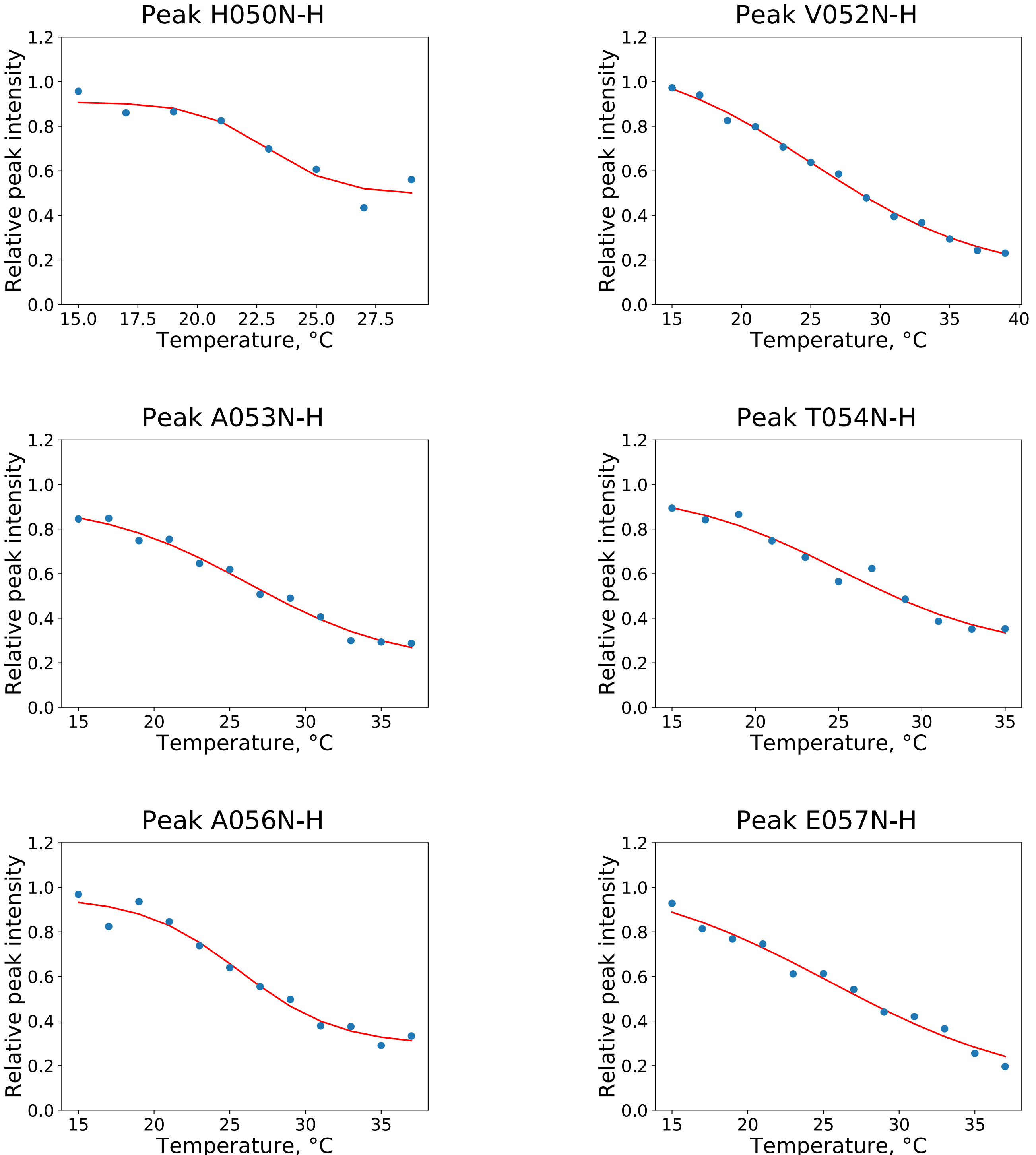
Peak H050N-H
Relative peak intensity
Temperature, °C
0.0 0.2 0.4 0.6 0.8 1.0 1.2
15.0 17.5 20.0 22.5 25.0 27.5
Peak V052N-H
Relative peak intensity
Temperature, °C
0.0 0.2 0.4 0.6 0.8 1.0 1.2
15 20 25 30 35 40
Peak A053N-H
Relative peak intensity
Temperature, °C
0.0 0.2 0.4 0.6 0.8 1.0 1.2
15 20 25 30 35
Peak T054N-H
Relative peak intensity
Temperature, °C
0.0 0.2 0.4 0.6 0.8 1.0 1.2
15 20 25 30 35
Peak A056N-H
Relative peak intensity
Temperature, °C
0.0 0.2 0.4 0.6 0.8 1.0 1.2
15 20 25 30 35
Peak E057N-H
Relative peak intensity
Temperature, °C
0.0 0.2 0.4 0.6 0.8 1.0 1.2
15 20 25 30 35

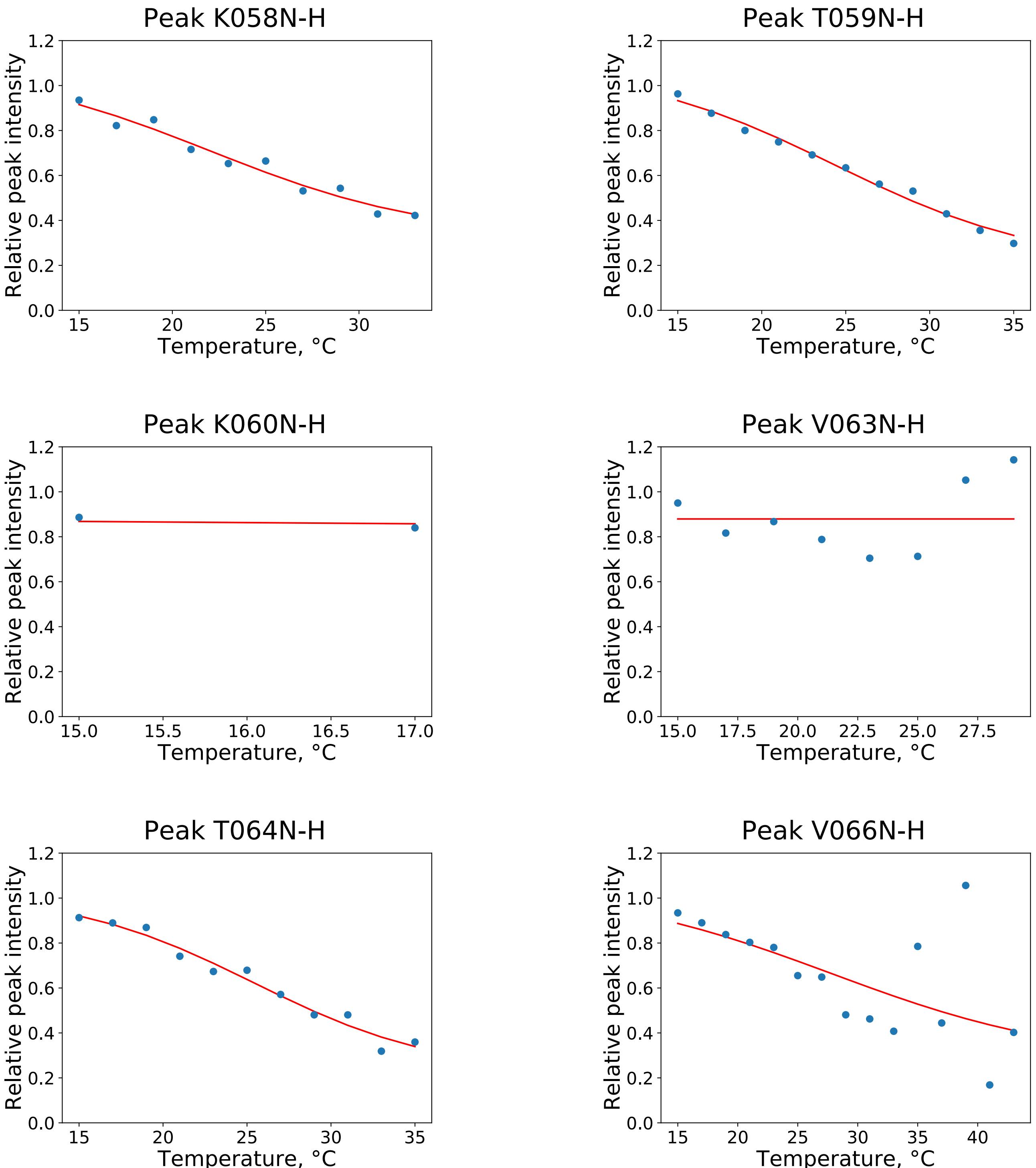
Peak K058N-H
Peak T059N-H
Peak K060N-H
Peak V063N-H
Peak T064N-H
Peak V066N-H
Relative peak intensity
Temperature, °C
0.0
0.2
0.4
0.6
0.8
1.0
1.2
15
20
25
30
35
40
15.0
15.5
16.0
16.5
17.0
17.5
20.0
22.5
25.0
27.5

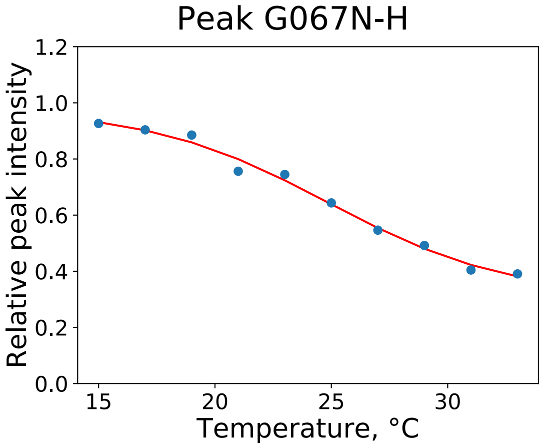
Peak G067N-H
Relative peak intensity
0.0 0.2 0.4 0.6 0.8 1.0 1.2
15 20 25 30
Temperature, °C

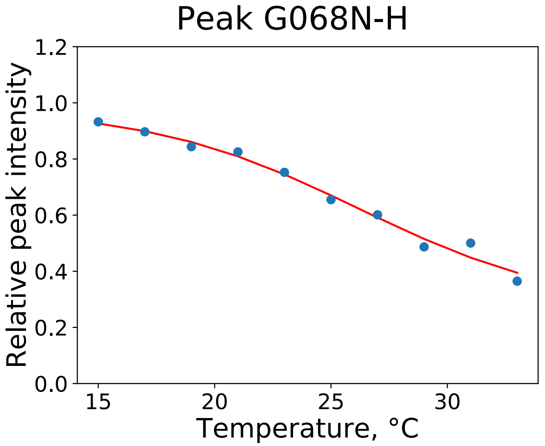
Peak G068N-H
Relative peak intensity
0.0 0.2 0.4 0.6 0.8 1.0 1.2
15 20 25 30
Temperature, °C

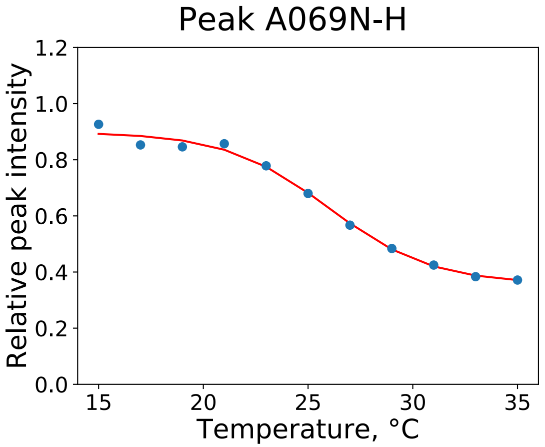
Peak A069N-H
Relative peak intensity
0.0 0.2 0.4 0.6 0.8 1.0 1.2
15 20 25 30 35
Temperature, °C

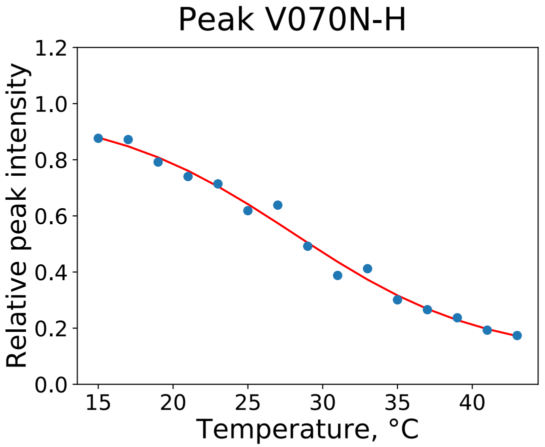
Peak V070N-H
Relative peak intensity
0.0 0.2 0.4 0.6 0.8 1.0 1.2
15 20 25 30 35 40
Temperature, °C

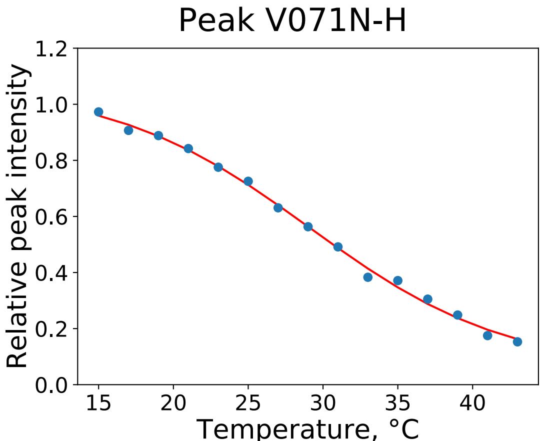
Peak V071N-H
Relative peak intensity
0.0 0.2 0.4 0.6 0.8 1.0 1.2
15 20 25 30 35 40
Temperature, °C

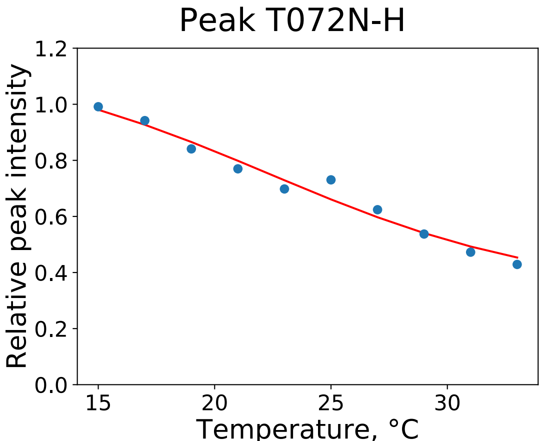
Peak T072N-H
Relative peak intensity
0.0 0.2 0.4 0.6 0.8 1.0 1.2
15 20 25 30
Temperature, °C

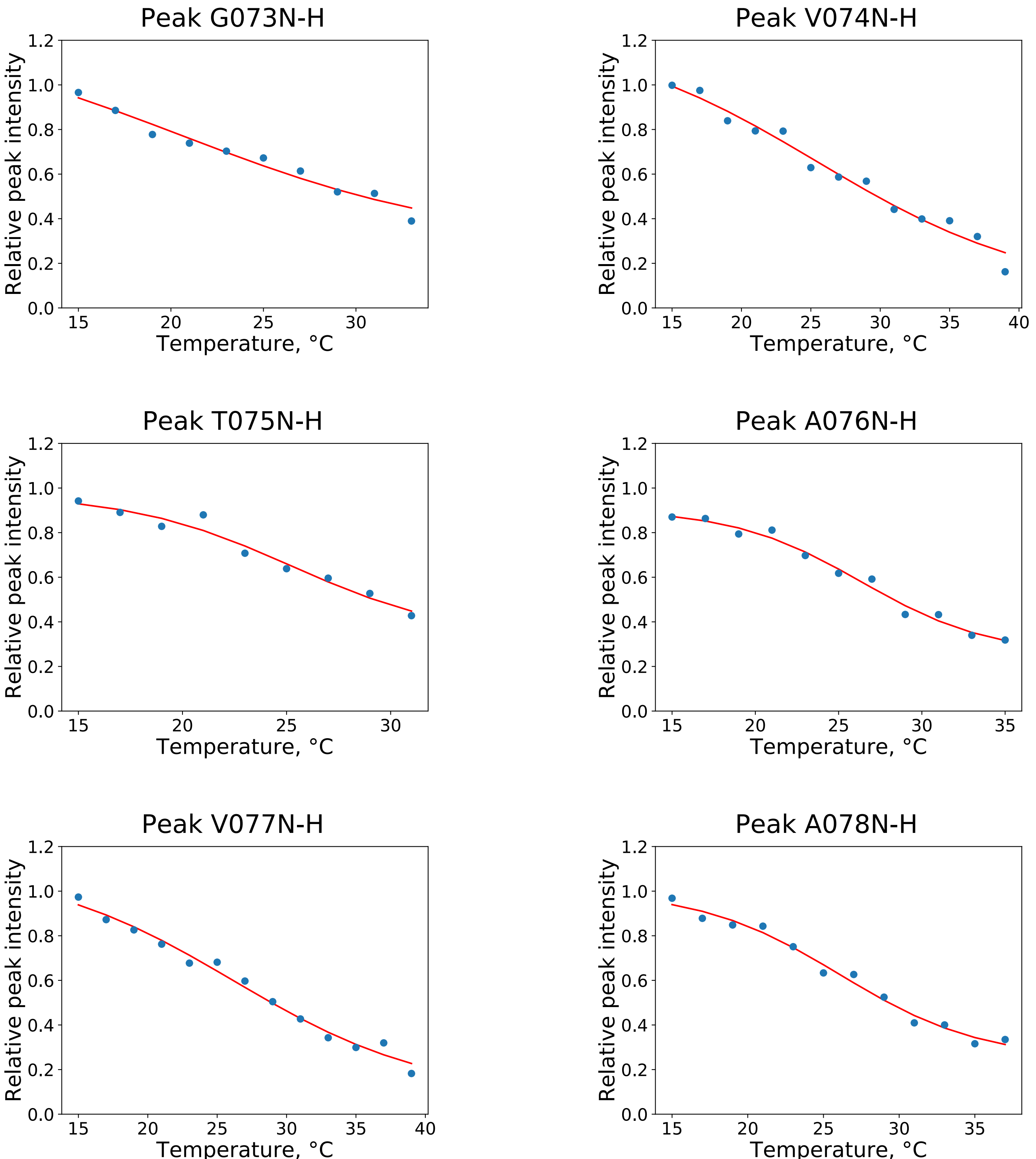
Peak G073N-H
Peak V074N-H
Peak T075N-H
Peak A076N-H
Peak V077N-H
Peak A078N-H
Relative peak intensity
Temperature, °C

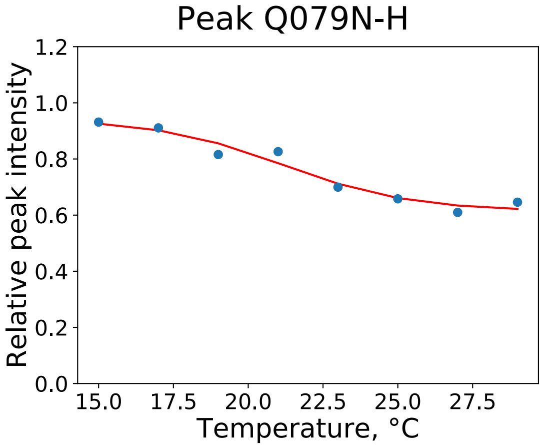
Peak Q079N-H
Relative peak intensity
Temperature, °C
0.0
0.2
0.4
0.6
0.8
1.0
1.2
15.0
17.5
20.0
22.5
25.0
27.5

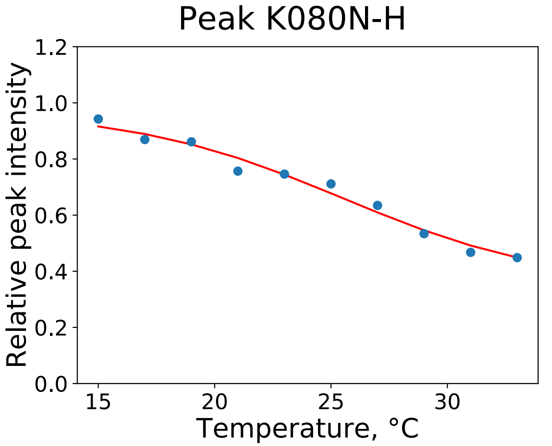
Peak K080N-H
Relative peak intensity
Temperature, °C
0.0
0.2
0.4
0.6
0.8
1.0
1.2
15
20
25
30

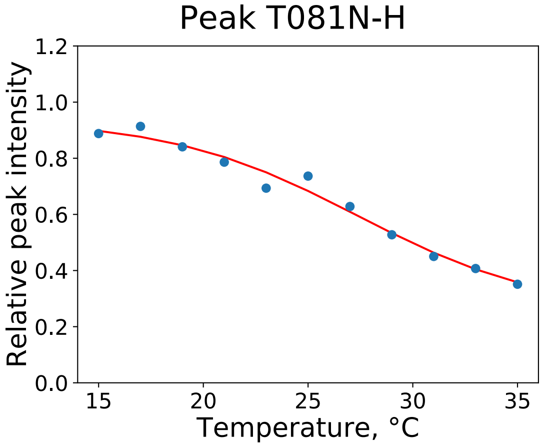
Peak T081N-H
Relative peak intensity
Temperature, °C
0.0
0.2
0.4
0.6
0.8
1.0
1.2
15
20
25
30
35

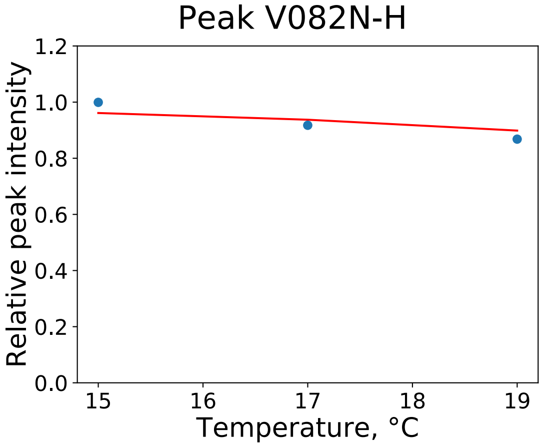
Peak V082N-H
Relative peak intensity
Temperature, °C
0.0
0.2
0.4
0.6
0.8
1.0
1.2
15
16
17
18
19

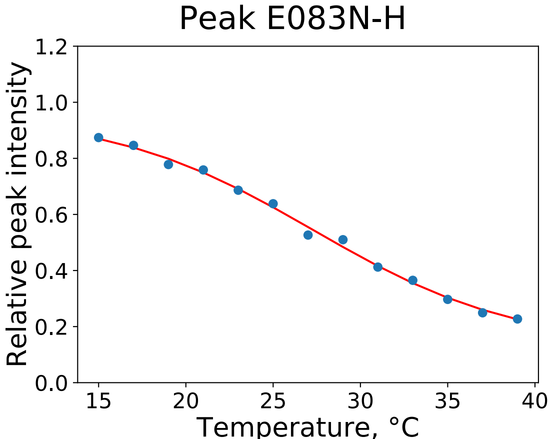
Peak E083N-H
Relative peak intensity
Temperature, °C
0.0
0.2
0.4
0.6
0.8
1.0
1.2
15
20
25
30
35
40

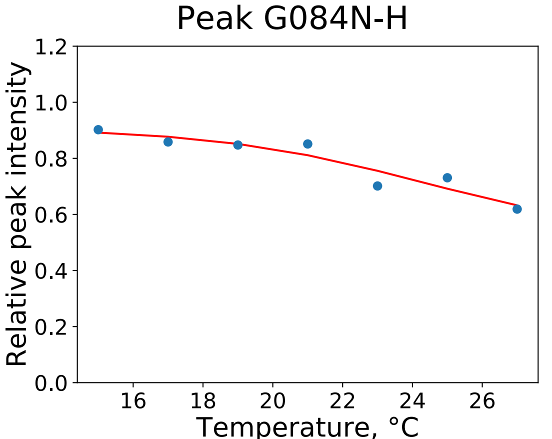
Peak G084N-H
Relative peak intensity
Temperature, °C
0.0
0.2
0.4
0.6
0.8
1.0
1.2
16
18
20
22
24
26

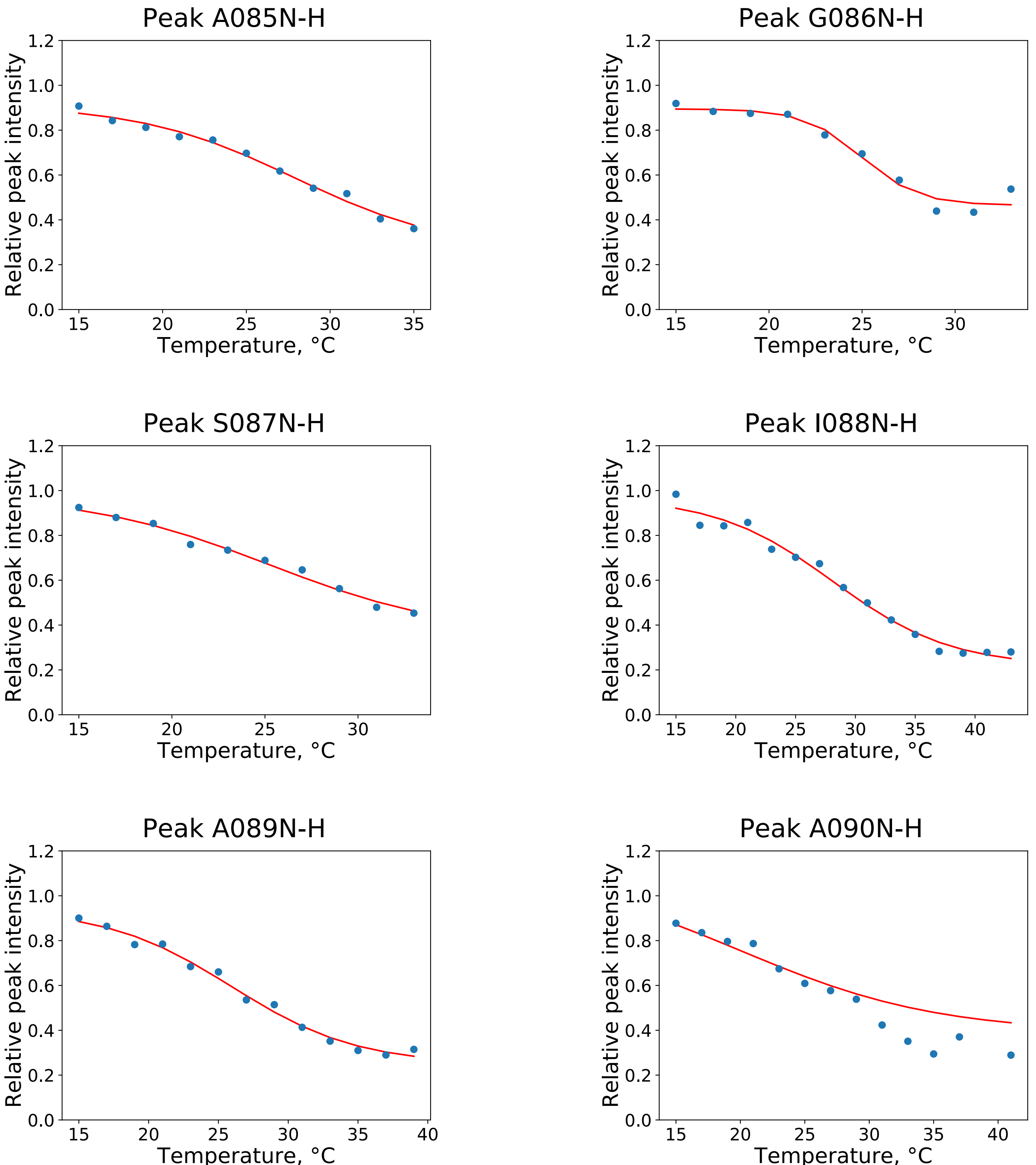
Peak A085N-H
Peak G086N-H
Peak S087N-H
Peak I088N-H
Peak A089N-H
Peak A090N-H
Relative peak intensity
Temperature, °C

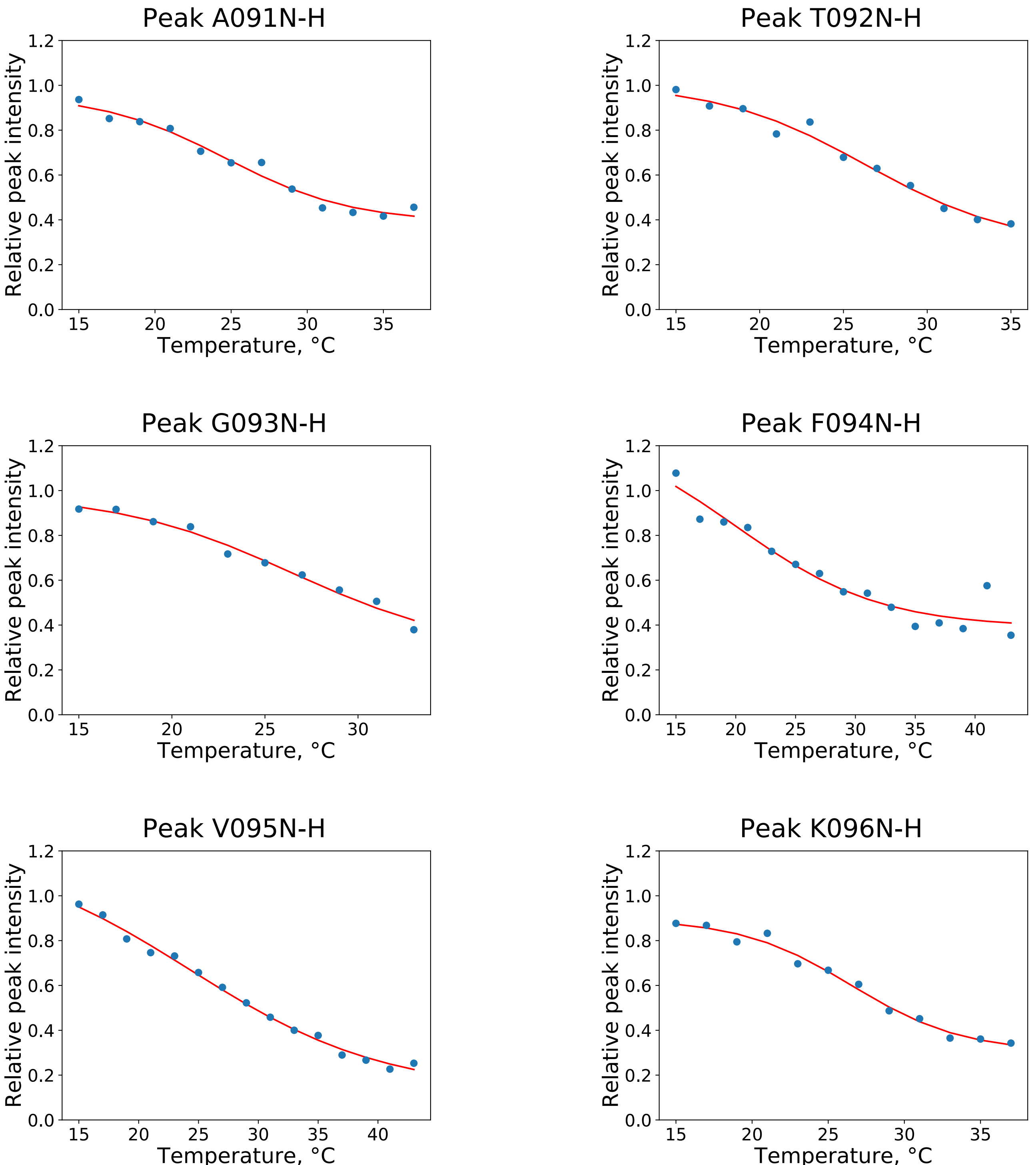
Peak A091N-H
Peak T092N-H
Peak G093N-H
Peak F094N-H
Peak V095N-H
Peak K096N-H
Relative peak intensity
Temperature, °C
0.0
0.2
0.4
0.6
0.8
1.0
1.2
15
20
25
30
35
40

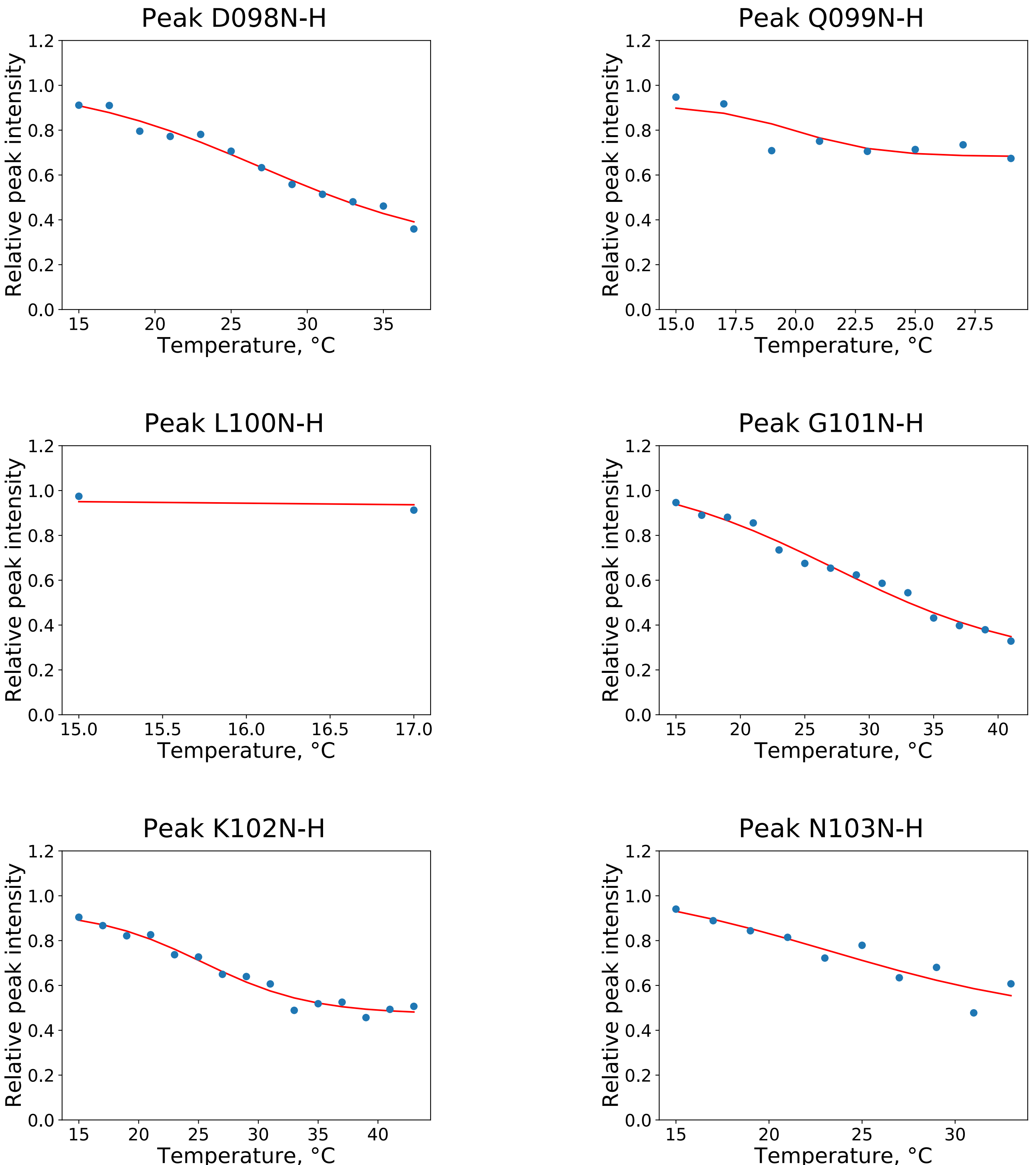
Peak D098N-H
Relative peak intensity
0.0 0.2 0.4 0.6 0.8 1.0 1.2
15 20 25 30 35
Temperature, °C
Peak Q099N-H
Relative peak intensity
0.0 0.2 0.4 0.6 0.8 1.0 1.2
15.0 17.5 20.0 22.5 25.0 27.5
Temperature, °C
Peak L100N-H
Relative peak intensity
0.0 0.2 0.4 0.6 0.8 1.0 1.2
15.0 15.5 16.0 16.5 17.0
Temperature, °C
Peak G101N-H
Relative peak intensity
0.0 0.2 0.4 0.6 0.8 1.0 1.2
15 20 25 30 35 40
Temperature, °C
Peak K102N-H
Relative peak intensity
0.0 0.2 0.4 0.6 0.8 1.0 1.2
15 20 25 30 35 40
Temperature, °C
Peak N103N-H
Relative peak intensity
0.0 0.2 0.4 0.6 0.8 1.0 1.2
15 20 25 30
Temperature, °C

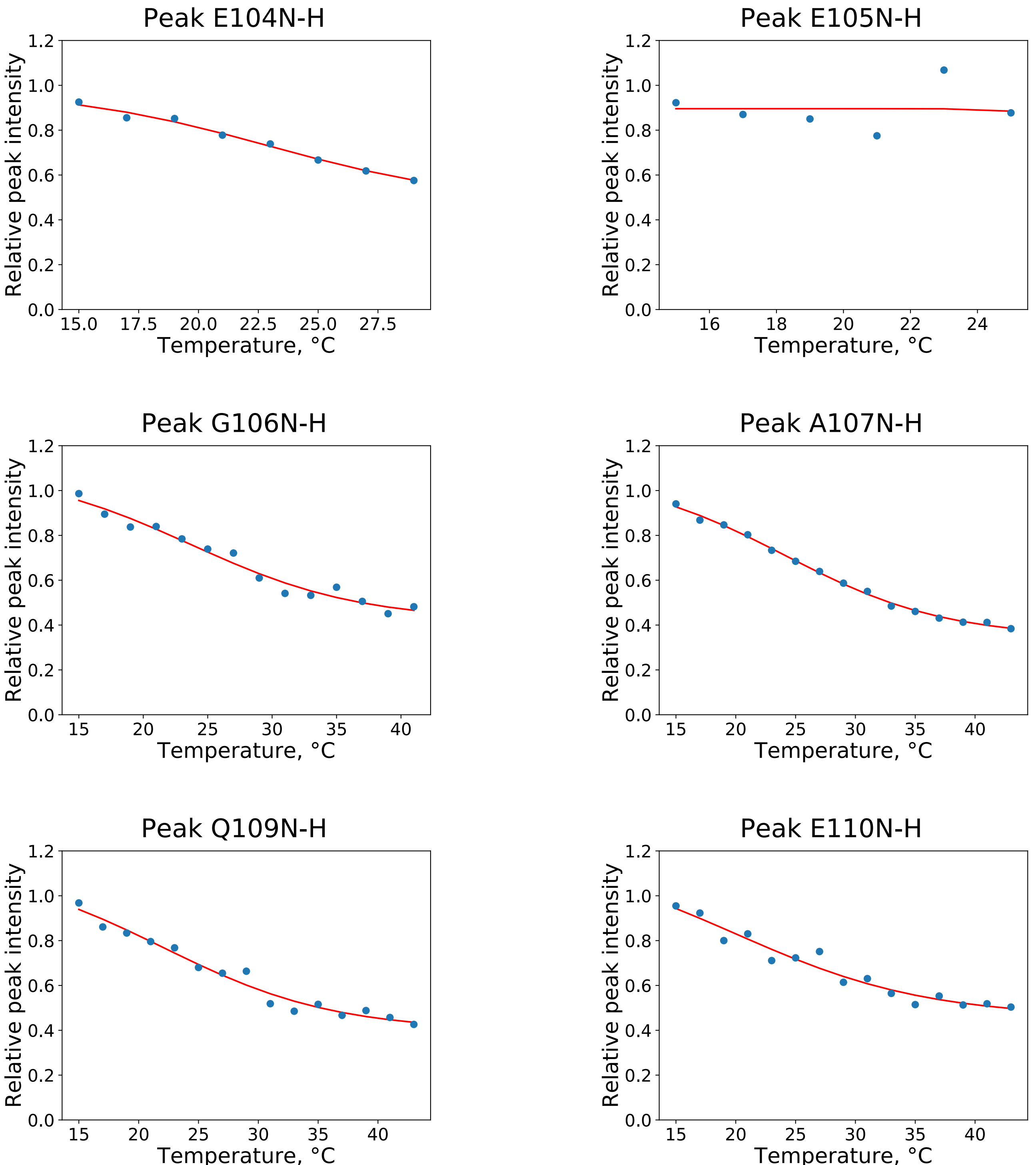
Peak E104N-H
Relative peak intensity
Temperature, °C
0.0
0.2
0.4
0.6
0.8
1.0
1.2
15.0
17.5
20.0
22.5
25.0
27.5
Peak E105N-H
16
18
20
22
24
Peak G106N-H
15
20
25
30
35
40
Peak A107N-H
Peak Q109N-H
Peak E110N-H

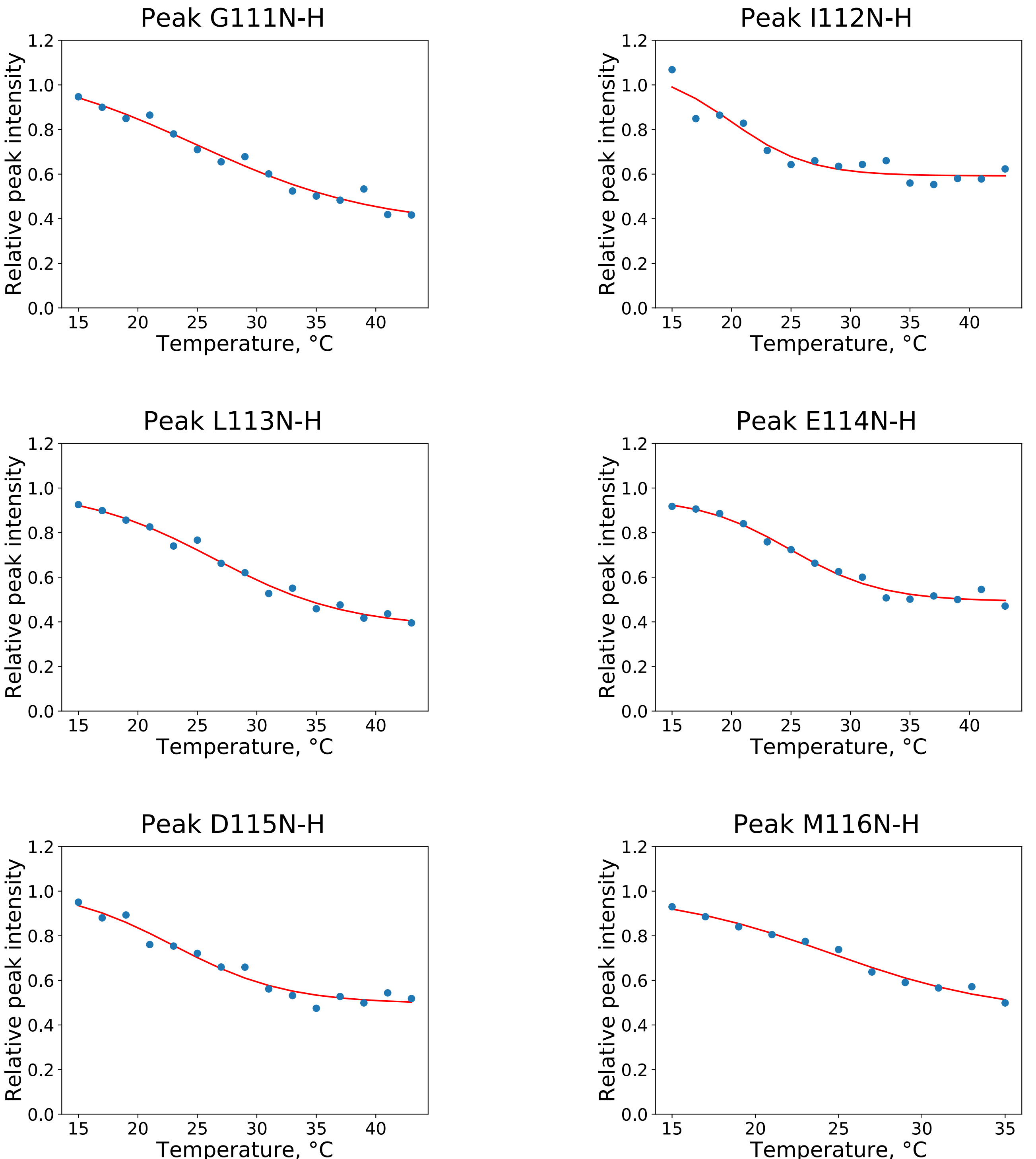
Peak G111N-H
Peak I112N-H
Peak L113N-H
Peak E114N-H
Peak D115N-H
Peak M116N-H
Relative peak intensity
Temperature, °C
0.0
0.2
0.4
0.6
0.8
1.0
1.2
15
20
25
30
35
40

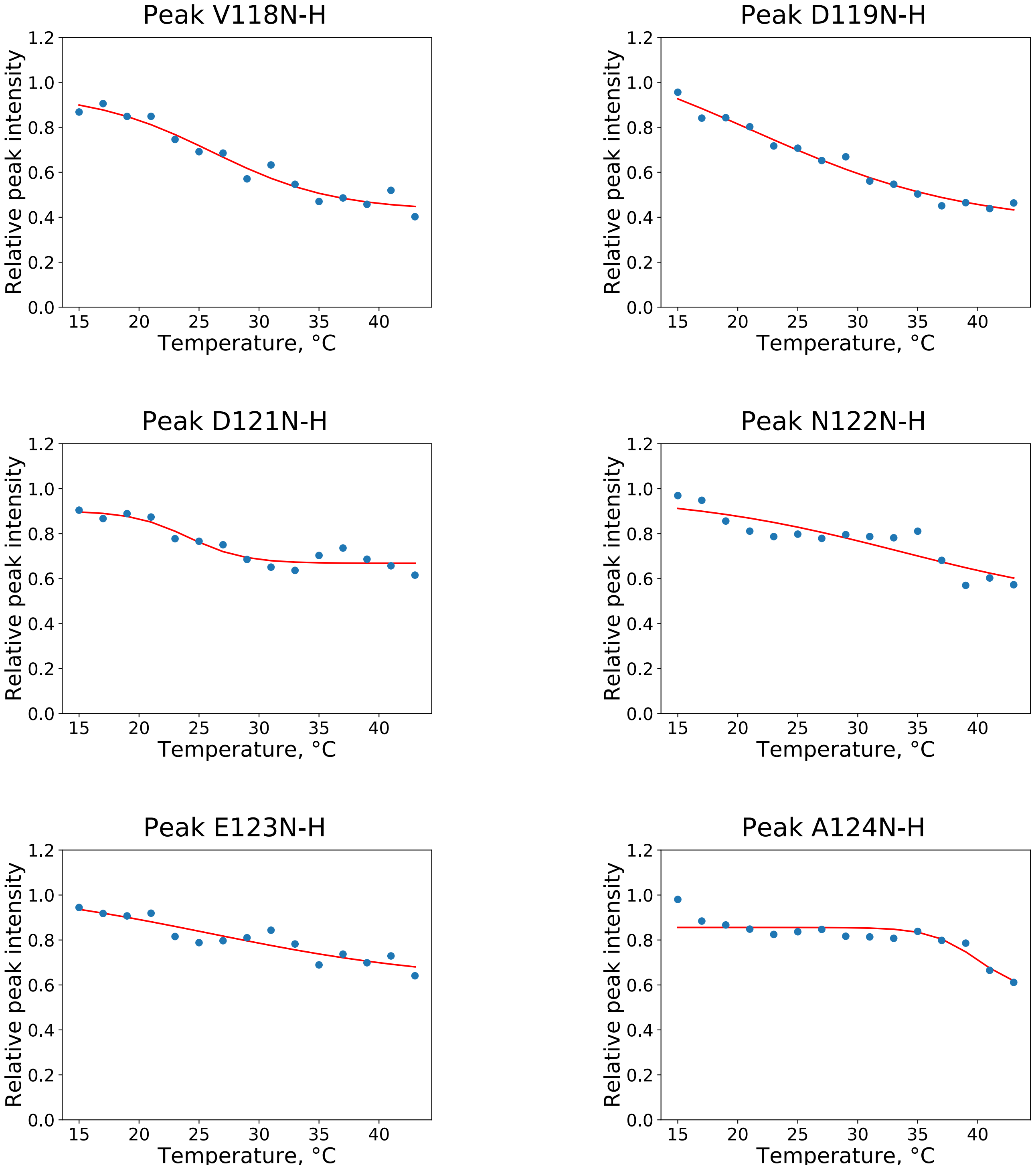
Peak V118N-H
Peak D119N-H
Peak D121N-H
Peak N122N-H
Peak E123N-H
Peak A124N-H
Relative peak intensity
Temperature, °C
0.0
0.2
0.4
0.6
0.8
1.0
1.2
15
20
25
30
35
40

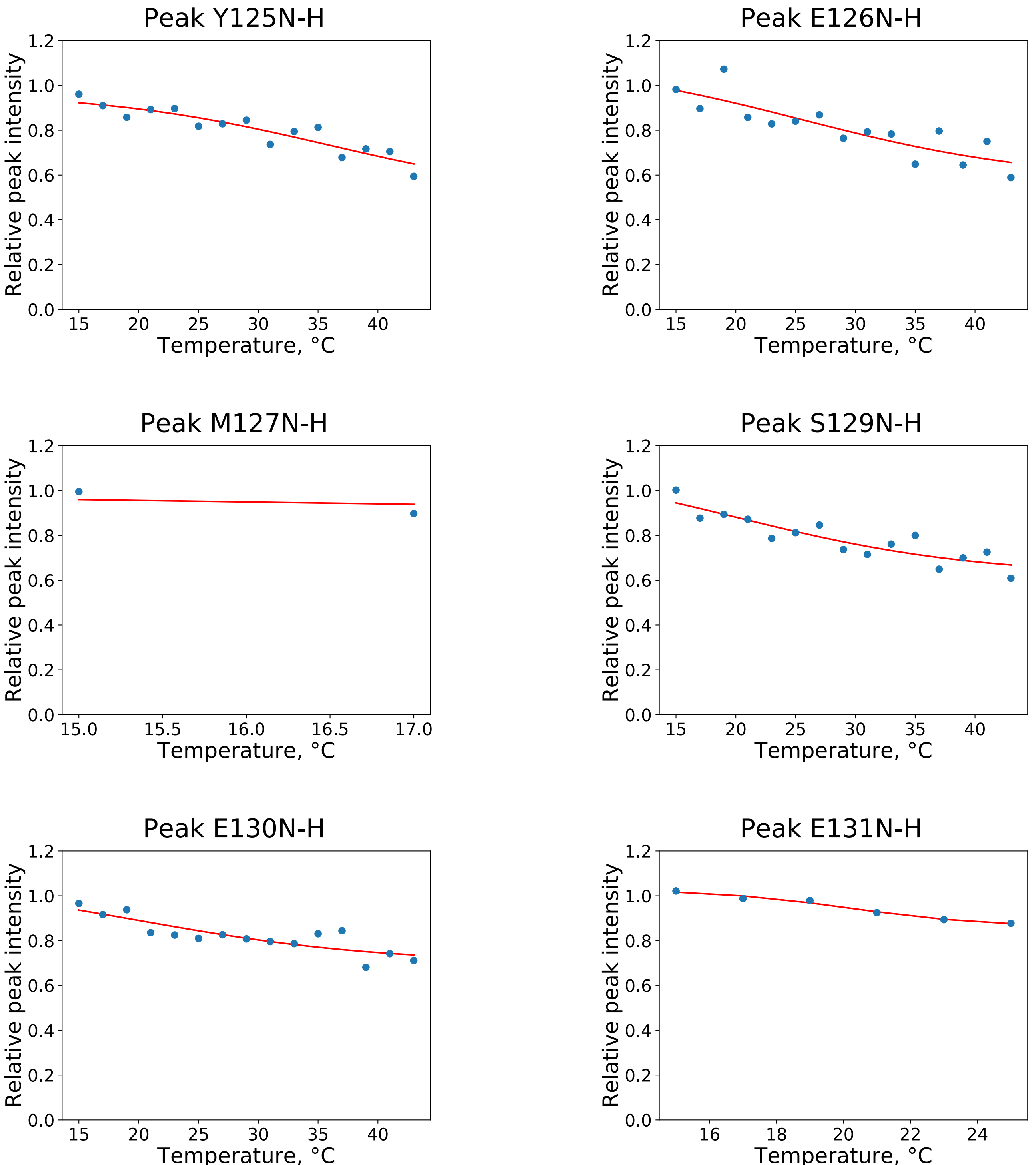
Peak Y125N-H
Peak E126N-H
Peak M127N-H
Peak S129N-H
Peak E130N-H
Peak E131N-H
Relative peak intensity
Temperature, °C

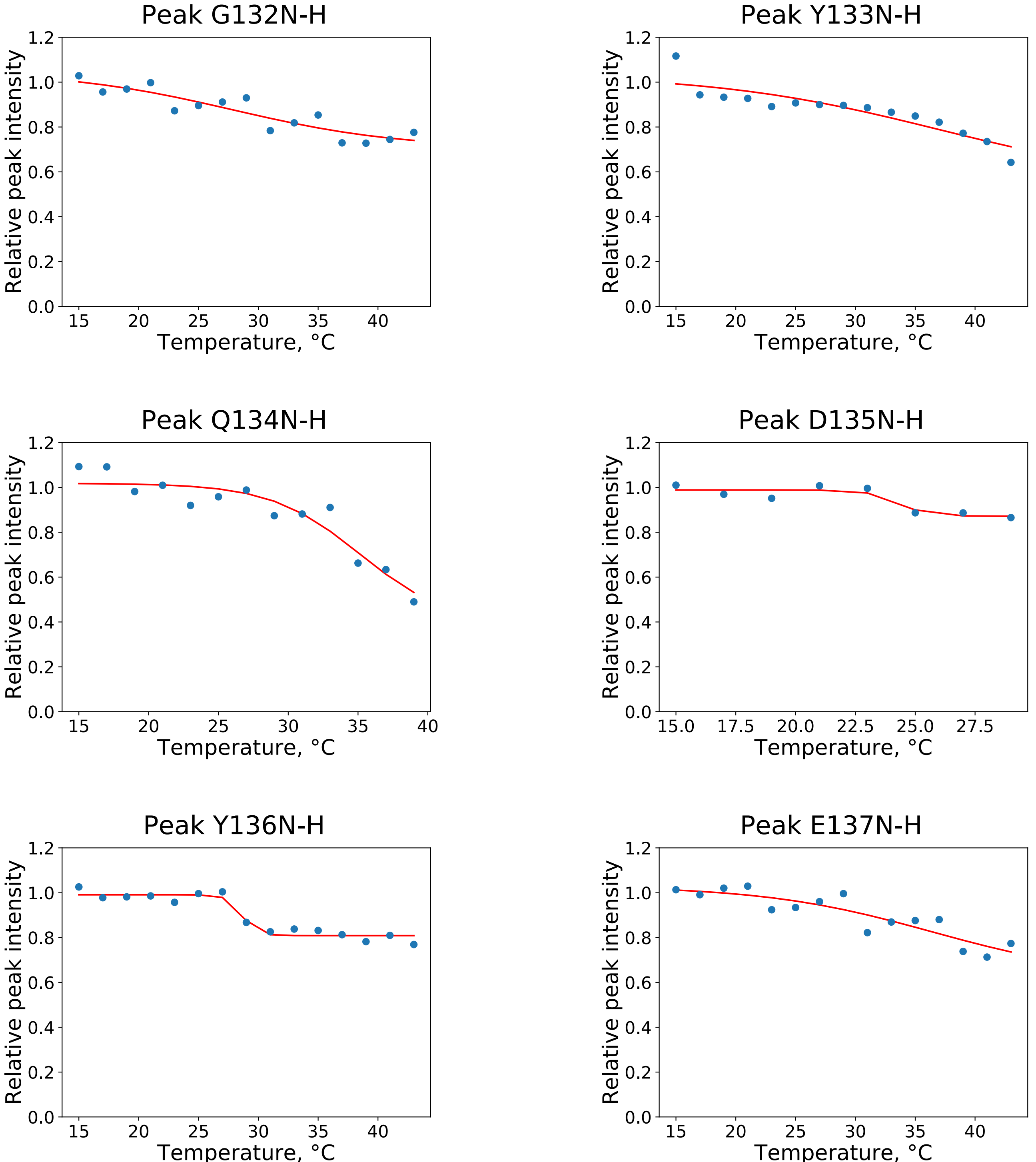

Peak G132N-H
Peak Y133N-H
Peak Q134N-H
Peak D135N-H
Peak Y136N-H
Peak E137N-H
Relative peak intensity
Temperature, °C
0.0
0.2
0.4
0.6
0.8
1.0
1.2
15
20
25
30
35
40
15.0
17.5
20.0
22.5
25.0
27.5

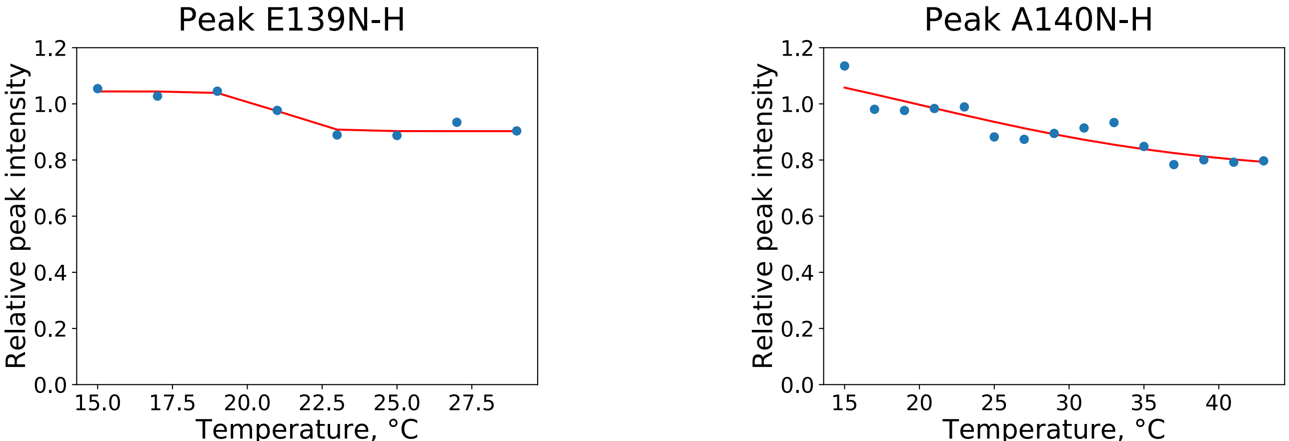

Peak E139N-H
Relative peak intensity
Temperature, °C
1.2
1.0
0.8
0.6
0.4
0.2
0.0
15.0
17.5
20.0
22.5
25.0
27.5
Peak A140N-H
15
20
25
30
35
40
